# Air‐Insensitive Sulfonylation Enabled by a MOF‐Supported Nickel Photocatalyst

**DOI:** 10.1002/smll.202508991

**Published:** 2025-09-27

**Authors:** Guannan Wang, Tianyu Liu, Ching Kit Tommy Wun, Tsz Woon Benedict Lo, Jian He

**Affiliations:** ^1^ Department of Chemistry The University of Hong Kong Pokfulam Road Hong Kong 999077 P. R. China; ^2^ State Key Laboratory of Synthetic Chemistry The University of Hong Kong Pokfulam Road Hong Kong 999077 P. R. China; ^3^ Materials Innovation Institute for Life Sciences and Energy (MILES) HKU‐SIRI Shenzhen 518063 P. R. China; ^4^ State Key Laboratory of Chemical Biology and Drug Discovery Department of Applied Biology and Chemical Technology The Hong Kong Polytechnic University Hong Kong 999077 P. R. China

**Keywords:** heterogeneous catalysis, metal–organic frameworks, nickel, photocatalysis, sustainable synthesis

## Abstract

Photoredox nickel dual catalysis has become a powerful tool in organic synthesis over the past decade, demonstrating versatile reactivity and utilizing earth‐abundant metals to form new bonds. However, the requirement for strict inert‐gas protection and the existence of intricate metal complex equilibria in homogeneous systems present obstacles to further advancement in practical applications and mechanistic studies. Herein, a heterogeneous strategy is devised by immobilizing nickel complexes within a highly crystalline mesoporous framework, enabling the photoinduced sulfonylation of aryl halides to proceed under ambient air conditions. Notably, pre‐treatment with coordinating solvents significantly enhances the activity of the framework‐supported nickel catalyst. This research not only broadens the scope of sustainable cross‐coupling methodologies but also provides valuable insights into designing robust heterogeneous photocatalytic systems.

## Graphical abstract

Immobilizing a nickel complex coordinated with a specially designed bidentate ligand into a stable zirconium‐based metal–organic framework establishes a heterogeneous platform for promoting air‐insensitive sulfonylation of aryl halides. In this system, solvent coordination is found to enhance C─S bond formation, a process that is challenging to investigate in homogeneous transition‐metal catalysis.

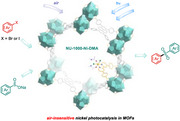

## 1 Introduction

**Figure 1:**
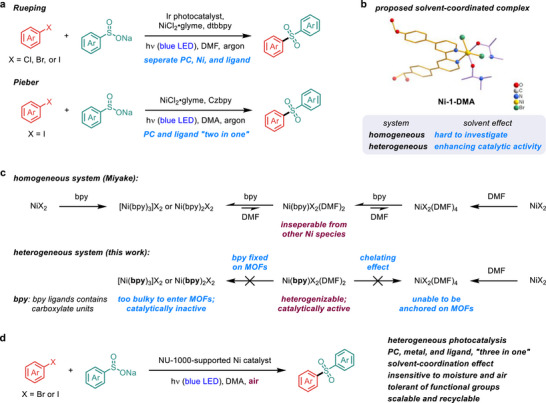
a) Catalyst design for homogeneous nickel‐catalyzed sulfonylation of aryl halides under induced by visible light. Czbpy, 5,5′‐di(9H‐carbazol‐9‐yl)‐2,2′‐bipyridine; dtbbpy, dtbbpy, 4,4′‐di‐tert‐butyl‐2,2′‐bipyridine; glyme, 1,2‐dimethoxyethane. b) Establishing a platform to explore solvent effects on a mono‐bpy‐ligated nickel complex to improve photocatalytic activity. c) Exclusive synthesis of the target nickel complex in coordinating solvents using a heterogenization approach. d) Sulfonylation with a NU‐1000‐supported nickel photocatalyst.

Transition‐metal‐catalyzed cross‐coupling reactions play a pivotal role in organic synthesis, offering efficient methodologies for the rapid formation of C─H and C─heteroatom bonds.^[^ ^1^, ^2^, ^3^, ^4^, ^5^, ^6^ ^]^ In recent years, the integration of transition‐metal catalysis with photoredox catalysis has emerged as a prominent research field.^[^ ^7^, ^8^ ^]^ This synergistic approach enables diverse transformations with broad functional‐group tolerance under mild conditions, facilitating easy access to a variety of diaryl sulfones—valuable scaffolds that exhibit significant bioactivity in drug discovery and versatile applications in synthetic chemistry.^[^ ^9^, ^10^ ^]^ In 2017, the Rueping group reported the pioneering work on the cross‐coupling of aryl halides with sodium sulfinates via nickel/photoredox dual catalysis.^[^ ^11^ ^]^ By employing a robust iridium complex as the photocatalyst (PC), the C─S coupling reaction proceeded efficiently with 10 mol% of a 2,2′‐bipyridine (bpy)‐ligated nickel species under argon atmosphere (**Figure** **1**a). Subsequent research has focused on developing bifunctional ligands that enable photoinduced nickel‐catalyzed cross‐coupling reactions without requiring external photocatalysts.^[^ ^12^, ^13^, ^14^ ^]^ Structural modifications to the bpy ligand, such as incorporating carbazole units^[^ ^13^ ^]^ or replacing a pyridine moiety with a quinoline group,^[^ ^14^ ^]^ have proven effective for the sulfonylation of aryl iodides in nickel photocatalysis. Despite significant advancements in homogeneous photocatalytic systems, there remains a strong need to develop highly efficient and recyclable nickel photocatalysts that can operate in the presence of ambient air during light exposure (Figure S1, Supporting Information),^[^ ^15^, ^16^, ^17^, ^18^, ^19^, ^20^, ^21^ ^]^ while demonstrating broad substrate compatibility for aryl halides in the practical synthesis of diaryl sulfones.

To streamline ligand preparation for enhanced synthetic practicality and establish a platform for studying solvent effects in nickel photocatalysis, we utilized a heterogenization strategy^[^ ^22^, ^23^, ^24^, ^25^, ^26^ ^]^ to anchor a bpy‐ligated nickel(II) complex onto metal–organic framework (MOF) supports through a benzoate linkage (Figure 1b).^[^ ^27^, ^28^, ^29^, ^30^ ^]^ In homogeneous systems, it is typically difficult to generate a mono‐bpy‐ligated nickel species even with stoichiometric ligand‐to‐metal ratios, particularly in coordinating solvents.^[^ ^31^ ^]^ The strong binding affinity of bpy ligands often leads to the formation of nickel complexes bound by multiple bpy ligands, along with solvent‐coordinated species, which complicates the identification of active nickel intermediates in photocatalytic reactions (Figure 1c). In contrast, the substantial steric bulk of multi‐bpy‐ligated nickel complexes prevents their incorporation into the framework pores, while nickel species lacking bpy coordination are eliminated during workup. Consequently, the heterogeneous system preferentially yields mono‐bpy‐ligated nickel complexes (Figure 1c), which offer open coordination sites for substrate and solvent binding,^[^ ^32^, ^33^, ^34^, ^35^ ^]^ thereby enhancing their photocatalytic performance.

In this work, we report a heterogeneous “three‐in‐one” catalytic system combining PC, metal, and ligand functions within the stable NU‐1000 framework^[^ ^36^, ^37^, ^38^, ^39^ ^]^ for visible‐light‐driven nickel‐catalyzed sulfonylation of aryl halides with sodium sulfinates (Figure 1d). The system displays exceptional functional group compatibility across diverse substrates. Capitalizing on the MOF's heterogeneous nature and confinement effects,^[^ ^40^, ^41^, ^42^ ^]^ the supported nickel photocatalyst exhibits remarkable stability, excellent recyclability, and gram‐scale applicability for diaryl sulfone synthesis. Remarkably, the protocol operates efficiently under ambient atmosphere, eliminating the need for stringent oxygen‐free conditions. Furthermore, this heterogeneous platform allows for the isolation and characterization of solvent‐coordinated nickel complexes, which we demonstrate contribute to enhanced catalytic performance.

## 2 Results and Discussion

**Figure 2:**
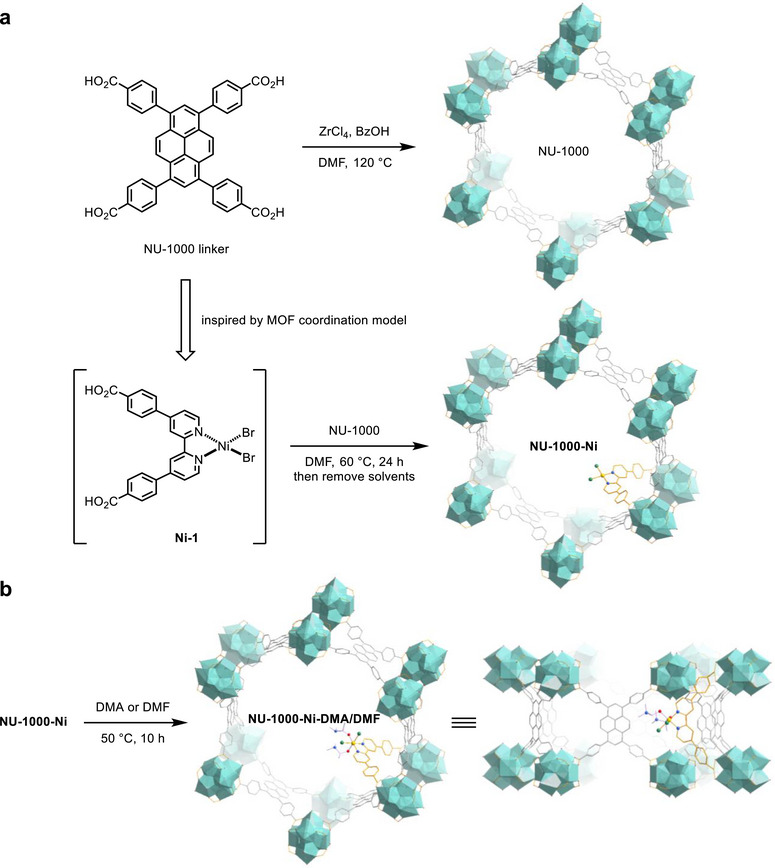
a) Integration of bpy‐ligated nickel complexes into NU‐1000‐derived framework supports. b) Boosting reactivity of heterogeneous photocatalysts via coordinating solvent modulation. Perspective viewed close to the [001] and [110] directions.

Synthesis and characterization of heterogeneous nickel photocatalysts. The zirconium‐based framework support, NU‐1000, was synthesized using a solvothermal method with benzoic acid as a modulator,^[^ ^43^, ^44^ ^]^ displaying rod‐shaped crystal structures as confirmed by scanning electron microscopy (SEM) images (Figure S5, Supporting Information). In an effort to enhance the interaction between functional linkers and MOF matrices to reduce metal leaching, a bpy‐ligated nickel(II) species, **Ni‐1**, designed to mimic the organic linkers of NU‐1000^[^ ^45^, ^46^ ^]^ and possessing two potential binding sites for the [Zr_6_] secondary building units, was successfully immobilized onto NU‐1000 through post‐synthetic metalation, resulting in **NU‐1000‐Ni** (**Figure** **2**a). The preservation of the morphology in **NU‐1000‐Ni** was verified through SEM images (Figure S5, Supporting Information), while energy‐dispersive X‐ray spectroscopy elemental mappings revealed the successful incorporation and uniform distribution of **Ni‐1** on NU‐1000, as indicated by the well‐dispersed signals of Ni and Br elements (Figures S6 and S7, Supporting Information). Immersing **NU‐1000‐Ni** in coordinating solvents, such as DMA and DMF, for 10 h led to the formation of solvent‐coordinated nickel(II) complexes on the framework support (Figure 2b), which were then employed in the evaluation of photocatalytic performance.

**Figure 3:**
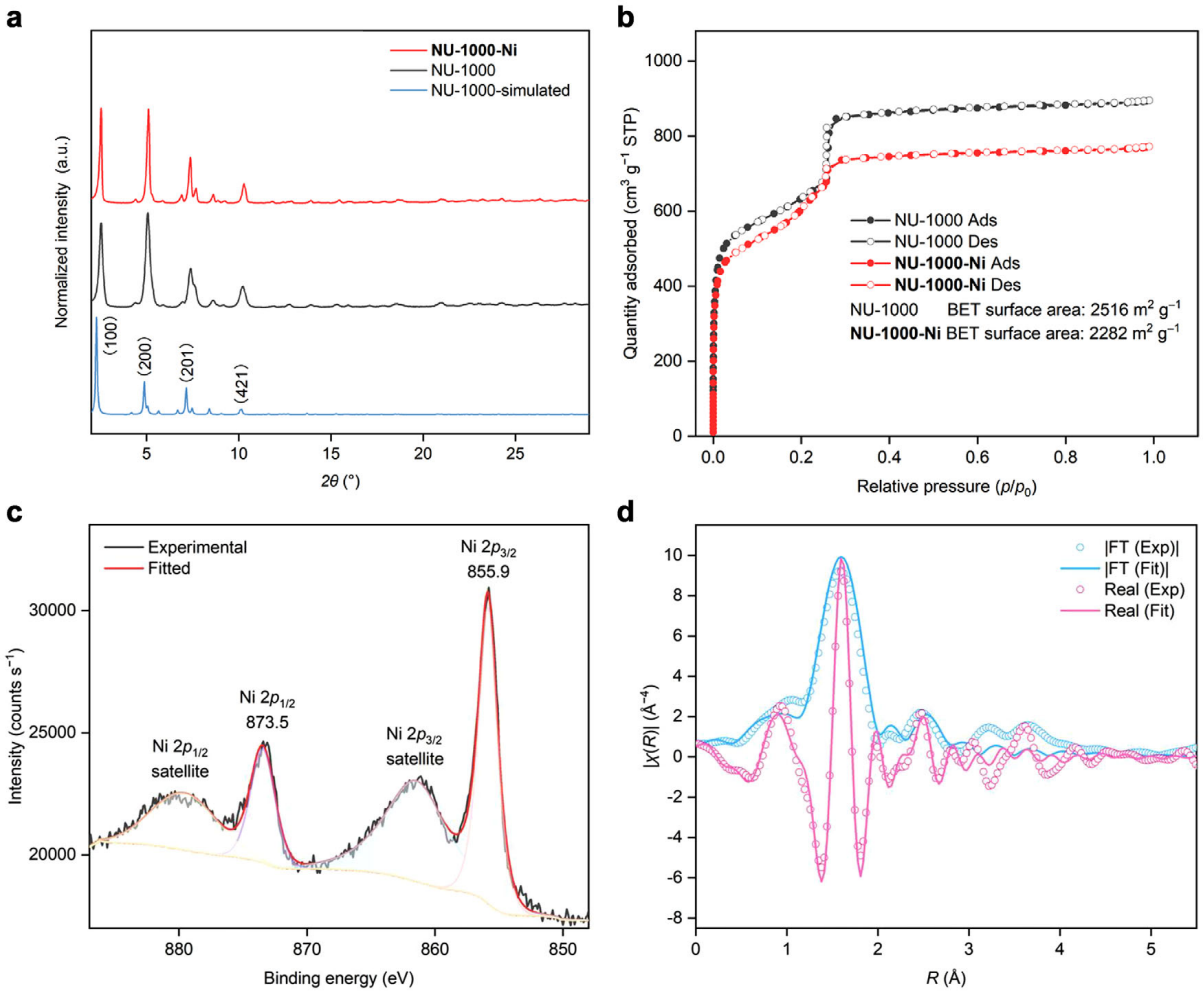
a) Comparing PXRD patterns of **NU‐1000‐Ni** and NU‐1000 with simulations of NU‐1000. b) N_2_ adsorption/desorption isotherms collected at 77 K. c) Ni 2*p* XPS spectrum showing the +2 oxidation state of the Ni centers in **NU‐1000‐Ni**. d) EXAFS spectra and fitting data of Ni K‐edge absorption. *χ* is the fine structure function. *R* is the interatomic distance from Ni.

Powder X‐ray diffraction (PXRD) patterns indicated that the crystallinity of the framework remained well‐preserved after post‐synthetic metalation, exhibiting sharp peaks below 10° corresponding to the (100), (200), (201), and (421) reflection planes^[^ ^47^ ^]^ (**Figure** **3**a). Nitrogen adsorption isotherms at 77 K revealed a slight decrease in the Brunauer–Emmett–Teller (BET) surface area^[^ ^48^ ^]^ from 2516 to 2282 m^2^ g^−1^, further confirming the structural integrity of NU‐1000 during catalyst preparation (Figure 3b). X‐ray photoelectron spectroscopy (XPS) analysis confirmed the presence of only Ni^II^ species in **NU‐1000‐Ni**, with peaks at 873.5 and 855.9 eV assigned to Ni 2*p*_1/2_ and Ni 2*p*_3/2_,^[^ ^49^ ^]^ respectively (Figure 3c). To investigate the coordination environment of nickel centers following solvent coordination to **NU‐1000‐Ni**, extended X‐ray absorption fine structure (EXAFS) was employed to elucidate the binding modes of **NU‐1000‐Ni‐DMF** (Figure 3d). Data analysis revealed the presence of two Ni–Br and four Ni–N/O bonds at 2.838(31) and 2.040(11) Å, respectively (Table S1, Supporting Information). As the mono‐bpy‐ligated nickel(II) complex featured two Ni─Br and two Ni─N bonds, the additional two Ni─N/O bonds were attributed to Ni─O interactions between the nickel center and the two coordinating solvent molecules. In the case of the homogeneous counterpart, heating **Ni‐1** in DMSO‐*d*_6_ at 50 °C for 15 min broke its symmetrical geometry, as evidenced by the ^1^H NMR spectrum (Figure S2, Supporting Information),^[^ ^50^, ^51^ ^]^ indicating the successful coordination of DMSO‐*d*_6_.

After evaluating various reaction parameters (Table S2, Supporting Information), we established a procedure that facilitated the targeted photoinduced sulfonylation of 4‐bromobenzonitrile using catalytic amounts of **NU‐1000‐Ni‐DMA** (**Table** **1**, entry 1; 74% yield). Control experiments underscored the significance of both the catalyst and light in the C─S coupling reaction (entries 2 and 3). While weakly coordinating solvents such as DCM, THF, and EA proved ineffective in the heterogeneous photocatalysis (entry 4), performing the cross‐coupling with **NU‐1000‐Ni‐DMF** in DMF afforded product 1 in a comparable yield to the standard conditions (entry 5). Importantly, the use of **NU‐1000‐Ni**, the MOF‐supported nickel catalyst without pre‐treatment in coordinating solvents, dramatically reduced the product yield to 46% (entry 6), highlighting that DMA coordination to the nickel center enhanced the catalytic activity in the sulfonylation reaction. As expected, the bpy‐based homogeneous nickel catalysts lacking photoactive functionalities exhibited significantly lower reactivity (entries 7–10). These findings confirm the essential role of PCs in homogeneous catalytic systems when sophisticated ligand design is not employed.^[^ ^52^, ^53^, ^54^ ^]^ Interestingly, although NU‐1000 alone failed to catalyze C─S bond formation, the framework itself consumed starting material under light irradiation (entry 11). This observation suggests the MOF possesses inherent photoactivity that could potentially replace PCs in the reaction system.^[^ ^36^, ^55^ ^]^ Furthermore, in comparison to the sulfonylation reactions using **Ni‐1** and NU‐1000 separately (entries 7 and 11), the joint application of **Ni‐1** and NU‐1000 resulted in an improved yield of 36% (entry 12). In contrast, employing **Ni‐2** without a benzoate linkage did not boost reactivity when combined with NU‐1000 (entry 13). These comparative analyses underline the significance of integrating nickel species into framework supports to achieve synergistic effects^[^ ^26^, ^56^, ^57^ ^]^ that promote the photoinduced C─S cross‐coupling. Despite the ineffectiveness of heterogeneous nickel catalysts derived from [2,2′‐bipyridine]‐5,5′‐dicarboxylic acid (entries 14 and 15),^[^ ^58^, ^59^ ^]^ the framework support featuring extended organic linkers yielded a UiO‐type MOF catalyst^[^ ^60^ ^]^ demonstrating superior reactivity to its homogeneous counterpart (entry 16). It is worth noting that we consistently observed slightly increased yields when employing recycled MOF‐supported photocatalysts in subsequent runs (entries 17 and 18), likely attributable to enhanced interaction kinetics between the immobilized nickel centers and coupling partners. Significantly, the heterogeneous nickel‐catalyzed sulfonylation has excellent moisture compatibility, with added water showing no detrimental effect on the reaction (entry 19). To our delight, aryl iodides also serve as effective substrates in the newly developed nickel photocatalysis (entry 20).

**Table 1:** Effects of reaction parameters on the photoinduced nickel‐catalyzed sulfonylation of aryl halides.

Entry	Change from “standard conditions”	Yield^a)^ [%]	Conversion^a)^ [%]
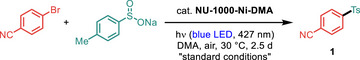			
1	None	74	>95
2	No catalyst	13	15
3	No light	<5	<5
4	DCM, THF, or EA as solvent	<5	<5
5	**NU‐1000‐Ni‐DMF** as catalyst in DMF	70	>95
6	**NU‐1000‐Ni** as catalyst	46	74
7	**Ni‐1** as catalyst	19	25
8	**Ni‐2** as catalyst	23	27
9	**Ni‐3** as catalyst	13	13
10	**Ni‐4** as catalyst	12	14
11	NU‐1000 as catalyst	5	40
12	**Ni‐1** and NU‐1000 as catalyst	36	71
13	**Ni‐2** and NU‐1000 as catalyst	12	65
14	**MOF‐253‐bpy‐Ni‐DMA** as catalyst	<5	<5
15	**UiO‐67‐bpy‐Ni‐DMA** as catalyst	<5	<5
16	**UiO‐69‐bpy‐Ni‐DMA** as catalyst	38	55
17	**NU‐1000‐Ni‐DMA‐1** ^b)^ as catalyst	80	>95
18	**UiO‐69‐bpy‐Ni‐DMA‐1** ^b)^ as catalyst	50	58
19	H_2_O (6 equiv.)	74	>95
20	Ar–I, instead of Ar–Br	85	>95
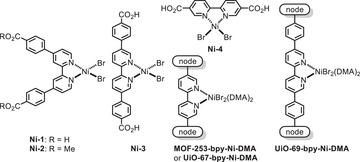			

Standard conditions: 4‐bromobenzonitrile (0.2 mmol, 1.0 equiv.), sodium *p*‐toluenesulfinate (3.0 equiv.), and **NU‐1000‐Ni‐DMA** (20 mg, 5.0 mol% Ni) in DMA (2.0 mL) under air with blue‐LED light irradiation (427 nm) for 2.5 d.

a) Yield of the product and conversion of the aryl bromide were determined by ^1^H NMR of the crude product using 1,2‐dibromoethane as an internal standard. Ts, *p*‐toluenesulfonyl; **MOF‐253‐bpy‐Ni‐DMA**, an aluminum‐based metal−organic framework containing nickel‐functionalized 2,2′‐bipyridine dicarboxylate organic linkers^[^ ^57^ ^]^ upon DMA treatment; **UiO‐67‐bpy‐Ni‐DMA**, a metal−organic framework containing [Zr_6_] inorganic nodes and nickel‐functionalized 2,2′‐bipyridine dicarboxylate organic linkers58 upon DMA treatment; **UiO‐69‐bpy‐Ni‐DMA**, a metal−organic framework containing [Zr_6_] inorganic nodes and nickel‐functionalized 2,2′‐bipyridine dibenzoate organic linkers^[^ ^59^ ^]^ upon DMA treatment.

b) Recycled catalysts after the first run.

**Figure 4:**
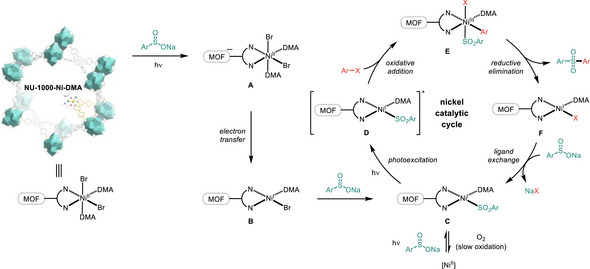
Proposed mechanism for sulfonylation of aryl halides in heterogeneous nickel photocatalysis.

Solid‐state absorption spectra revealed that both pristine and recycled heterogeneous nickel catalysts displayed enhanced absorption relative to **Ni‐1** at the wavelength of ≈427 nm (Figure S9, Supporting Information). In addition, the coupling process was completely inhibited when light irradiation was removed during the reaction (Figure S2, Supporting Information). Based on comprehensive control experiments and mechanistic investigations, we propose the following catalytic cycle for the sulfonylation reaction (**Figure** **4**). Under light irradiation, single‐electron reduction by sodium sulfinates generates a radical anion on the framework matrix (**A**),^[^ ^36^, ^61^ ^]^ which subsequently transfers an electron to the immobilized nickel(II) complex to yield solvent‐bound nickel(I) species **B**.^[^ ^6^, ^62^ ^]^ Following transmetalation, the MOF‐supported Ni─SO2Ar species undergoes oxidative addition with an aryl halide after photoexcitation to form intermediate **E**, likely serving as the rate‐limiting step.^[^ ^32^ ^]^ The reductive elimination of this nickel(III) species delivers the sulfone product and supported nickel(I) **F**, which completes the catalytic cycle by transmetalating with a sulfinate. The limited oxygen diffusion within the MOF pores renders aerobic oxidation of the supported nickel(I) intermediate **C** more difficult than in homogeneous systems. This accounts for the remarkable air tolerance observed in the heterogeneous nickel‐catalyzed sulfonylation.

**Figure 5:**
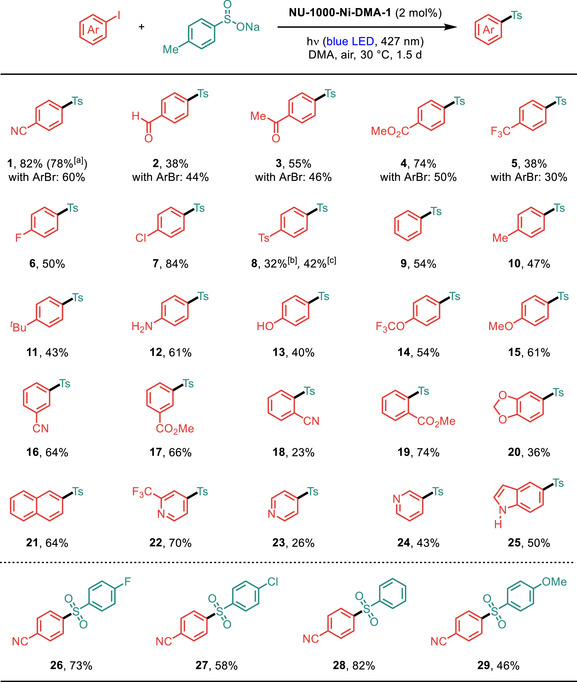
Reaction conditions: aryl halide (0.2 mmol, 1.0 equiv.), sodium aryl sulfinate (3.0 equiv.), and **NU‐1000‐Ni‐DMA‐1** (20 mg, 2.0 mol% Ni) in DMA (2.0 mL) under air with blue‐LED light irradiation (427 nm, 3 × 40 W) for 1.5 or 2.5 d. For each entry number (in bold), data are reported as isolated yields. a) Gram‐scale synthesis with 0.5 mol% of **NU‐1000‐Ni‐DMA‐1**. b) 1,4‐Diiodobenzene as the substrate. c) 1‐Bromo‐4‐iodobenzene as the substrate; sodium *p*‐toluenesulfinate (5.0 equiv.) was used. Ts, *p*‐toluenesulfonyl; *^t^* Bu, *tert*‐butyl.

Having established the optimal conditions, we chose sodium *p*‐toluenesulfinate as the nucleophile to explore the scope of aryl halides (**Figure** **5**). The reaction demonstrated broad functional group compatibility in aryl iodides, accommodating substituents with diverse electronic properties, whereas aryl bromides required electron‐withdrawing groups to facilitate efficient oxidative addition (Figure 5, products **1**–**15**). Aniline and phenol derivatives were well tolerated in the C─S coupling (Figure 5, products **12** and **13**). For 1,4‐disulfonylation, sequential coupling reactions using 1‐bromo‐4‐iodobenzene proved superior to 1,4‐diiodobenzene as the substrate (Figure 5, product **8**). We further examined substrates bearing *meta*‐ and *ortho*‐substituents (Figure 5, products **16**–**19**). Notably, *ortho*‐carbonyl groups on the aryl iodide substrates enhanced reactivity, facilitating oxidative addition through pre‐coordination with the nickel center (Figure 5, product **19**). In addition to tolerating di‐substituted and polycyclic aromatic rings (Figure 5, products **20** and **21**), the electrophile scope includes pyridine and indole derivatives (Figure 5, products **22**–**25**). To evaluate the sulfinate scope, 4‐iodobenzonitrile was employed as the model electrophile. A range of sodium sulfinates with either an electron‐donating or electron‐withdrawing substituent on the aryl group furnished the desired products in good yields (Figure 5, products **26**–**29**). Remarkably, the gram‐scale synthesis of sulfone **1** proceeded efficiently with just 0.5 mol% catalyst loading, achieving a high isolated yield of 78%.

**Figure 6:**
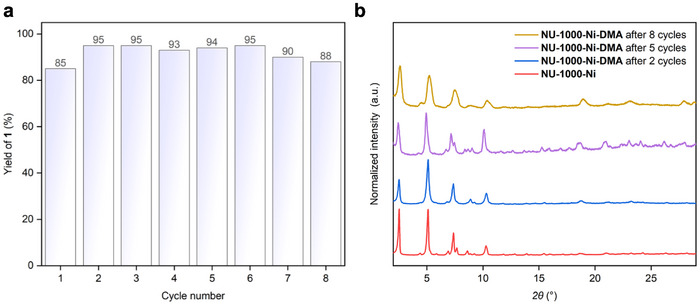
a) Recycling experiments for sulfonylation of 4‐iodobenzonitrile with sodium *p*‐toluenesulfinate using an equal amount of **NU‐1000‐Ni**. Yield was determined by ^1^H NMR of the crude product using 1,2‐dibromoethane as an internal standard. b) PXRD patterns of the original (red line) and recycled heterogeneous nickel photocatalyst after two (blue line), five (purple line), and eight cycles (yellow line).

Encouragingly, **NU‐1000‐Ni‐DMA** exhibited excellent catalytic performance in the photoinduced sulfonylation of aryl halides while maintaining remarkable recyclability, consistently delivering product 1 in good yields over eight consecutive cycles (**Figure** **6**a). The PXRD patterns confirmed retention of NU‐1000′s crystallinity throughout the catalytic runs (Figure 6b). The inductively coupled plasma mass spectrometry analysis of **NU‐1000‐Ni‐DMA** revealed substantial nickel leaching during the initial catalytic cycle, exhibiting a 60% decrease in nickel content. However, subsequent cycles showed much greater stability, with merely 16% additional nickel loss observed after eight cycles. This initial metal leaching likely originates from surface‐bound **Ni‐1** species rather than the effectively immobilized nickel complexes within the MOF pores.

## 3 Conclusion

In summary, we have developed a heterogeneous photocatalytic system based on NU‐1000‐supported nickel catalysts for the sulfonylation of aryl halides under visible light irradiation. The synergistic interaction between the photoactive MOF linkers and anchored nickel centers allows for efficient C─S bond formation without requiring the use of external photocatalysts or elaborate ligand design. Notably, this system offers excellent scalability, catalyst recyclability, and the unique capability to facilitate photoinduced nickel‐catalyzed coupling reactions in ambient air. Moreover, the exclusive generation of mono‐bpy‐ligated nickel complexes on the MOF framework presents a well‐defined platform for investigating solvent coordination effects on reactivity, a challenging aspect to explore in homogeneous catalysis.

## Conflict of Interest

The authors declare no conflict of interest.

## Author Contributions

G.W. synthesized the framework‐based nickel catalysts, conducted most of the experiments for characterization, and optimized the photocatalytic reaction conditions. T.L. simulated the PXRD patterns. C.K.T.W. and T.W.B.L. simulated the EXAFS spectra. J.H. directed the project and wrote the manuscript with the contributions from G.W.

## Supporting information



Supporting Information

## Data Availability

The data that support the findings of this study are available from the corresponding author upon reasonable request.
