# Supplementary material for: Air‐Insensitive Sulfonylation Enabled by a MOF‐Supported Nickel Photocatalyst
Source: Small. 2025 Sep 27;21(46):e08991. doi: 10.1002/smll.202508991 (PMC12632432; doi:10.1002/smll.202508991)
Supplement: Supplementary file 1 — Supporting Information [file SMLL-21-e08991-s001.pdf]

NANO · MICRO small

Supporting Information

for *Small*, DOI 10.1002/smll.202508991

Air-Insensitive Sulfonylation Enabled by a MOF-Supported Nickel Photocatalyst

*Guannan Wang, Tianyu Liu, Ching Kit Tommy Wun, Tsz Woon Benedict Lo and Jian He**

Supporting Information

Air-Insensitive Sulfonylation Enabled by a MOF-Supported Nickel Photocatalyst

Guannan Wang[1], Tianyu Liu[1], Ching Kit Tommy Wun[4], Tsz Woon Benedict Lo[4], and Jian He[1,2,3]*

[1]Department of Chemistry, The University of Hong Kong, Pokfulam Road, Hong Kong 999077, China.

[2]State Key Laboratory of Synthetic Chemistry, The University of Hong Kong, Hong Kong 999077, China.

[3]Materials Innovation Institute for Life Sciences and Energy (MILES), HKU-SIRI, Shenzhen 518063, China.

[4]State Key Laboratory of Chemical Biology and Drug Discovery, Department of Applied Biology and Chemical Technology, The Hong Kong Polytechnic University, Hong Kong 999077, China.

*Email: jianhe@hku.hk

Table of contents

1. **Testing reported systems under air** .... S−2

2. **Preparations for organic linkers and nickel complexes** .... S−3

3. **Preparations for heterogeneous catalysts** .... S−12

4. **Photoinduced sulfonylation of aryl halides catalyzed by heterogeneous catalysts** .... S−23

5. **References** .... S−59

6. **NMR spectra** .... S−60

1. Testing reported systems under air

The reported systems of blue-light-induced sulfonylation of aryl halides must require noble-gas environments.[1-3]

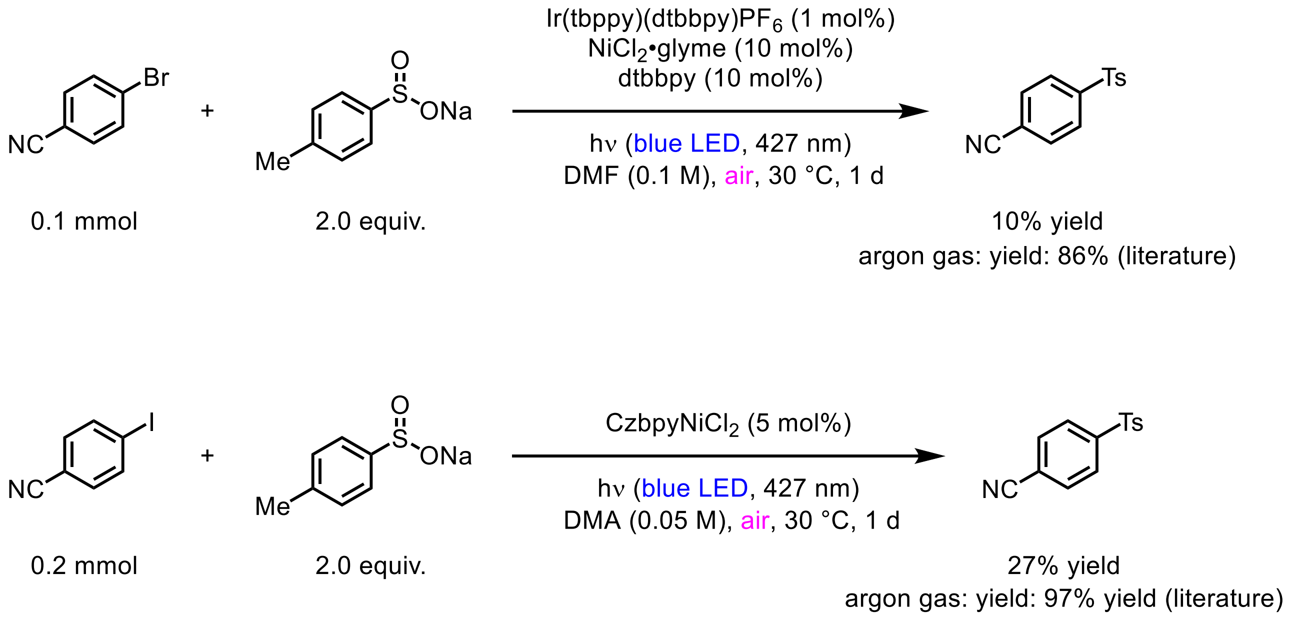

Figure S1. Testing N_2-demanding systems under air.

2. Preparations for organic linkers and nickel complexes

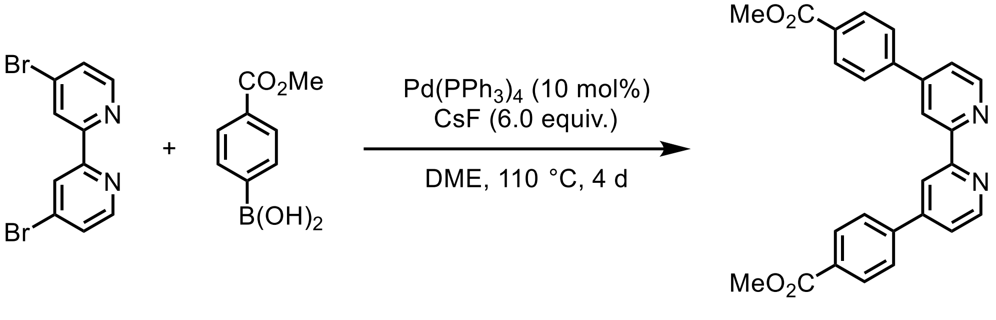

Dimethyl 4,4'-([2,2'-bipyridine]-4,4'-diyl)dibenzoate (**L1**). To a mixture of 4,4'-dibromo-2,2'-bipyridine (3.0 mmol, 0.942 g), (4-(methoxycarbonyl)phenyl)boronic acid (4.0 equiv., 12.0 mmol, 2.16 g), $Pd(PPh_3)_4$ (10 mol%, 0.3 mmol, 347 mg) and CsF (6.0 equiv., 18.0 mmol, 2.73 g) was added dimethoxyethane (DME) (30 mL) under N_2 atmosphere. The mixture was heated to 110 °C and stirred for 4 days (if bulky lump was formed during the reaction, the lump was sonicated into powder to release the wrapped substrates). After the completion of the reaction, ethyl acetate (EA) (90 mL) was poured into the reaction system, and the mixture was sonicated for 15 min. The mixture was then filtered to collect the solids, followed by washing with EA (90 mL) to remove most soluble impurities. The crude product was subjected to Soxhlet extraction with chloroform for 4 days to remove insoluble salts, and the extraction was concentrated *in vacuo*. The afforded precipitate was sequentially rinsed with acetone (10 mL), dichloromethane (DCM) (10 mL), and diethyl ether (30 mL) to remove the residue of soluble impurities. The resulting precipitate was dried *in vacuo* to give **L1** as white solids (1.00 g, 79% yield).

^{1}H NMR (400 MHz, $CDCl_3$) δ 8.82 – 8.74 (m, 4H), 8.18 (d, J = 8.4 Hz, 4H), 7.86 (d, J = 8.4 Hz, 4H), 7.60 (dd, J = 5.2, 2.0 Hz, 2H), 3.97 (s, 6H).

^{13}C NMR (125 MHz, $CDCl_3$) δ 166.7, 156.6, 149.8, 148.3, 142.6, 130.6, 130.3, 127.2, 121.9, 119.3, 52.3.

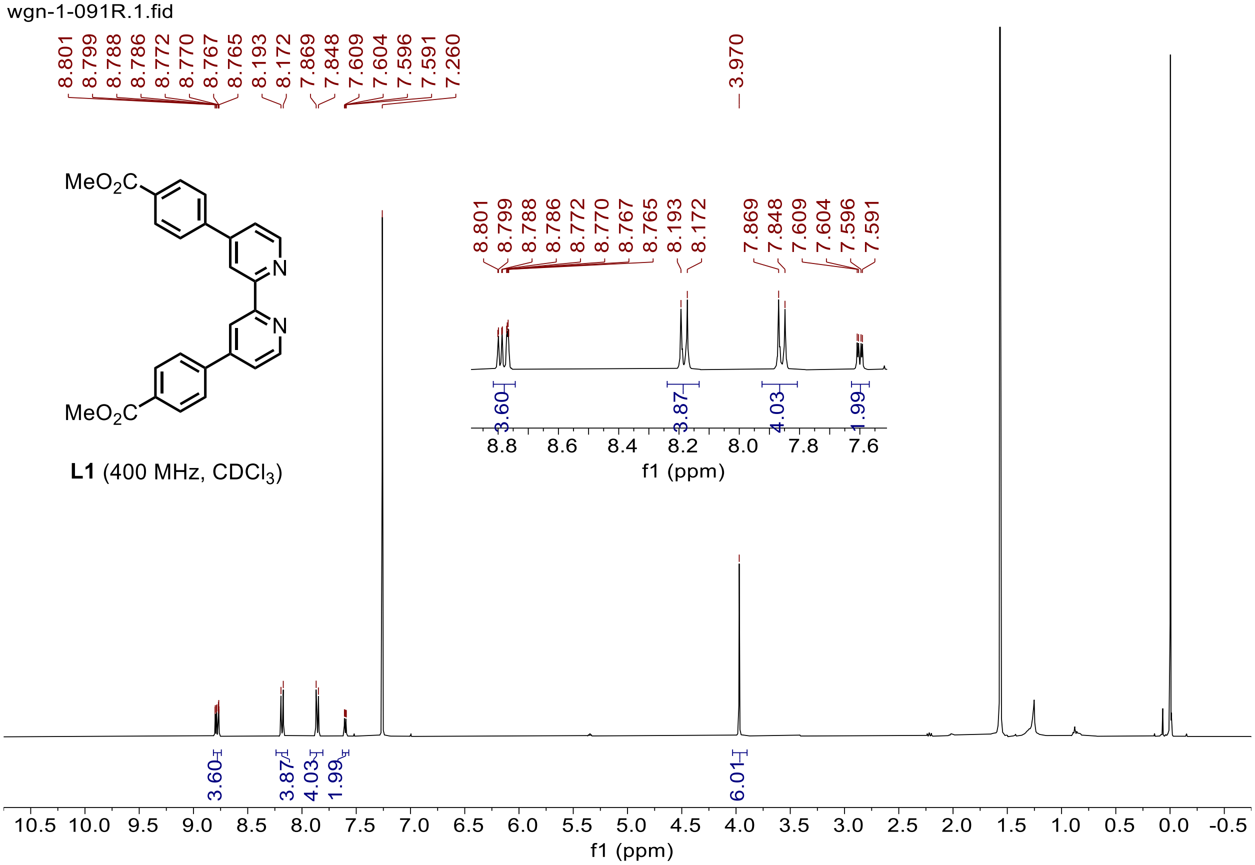
wgn-1-091R.1.fid
8.801 8.799 8.788 8.786 8.772 8.770 8.767 8.765 8.193 8.172 7.869 7.848 7.609 7.604 7.596 7.591 7.260
3.970
MeO_2C
N
N
MeO_2C
L1 (400 MHz, $CDCl_3$)
8.801 8.799 8.788 8.786 8.772 8.770 8.767 8.765 8.193 8.172 7.869 7.848 7.609 7.604 7.596 7.591
3.60 3.87 4.03 1.99
8.8 8.6 8.4 8.2 8.0 7.8 7.6
f1 (ppm)
3.60 3.87 4.03 1.99 6.01
10.5 10.0 9.5 9.0 8.5 8.0 7.5 7.0 6.5 6.0 5.5 5.0 4.5 4.0 3.5 3.0 2.5 2.0 1.5 1.0 0.5 0.0 -0.5
f1 (ppm)

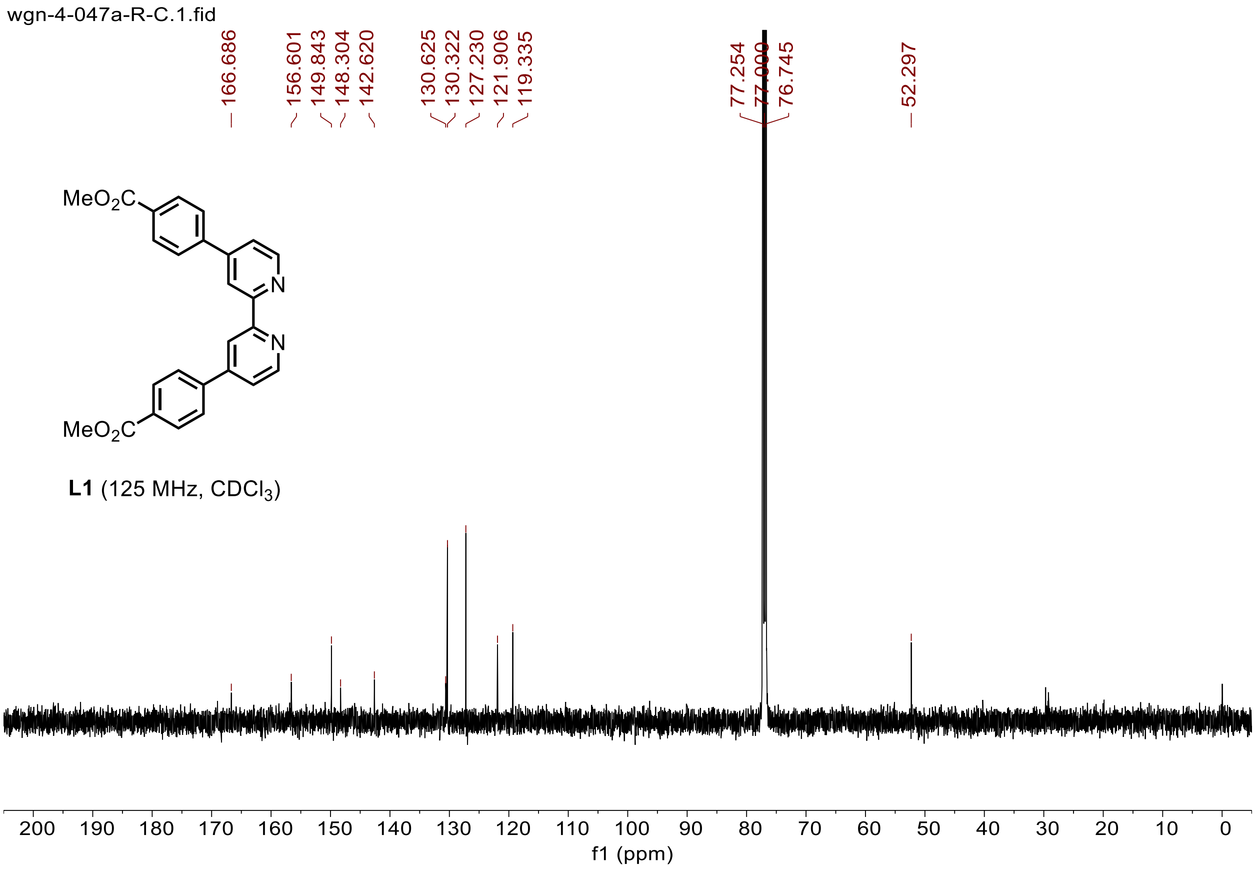
wgn-4-047a-R-C.1.fid
166.686
156.601
149.843
148.304
142.620
130.625
130.322
127.230
121.906
119.335
77.254
77.000
76.745
52.297
MeO2C
N
N
MeO2C
L1 (125 MHz, CDCl3)
200 190 180 170 160 150 140 130 120 110 100 90 80 70 60 50 40 30 20 10 0
f1 (ppm)

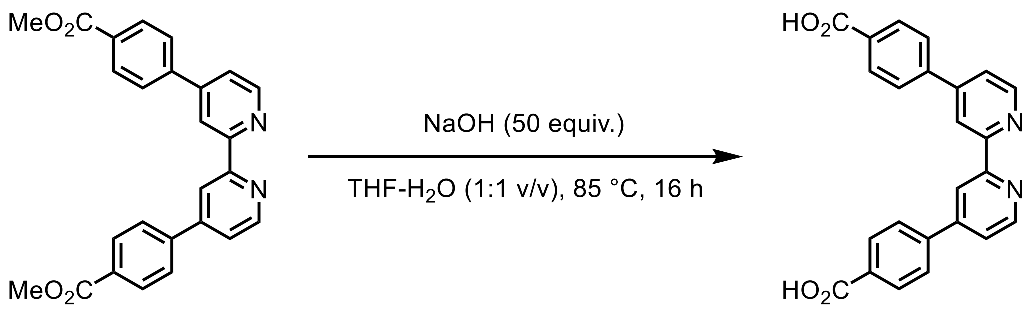

L1 (3.0 mmol) **L2**, quantitative yield

4,4'-([2,2'-Bipyridine]-4,4'-diyl)dibenzoic acid (**L2**). To a suspension of **L1** (3.0 mmol, 1.27 g) in THF (60 mL) was added NaOH (50.0 equiv., 150.0 mmol, 12.00 g) in H_2O (60 mL) under N_2 atmosphere. The mixture was heated to 85 °C and stirred for 16 h. The reaction system was cooled to ambient temperature after its completion, and THF was removed under reduced pressure. The afforded suspension was acidified to pH = 4 – 5 with concentrated HCl and filtered to collect the formed solids. The solids were then washed with deionized water to remove inorganic salts. The purified product was dried in an oven at 60 °C for 24 h to give **L2** (1.2 g, quantitative yield) as pinkish solids. Due to the bad solubility of **L2** (almost no corresponding signal in ^{1}H NMR was observed with DMSO-d_6), it was transformed into its lithium salt (**L2'**) for characterization according to the reference.[4]

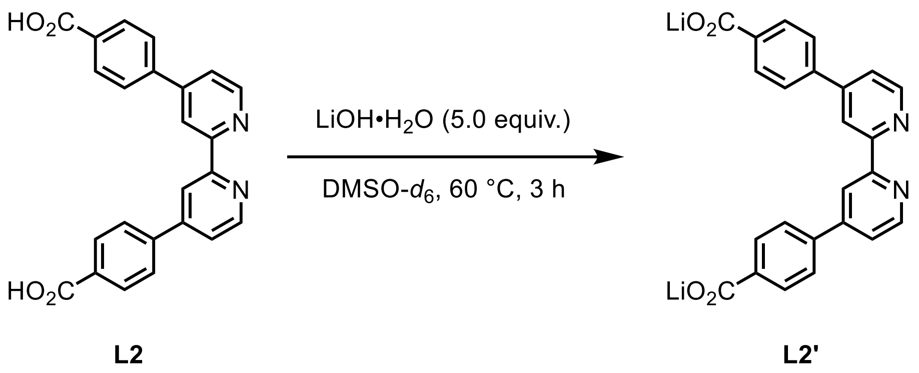

Lithium 4,4'-([2,2'-bipyridine]-4,4'-diyl)dibenzoate (**L2'**). To a mixture of **L2** (0.02 mmol, 7.9 mg) and LiOH•H_2O (5.0 equiv., 0.10 mmol, 4.0 mg) was added DMSO-d_6 (0.7 mL) under N_2 atmosphere. The reaction system was heated to 60 °C and stirred for 3 h. After the completion of the reaction, the reaction system was filtered, and the filtrate was directly used for ^{1}H NMR.

^{1}H NMR (400 MHz, DMSO-d_6) δ 8.79 – 8.78 (m, 2H), 8.73 (s, 2H), 8.03 – 8.01 (m, 4H), 7.82 – 7.79 (m, 6H).

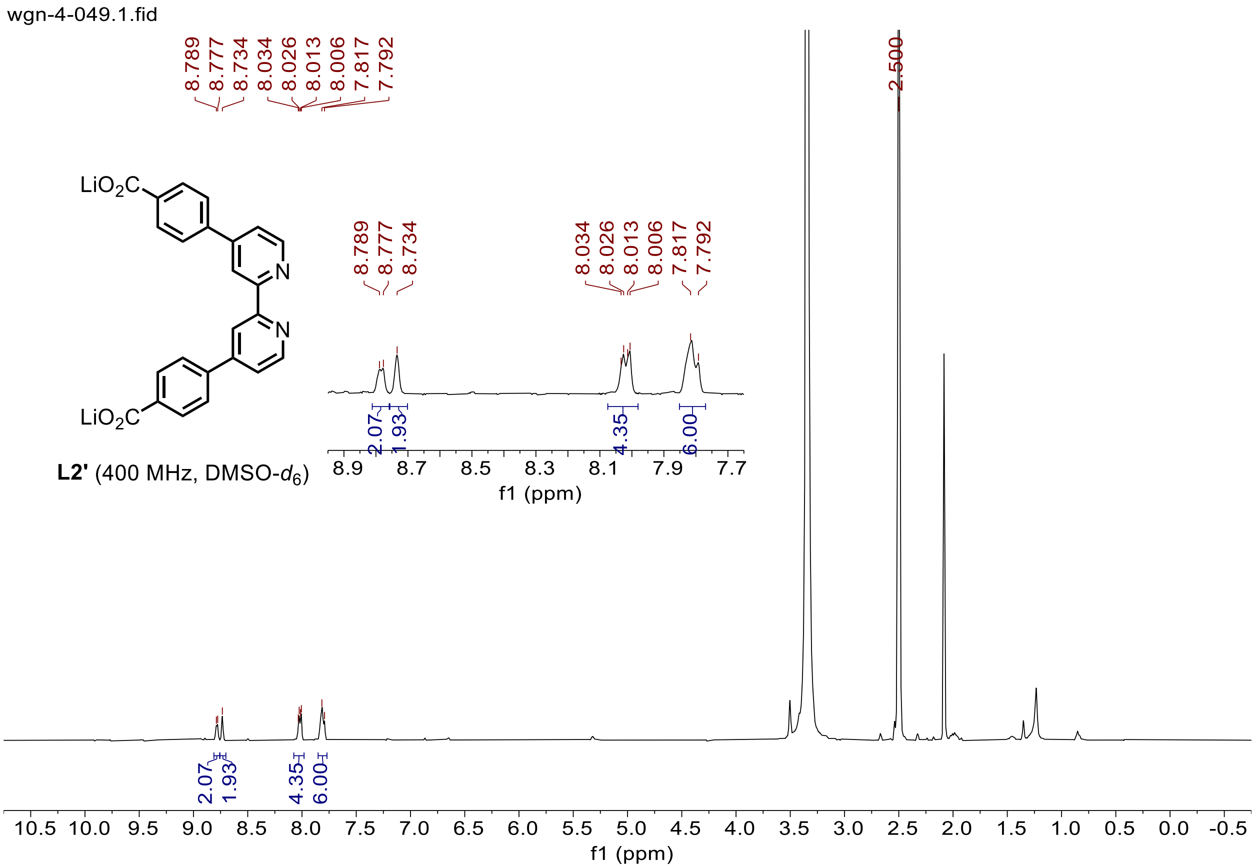
wgn-4-049.1.fid
8.789
8.777
8.734
8.034
8.026
8.013
8.006
7.817
7.792
2.500
LiO2C
N
N
LiO2C
L2' (400 MHz, DMSO-d6)
2.07
1.93
4.35
6.00
f1 (ppm)

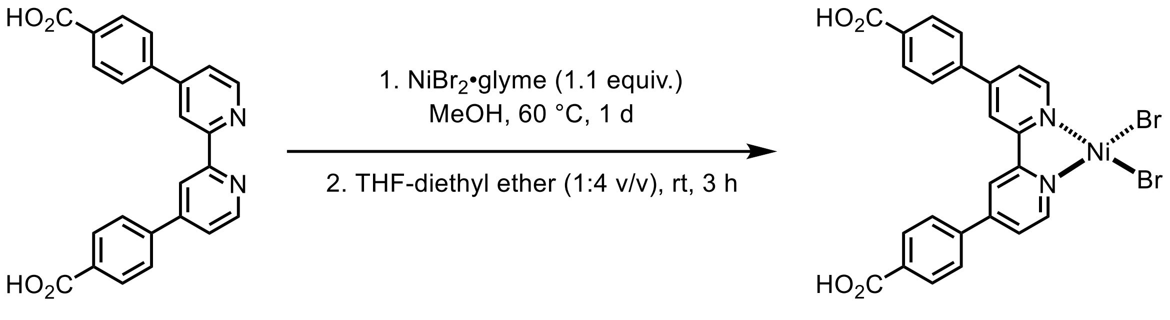

L2 (3.0 mmol) **Ni-1**, 76% yield

Ni-1 was synthesized according to previously reported procedures.[5] To fine-ground **L2** (3.0 mmol, 1.17 g) was added the solution of $NiBr_2$•glyme (1.1 equiv., 3.3 mmol, 1.02 g) in MeOH (10 mL) under N_2 atmosphere. The reaction was heated to 60 °C and stirred for 1 day. After the completion of the reaction, pale green solids were formed. The solids were collected by filtration and then stirred in THF-diethyl ether (1:4 v/v, 10 mL) at ambient temperature for 3 h. The product was filtrated out and dried in an oven at 60 °C for 24 h to give **Ni-1** (1.40 g, 76% yield) as pale green solids.

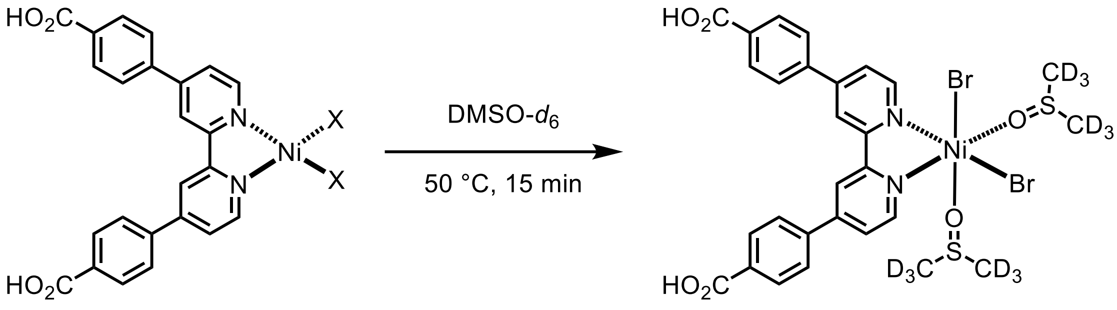

Ni-1 **Ni-1-DMSO-d_6**

Ni-1-DMSO-d_6. **Ni-1** (6 mg) was stirred with DMSO-d_6 (0.5 mL) at 50 °C for 15 min, gradually dissolved. Cooled to ambient temperature, the reaction system was filtered (small amount of **L2** existed as precipitates), and the filtrate was directly sent for ^{1}H NMR.

^{1}H NMR (400 MHz, DMSO-d_6) δ 60.48 – 56.55 (m, 2H), 43.26 – 39.83 (m, 2H), 13.37 (s, 2H), 10.37 – 9.46 (m, 4H), 7,83 – 7.31 (m, 4H).

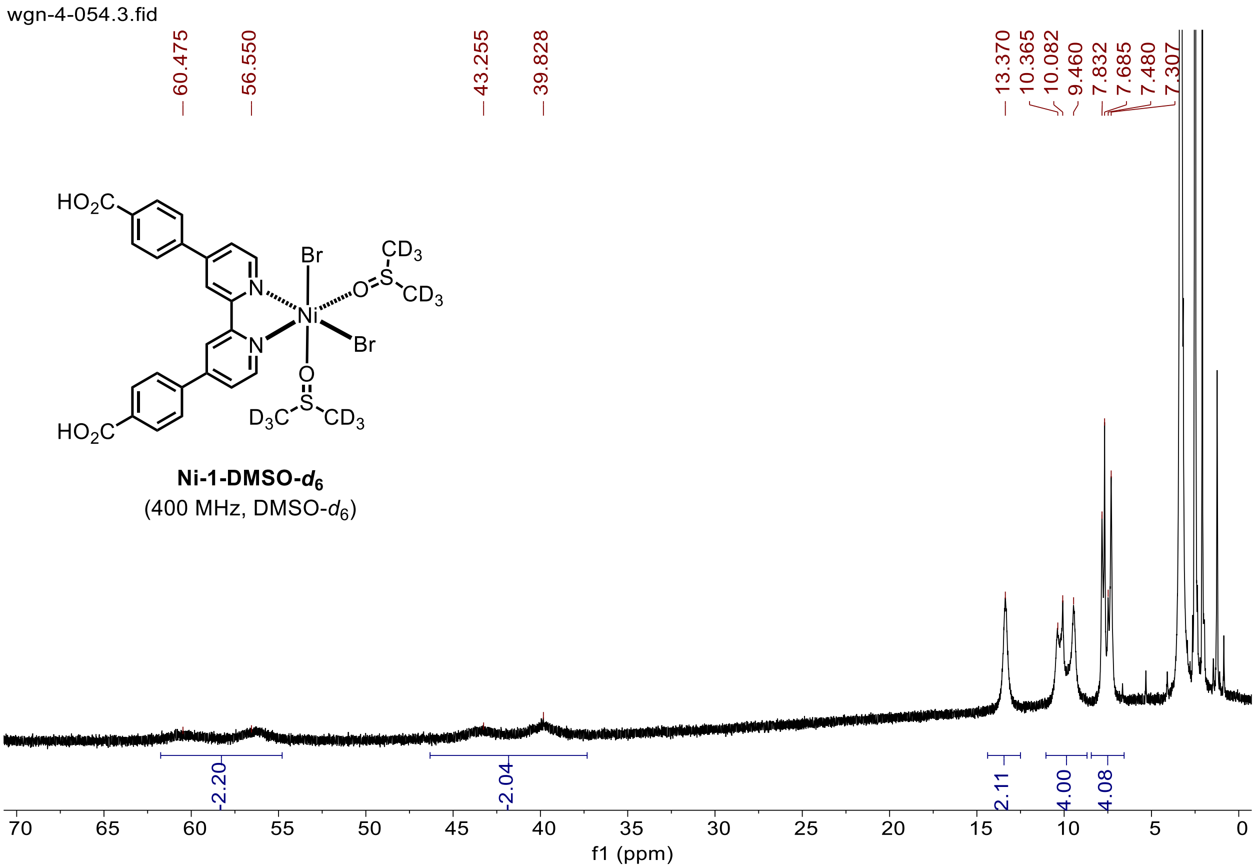

Figure S2. ^{1}H NMR spectrum of **NU-1000-Ni** heated in DMSO-d_6.

3. Preparations for heterogeneous catalysts

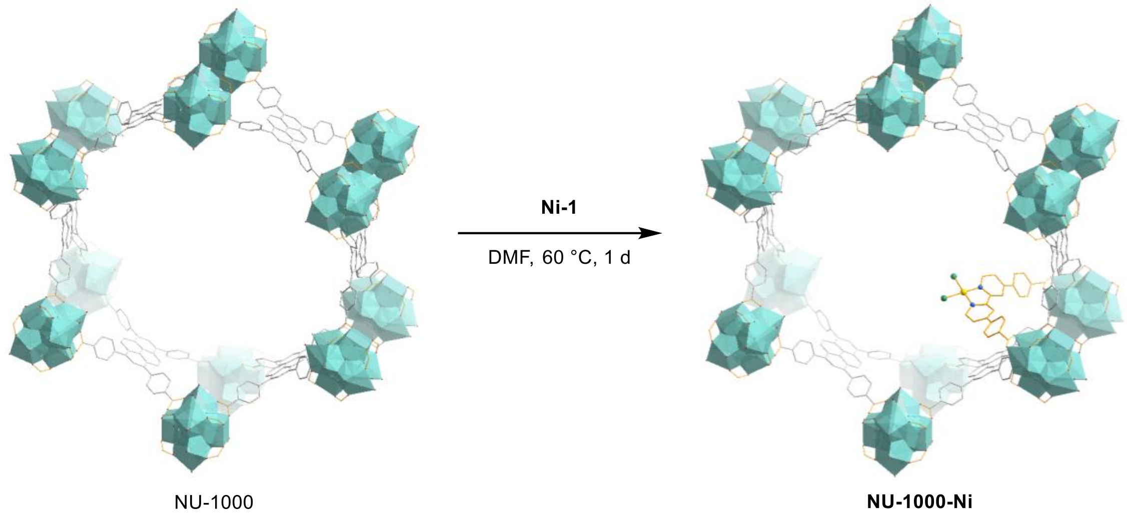

NU-1000-Ni. **Ni-1** (10.0 equiv., 2.5 mmol, 1.54 g) was stirred in DMF (12.5 mL) at 60 °C for 1 h, during which **Ni-**1 gradually dissolved. Meanwhile, NU-1000[6] (prepared on 1-g scale according to literature) was evacuated for 10 min and refilled with N_2, and these processes were repeated 3 times.

The freshly prepared **Ni-1** solution in DMF was filtered to remove the insoluble, and the filtrate was added to N_2-purged NU-1000 (0.25 mmol, 171 mg) under N_2 atmosphere. The mixture was heated to 60 °C for 1 day. After the completion of the reaction, the reaction system was cooled to ambient temperature and the mixture was filtered to collect **NU-1000-Ni** as solids (the filtrate could be recycled). The crude **NU-1000-Ni** was washed with DMF (100 mL), followed by soaking in DMF (100 mL) for 1 day, and the soaking was repeated 3 times to remove residue **Ni-1**. The prepared **NU-1000-Ni** was washed with acetone (100 mL), followed by soaking in acetone (100 mL) for 1 day, and the soaking was repeated 3 times to remove residue DMF. The prepared **NU-1000-Ni** was collected by filtration and dried in an oven at 60 °C for 24 h (180 mg).

Molecular weight of **NU-1000-Ni**: 2176.64 + (614.89 – 18.02 * 2) * 1.5 = 3045.02

Note: molecular weight = **NU-1000** + (**Ni-1** – H_2O * 2) * n; n is the Ni content per NU-1000 unit (per Zr_6), identified via ICP-MS.

For a 0.2-mmol-scale reaction, 20 mg NU-1000-Ni was used as catalyst, which is equal to 5 mol%.

[20 mg * (1 – 8%) * 1.5] / (3045.02 * 0.2 mmol) = 5 mol%

Note: 8% is the weight loss before 200 °C, assigned to solvent residue, identified via TGA.

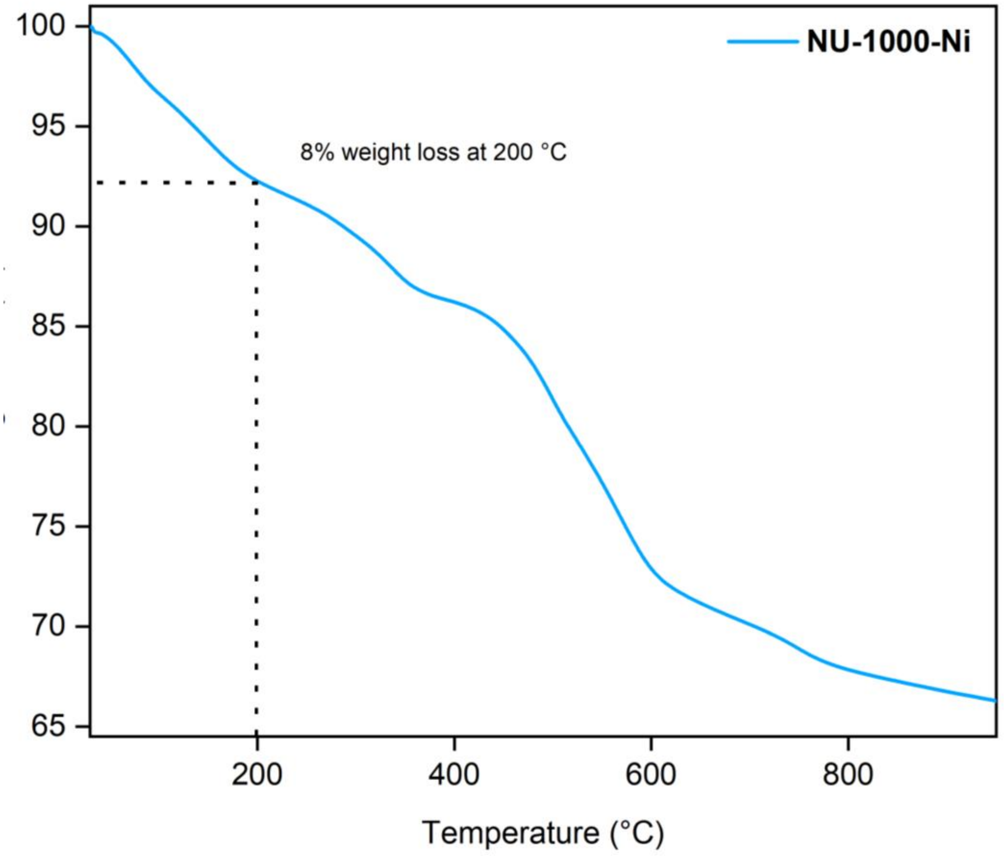

Figure S3. TGA of **NU-1000-Ni**.

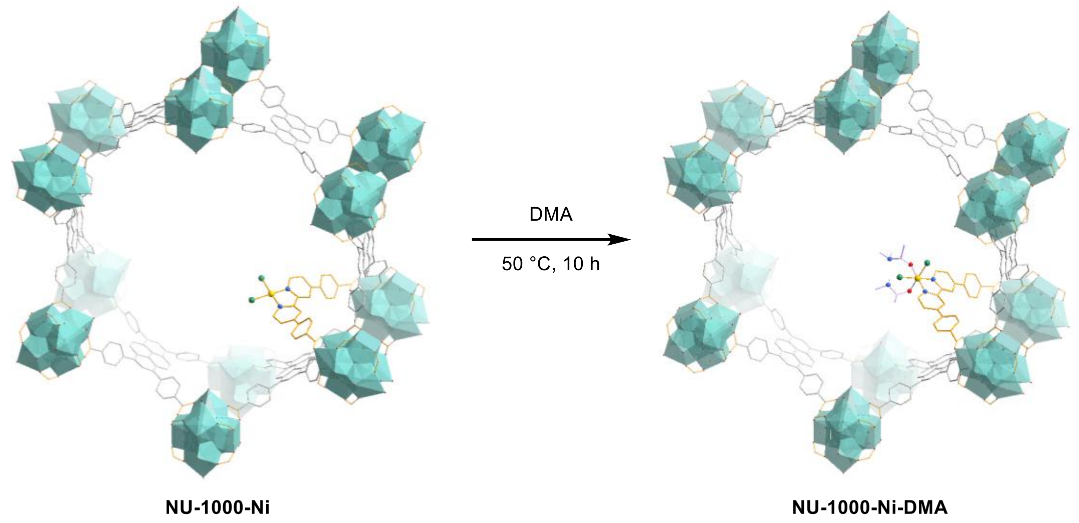

NU-1000-Ni-DMA. **NU-1000-Ni** (20 mg) was evacuated for 10 min and refilled with N_2, and those processes were repeated 3 times. The prepared **NU-1000-Ni** was mixed with DMA (2 mL) under N_2 atmosphere, followed by heated to 50 °C and stirred for 10 h. The **NU-1000-Ni-DMA** suspension in DMA (**NU-1000-Ni-DMA** might lose its coordinated DMA when washed with acetone or diethyl ether) was ready to be directly used for catalysis by mixing with substrates under air. By filtration, **NU-1000-Ni-DMA** was collected as mud, which could be remade into its suspension with general DMA under air.

NU-1000-Ni-DMF was prepared by the same method of preparing **NU-1000-Ni-DMA**, except using DMF instead of DMA.

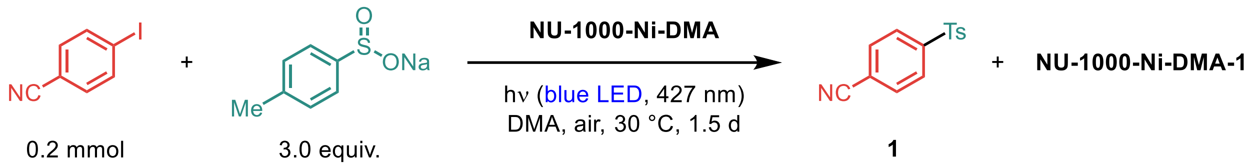

NU-1000-Ni-DMA-1 (first recycle of **NU-1000-Ni-DMA**). To 4-iodobenzonitrile (0.2 mmol, 45.8 mg) and sodium 4-methylbenzenesulfinate (3.0 equiv., 0.6 mmol, 106.9 mg) was added **NU-1000-Ni-DMA** (20 mg, 5 mol% Ni) suspension in DMA (2.0 mL) under air. The reaction mixture was irradiated with blue LED light (Kessil PR-160, 427 nm, 40 W, three lamps) positioned approximately 3 cm away and stirred for 1.5 days. A water-cooling system was used to control the reaction temperature to be at 30 °C. After the completion of the reaction, the reaction system was filtered to recycle the heterogeneous catalyst.

By filtration, **NU-1000-Ni-DMA-1** was collected as mud, which could be remade into its suspension with normal DMA under air.

For characterizations, the **NU-1000-Ni-DMA-1** mud was washed with acetone (20 mL), followed by soaking in acetone (20 mL) for 1 day, and the soaking was repeated 3 times to remove residue DMA. The prepared heterogeneous catalyst was filtrated out, dried in an oven at 60 °C for 24 h, and sent for characterizations.

Molecular weight of **NU-1000-Ni-DMA-1**: 2176.64 + (614.89 – 18.02 * 2) * 0.6 = 2523.95

Note: molecular weight = **NU-1000** + (**Ni-1** – H_2O * 2) * n; n is the Ni content per NU-1000 unit (per Zr_6), identified via ICP-MS.

For a 0.2-mmol-scale reaction, 20 mg NU-1000-Ni was used as catalyst, which is equal to 2 mol%.

[20 mg * (1 – 5%) * 0.6] / (3450.21 * 0.2 mmol) = 2 mol%

Note: 5% is the weight loss before 200 °C, assigned to solvent residue, identified via TGA.

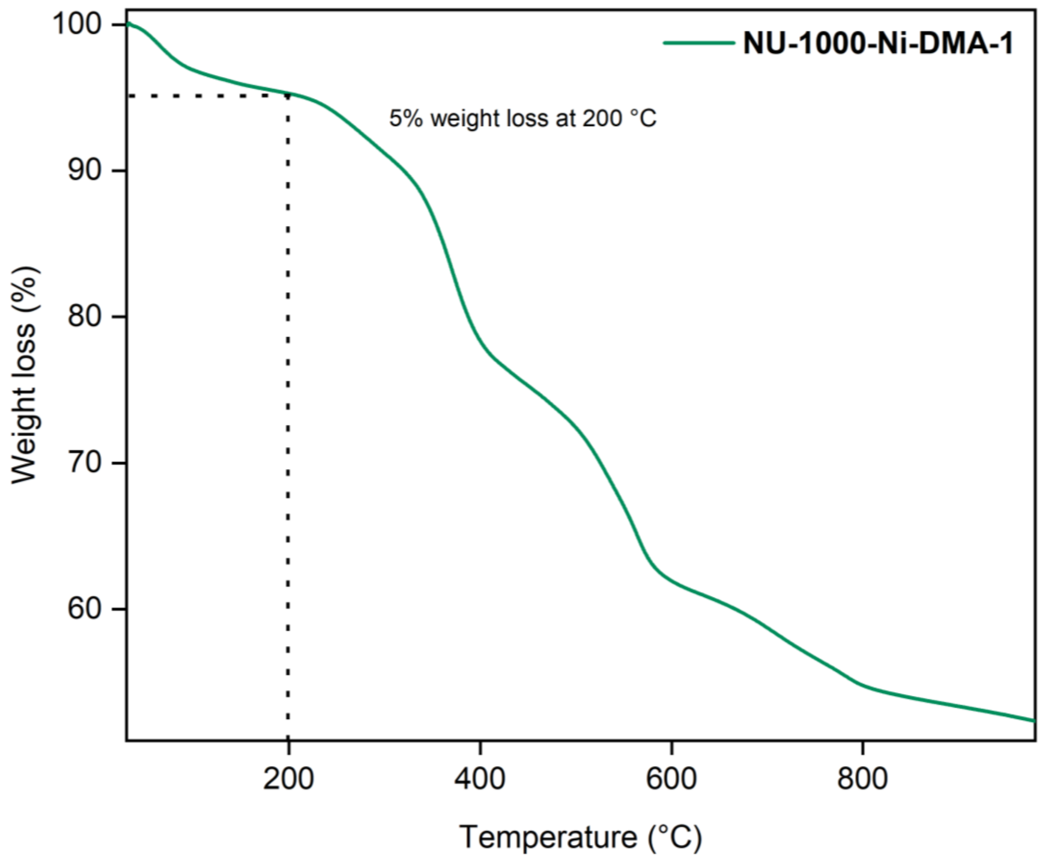

Figure S4. TGA of **NU-1000-Ni-DMA-1**.

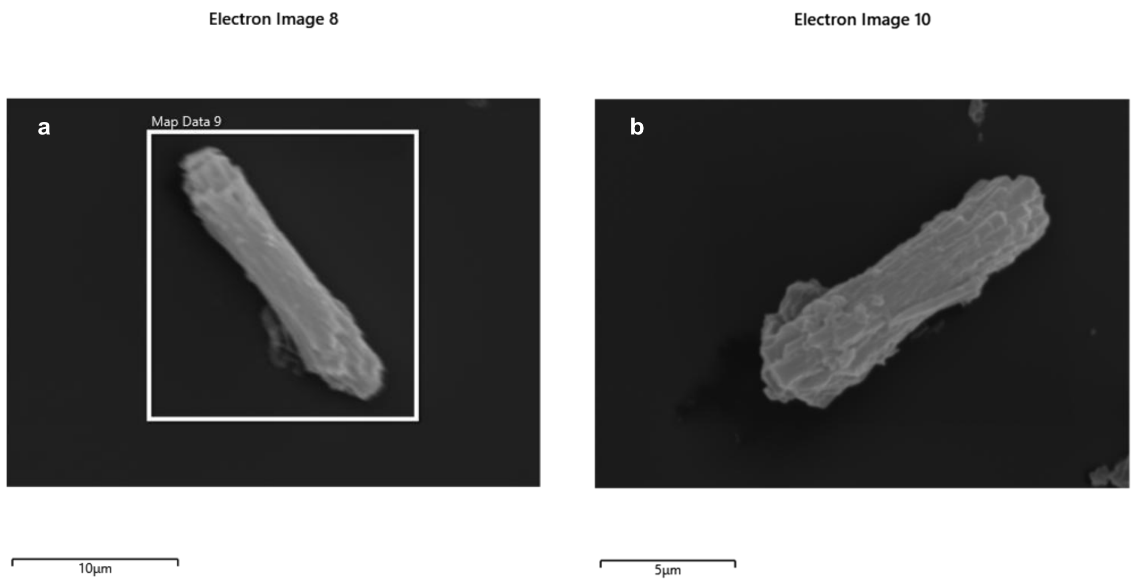

Figure S5. a, SEM image of **NU-1000**. **b**, SEM image of **NU-1000-Ni**.

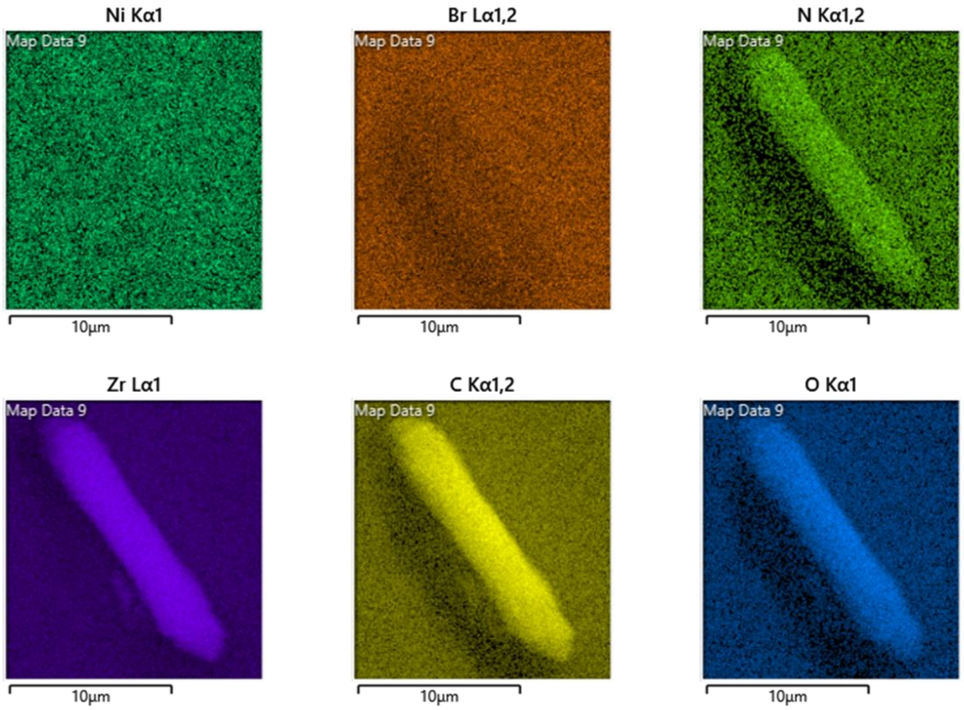

Figure S6. EDS mapping images of **NU-1000**. Ni, Br, N, Zr, C, and O are represented in jade green, bronze, yellowish green, purple, yellow, and blue, respectively.

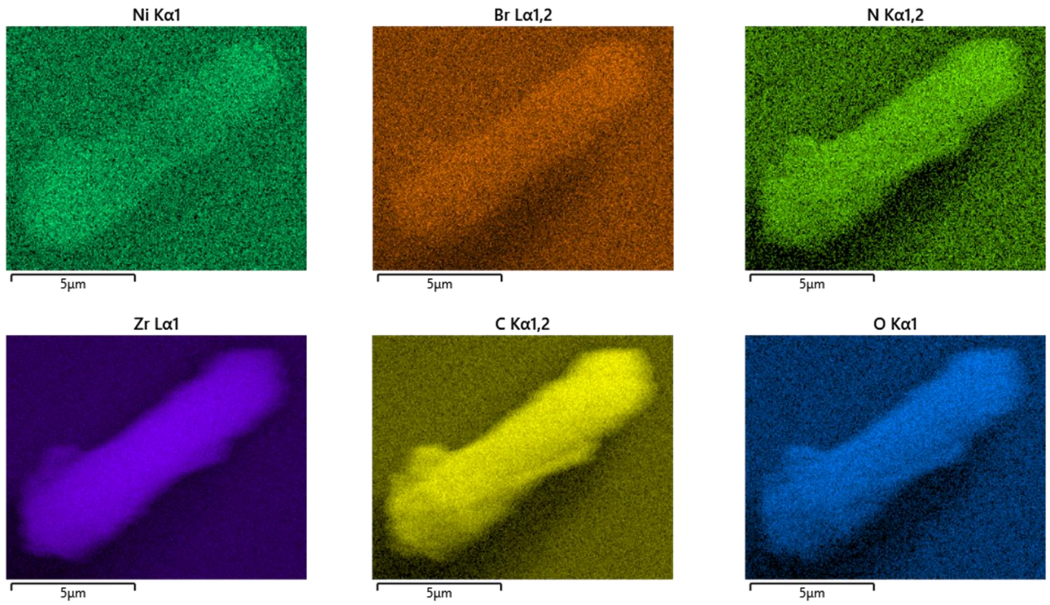

Figure S7. EDS mapping images of **NU-1000-Ni**. Ni, Br, N, Zr, C, and O are represented in jade green, bronze, yellowish green, purple, yellow, and blue, respectively.

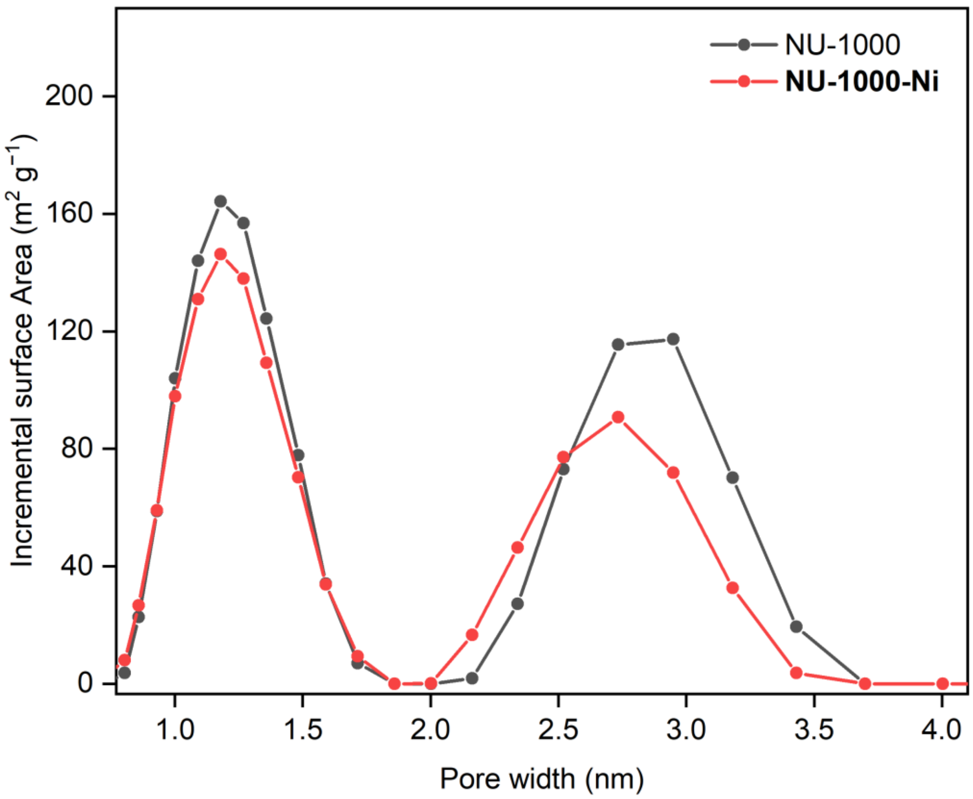

Figure S8. Pore width distributions.

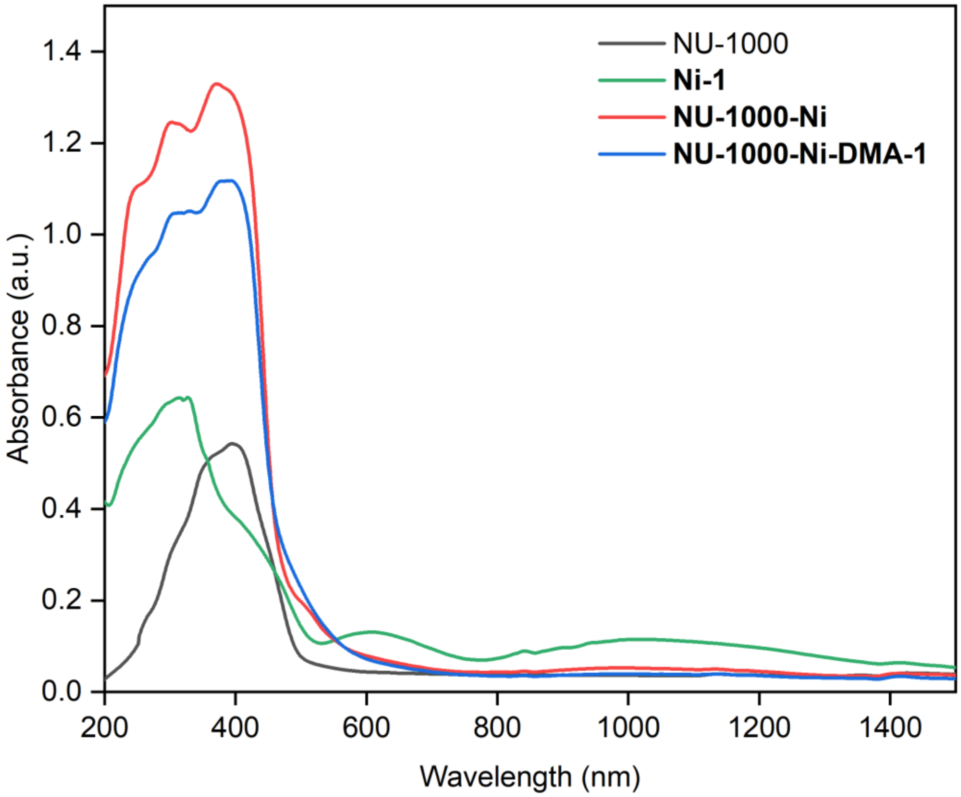

Figure S9. UV-Vis absorption spectra of **NU-1000-Ni-DMA-1** and its precursors.

Table S1. Summary of EXAFS fitting results at the Ni K-edge for NU-1000-Ni-DMF.

**NU-1000-Ni-DMF**		Fitting range	K: 3.0 – 11.5 Å^{-1}<br>R: 1.0 – 3.0 Å
Reduced Chi-square	564.8	R-factor	0.018
ΔE_0	– 7.10	S_0^2	0.98
σ^2Ni – N/O	0.003	R (Ni – N/O)	2.04 ± 0.01
σ^2Ni – Br	0.014	R (Ni – Br)	2.84 ± 0.03
Independent points	15.8	Number of variables	9
		CN (Ni – N/O)	4
		CN (Ni – Br)	2

CN: coordination numbers; R: bond distance; σ^2: Debye-Waller factors; ΔE_0: the inner potential correction; R factor: goodness of fit; S_0^2 was set as 0.98 for Ni data, which was obtained from the experimental EXAFS fit of Ni foil reference by fixing CN as the known crystallographic value and was fixed to all the samples.

4. Photoinduced sulfonylation of aryl halides catalyzed by heterogeneous catalysts

Table S2. Preliminary studies for reaction conditions.

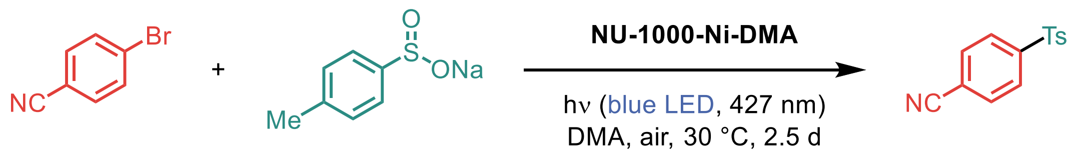

entry	variation from "standard condition"	conv./%[a]	yield/%[a]
1	none	>95	74
2	no light, r.t.	nd	nd
3	no light, 80 °C	10	9
4	390 nm, instead of 427 nm	>95	54
5	440 nm, instead of 427 nm	75	53
6	0.05 M, instead of 0.1 M	>95	67
7	0.2 M, instead of 0.1 M	>95	74
8	1.0 equiv. NaTs, instead of 3.0 euqiv.	71	43
9	2.0 equiv. NaTs, instead of 3.0 euqiv.	>95	70
10	0 °C, instead of 30 °C	>95	74
11	photoexcited for 1 h, then no light	7	6
12	photoexcited for 1 h under N_2, then no light	9	9

Condition: 4-bromobenzonitrile (0.2 mmol), sodium 4-methylbenzenesulfinate (3.0 equiv.), **NU-1000-Ni-DMA** (20 mg, 5 mol% Ni), DMA (2.0 mL). [a]Determined by ^{1}H NMR with CH_2Br_2 as an internal standard.

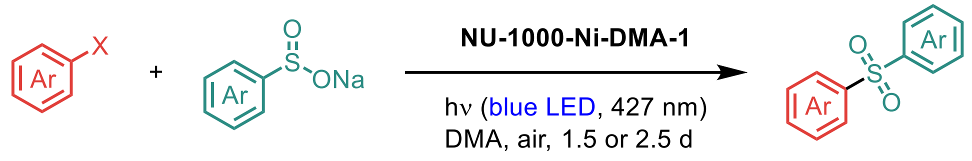

General Procedure: To Aryl halide (0.2 mmol) and sodium sulfinate (3.0 equiv., 0.6 mmol) was added **NU-1000-Ni-DMA-1** (2 mol% Ni) suspension in DMA (2.0 mL) under air. The reaction mixture was irradiated with blue LED light (Kessil PR-160, 427 nm, 40 W, three lamps) positioned approximately 3 cm away and stirred for 1.5 days (when using aryl iodides) or for 2.5 day (when using aryl bromides). A water-cooling system was used to control the reaction temperature to be at 30 °C. After the completion of the reaction, the reaction system was filtered, and the solids were washed with DMA (3 mL) 3 times. The washing DMA was combined with filtrate and extracted with hexanes-EA (1:1 v/v, 25 mL) and water (25 mL), and the aqueous phase was further extracted with hexanes-EA (1:1 v/v, 25 mL). All the organic phase was combined and further extracted with saturated brine (40 mL) to remove residue DMA. Finally, the organic phase was collected and concentrated under reduced pressure. The residue was purified by silica gel column chromatography and dried *in vacuo* to give pure product.

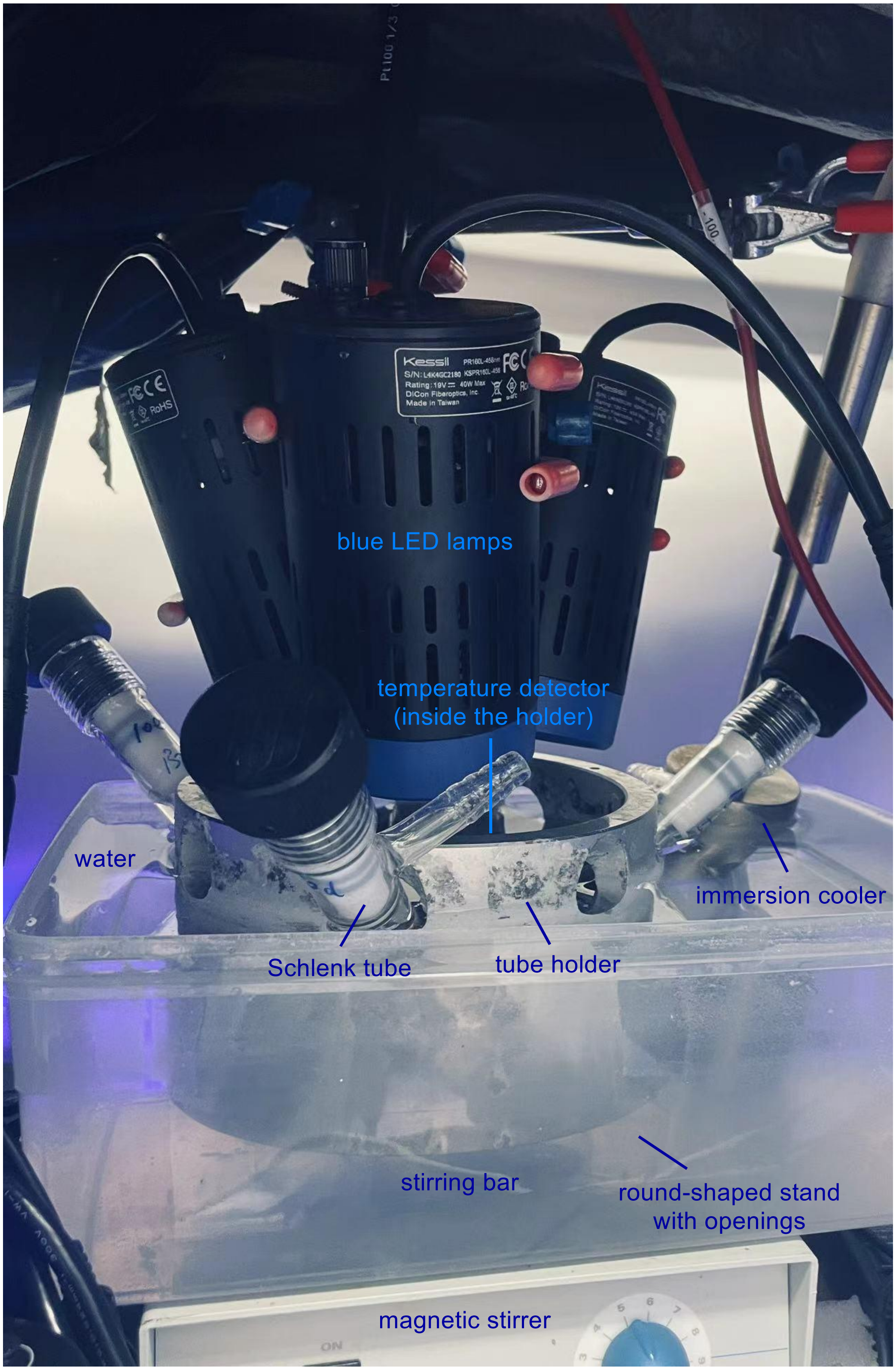

Figure S10. Photoreaction setup (lights off).

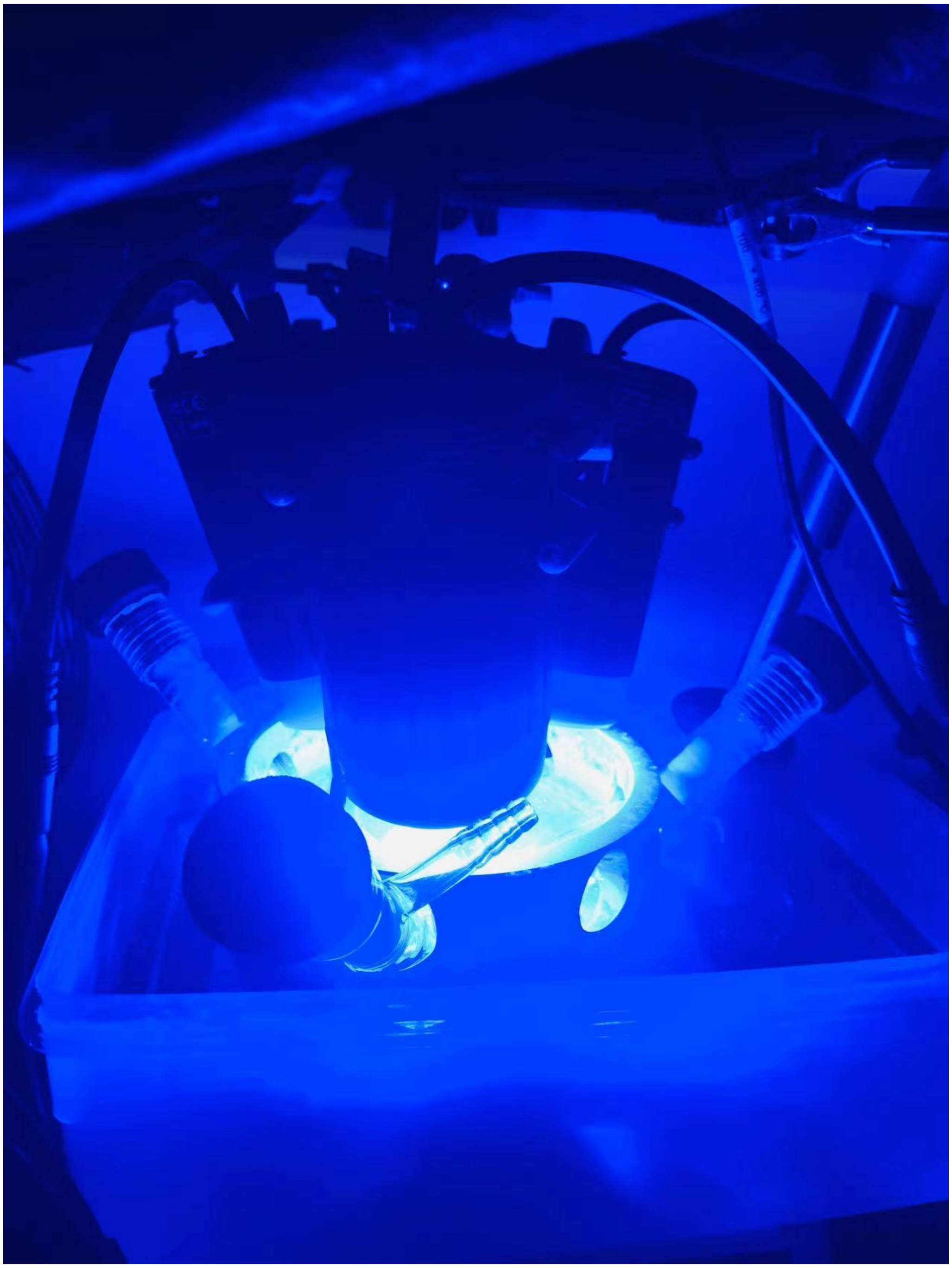

Figure S11. Photoreaction setup (lights on).

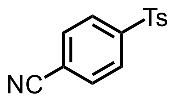

1

4-Tosylbenzonitrile (**1**) was prepared according to General Procedure with 4-iodobenzonitrile (1.0 equiv., 0.2 mmol, 45.8 mg) or 4-bromobenzonitrile (1.0 equiv., 0.2 mmol, 36.4 mg) and sodium 4-methylbenzenesulfinate (3.0 equiv., 0.6 mmol, 106.9 mg). The crude product was purified by silica gel column chromatography (eluent: hexanes-DCM = 2:1 to 1:1 v/v) to give the entitled compound (42.2 mg, 82% yield, when using aryl iodide; 30.9 mg, 60% yield, when using aryl bromide).

^{1}H NMR (400 MHz, $CDCl_3$) δ 8.03 (d, J = 8.4 Hz, 2H), 7.82 (d, J = 8.0 Hz, 2H), 7.78 (d, J = 8.4 Hz, 2H), 7.33 (d, J = 8.0 Hz, 2H), 2.42 (s, 3H).

^{13}C NMR (100 MHz, $CDCl_3$) δ 146.2, 145.2, 137.1, 133.0, 130.3, 128.1, 128.0, 117.2, 116.7, 21.6.

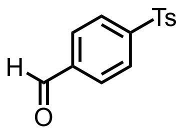

2

4-Tosylbenzaldehyde (**2**) was prepared according to General Procedure with 4-iodobenzaldehyde (1.0 equiv., 0.2 mmol, 46.4 mg) or 4-bromobenzonitrile (1.0 equiv., 0.2 mmol, 37.0 mg) and sodium 4-methylbenzenesulfinate (3.0 equiv., 0.6 mmol, 106.9 mg). The crude product was purified by silica gel column chromatography (eluent: hexanes-DCM = 1:1 v/v to pure DCM) to give the entitled compound (19.8 mg, 38% yield, when using aryl iodide; 22.9 mg, 44% yield, when using aryl bromide).

^{1}H NMR (400 MHz, $CDCl_3$) δ 10.06 (s, 1H), 8.08 (d, J = 8.0 Hz, 2H), 7.98 (d, J = 8.0 Hz, 2H), 7.84 (d, J = 8.0 Hz, 2H), 7.32 (d, J = 8.0 Hz, 2H), 2.40 (s, 3H).

^{13}C NMR (100 MHz, $CDCl_3$) δ 190.7, 147.0, 144.9, 139.0, 137.5, 130.24, 130.15, 128.2, 128.0, 21.6.

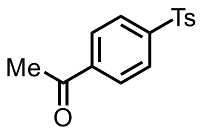

3

1-(4-Tosylphenyl)ethan-1-one (**3**) was prepared according to General Procedure with 1-(4-iodophenyl)ethan-1-one (1.0 equiv., 0.2 mmol, 49.2 mg) or 1-(4-bromophenyl)ethan-1-one (1.0 equiv., 0.2 mmol, 39.8 mg) and sodium 4-methylbenzenesulfinate (3.0 equiv., 0.6 mmol, 106.9 mg). The crude product was purified by silica gel column chromatography (eluent: hexanes-DCM = 1:1 v/v to pure DCM) to give the entitled compound (30.2 mg, 55% yield, when using aryl iodide; 25.2 mg, 46% yield, when using aryl bromide).

^{1}H NMR (400 MHz, $CDCl_3$) δ 8.07 – 7.97 (m, 4H), 7.83 (d, J = 8.0 Hz, 2H), 7.31 (d, J = 8.0 Hz, 2H), 2.60 (s, 3H), 2.39 (s, 3H).

^{13}C NMR (100 MHz, $CDCl_3$) δ 196.7, 145.8, 144.7, 140.1, 137.7, 130.1, 129.0, 127.9, 127.8, 26.8, 21.6.

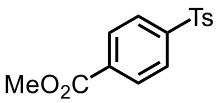

4

Methyl 4-tosylbenzoate (**4**) was prepared according to General Procedure with methyl 4-iodobenzoate (1.0 equiv., 0.2 mmol, 52.4 mg) or methyl 4-bromobenzoate (1.0 equiv., 0.2 mmol, 43.0 mg) and sodium 4-methylbenzenesulfinate (3.0 equiv., 0.6 mmol, 106.9 mg). The crude product was purified by silica gel column chromatography (eluent: hexanes-DCM = 1:1 v/v to pure DCM) to give the entitled compound (42.9 mg, 74% yield, when using aryl iodide; 29.0 mg, 50% yield, when using aryl bromide).

^{1}H NMR (400 MHz, $CDCl_3$) δ 8.12 (d, J = 8.0 Hz, 2H), 7.97 (d, J = 8.0 Hz, 2H), 7.82 (d, J = 8.0 Hz, 2H), 7.30 (d, J = 8.0 Hz, 2H), 3.92 (s, 3H), 2.39 (s, 3H).

^{13}C NMR (100 MHz, $CDCl_3$) δ 165.5, 145.8, 144.7, 137.8, 134.0, 130.3, 130.0, 127.8, 127.5, 52.6, 21.5.

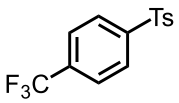

5

1-Methyl-4-((4-(trifluoromethyl)phenyl)sulfonyl)benzene (**5**) was prepared according to General Procedure with 1-iodo-4-(trifluoromethyl)benzene (1.0 equiv., 0.2 mmol, 54.4 mg) or 1-bromo-4-(trifluoromethyl)benzene (1.0 equiv., 0.2 mmol, 45.0 mg) and sodium 4-methylbenzenesulfinate (3.0 equiv., 0.6 mmol, 106.9 mg). The crude product was purified by silica gel column chromatography (eluent: hexanes-DCM = 2:1 to 1:1 v/v) to give the entitled compound (22.8 mg, 38% yield, when using aryl iodide; 18.0 mg, 30% yield, when using aryl bromide).

^{1}H NMR (400 MHz, $CDCl_3$) δ 8.05 (d, J = 8.0 Hz, 2H), 7.84 (d, J = 8.0 Hz, 2H), 7.75 (d, J = 8.0 Hz, 2H), 7.33 (d, J = 8.0 Hz, 2H), 2.41 (s, 3H).

^{13}C NMR (100 MHz, $CDCl_3$) δ 145.6, 144.9, 137.6, 134.6 (q, J = 32.9 Hz), 130.2, 128.0, 127.9, 126.4 (q, J = 3.8 Hz), 123.1 (q, J = 271.2 Hz), 21.6.

^{19}F NMR (376 MHz, $CDCl_3$) δ −63.2.

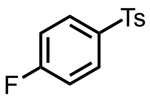

6

1-Fluoro-4-tosylbenzene (**6**) was prepared according to General Procedure with 1-fluoro-4-iodobenzene (1.0 equiv., 0.2 mmol, 54.4 mg) and sodium 4-methylbenzenesulfinate (3.0 equiv., 0.6 mmol, 106.9 mg). The crude product was purified by silica gel column chromatography (eluent: hexanes-DCM = 1:1 v/v) to give the entitled compound (25.0 mg, 50% yield).

^{1}H NMR (400 MHz, $CDCl_3$) δ 7.99 – 7.88 (m, 2H), 7.81 (d, J = 8.0 Hz, 2H), 7.30 (d, J = 8.0 Hz, 2H), 7.20 – 7.08 (m, 2H), 2.39 (s, 3H).

^{13}C NMR (100 MHz, $CDCl_3$) δ 165.3 (d, J = 253.9 Hz), 144.3, 138.5, 138.0 (d, J = 3.3 Hz), 130.3 (d, J = 9.4 Hz), 130.0, 127.6, 116.5 (d, J = 22.4 Hz), 21.5.

^{19}F NMR (376 MHz, $CDCl_3$) δ −104.6.

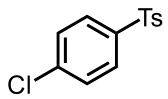

7

1-Chloro-4-tosylbenzene (**7**) was prepared according to General Procedure with 1-chloro-4-iodobenzene (1.0 equiv., 0.2 mmol, 47.7 mg) and sodium 4-methylbenzenesulfinate (3.0 equiv., 0.6 mmol, 106.9 mg). The crude product was purified by silica gel column chromatography (eluent: hexanes-DCM = 1:1 v/v to DCM) to give the entitled compound (44.8 mg, 84% yield).

^{1}H NMR (400 MHz, $CDCl_3$) δ 7.86 (d, J = 8.4 Hz, 2H), 7.80 (d, J = 8.0 Hz, 2H), 7.45 (d, J = 8.4 Hz, 2H), 7.30 (d, J = 8.0 Hz, 2H), 2.40 (s, 3H).

^{13}C NMR (100 MHz, $CDCl_3$) δ 144.5, 140.5, 139.6, 138.2, 130.0, 129.5, 128.9, 127.7, 21.6.

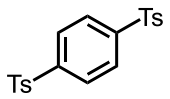

8

1,4-Ditosylbenzene (**8**) was prepared according to General Procedure with 1,4-diiodobenzene (1.0 equiv., 0.2 mmol, 66.0 mg) or 1,4-dibromobenzene (1.0 equiv., 0.2 mmol, 47.2 mg) and sodium 4-methylbenzenesulfinate (3.0 equiv., 0.6 mmol, 106.9 mg). The crude product was purified by silica gel column chromatography (eluent: hexanes-DCM = 1:1 v/v to pure DCM) to give the entitled compound (24.7 mg, 32% yield, when using 1,4-diiodobenzene; 32.5 mg, 42% yield, when using 1-bromo-4-iodobenzene with 5.0 equiv. of sodium 4-methylbenzenesulfinate).

^{1}H NMR (400 MHz, $CDCl_3$) δ 8.01 (s, 4H), 7.80 (d, J = 8.0 Hz, 4H), 7.31 (d, J = 8.0 Hz, 4H), 2.40 (s, 6H).
^{13}C NMR (100 MHz, $CDCl_3$) δ 146.2, 145.1, 137.2, 130.2, 128.3, 128.0, 21.6.

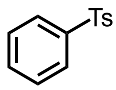

9

1-Methyl-4-(phenylsulfonyl)benzene (**9**) was prepared according to General Procedure with iodobenzene (1.0 equiv., 0.2 mmol, 40.8 mg) and sodium 4-methylbenzenesulfinate (3.0 equiv., 0.6 mmol, 106.9 mg). The crude product was purified by silica gel column chromatography (eluent: hexanes-DCM = 1:1 v/v to pure DCM) to give the entitled compound (25.1 mg, 54% yield).

^{1}H NMR (400 MHz, $CDCl_3$) δ 7.93 (d, J = 7.6 Hz, 2H), 7.83 (d, J = 8.0 Hz, 2H), 7.57 – 7.42 (m, 3H), 7.29 (d, J = 8.0 Hz, 2H), 2.39 (s, 3H).

^{13}C NMR (100 MHz, $CDCl_3$) δ 144.1, 142.0, 138.6, 133.0, 129.9, 129.2, 127.7, 127.5, 21.5.

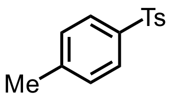

10

4,4'-Sulfonylbis(methylbenzene) (**10**) was prepared according to General Procedure with 1-iodo-4-methylbenzene (1.0 equiv., 0.2 mmol, 43.6 mg) and sodium 4-methylbenzenesulfinate (3.0 equiv., 0.6 mmol, 106.9 mg). The crude product was purified by silica gel column chromatography (eluent: hexanes-DCM = 2:1 to 1:1 v/v) to give the entitled compound (23.2 mg, 47% yield).

^{1}H NMR (400 MHz, $CDCl_3$) δ 7.83 (d, J = 8.0 Hz, 4H), 7.30 (d, J = 8.0 Hz, 4H), 2.41 (s, 6H).

^{13}C NMR (100 MHz, $CDCl_3$) δ 143.9, 139.0, 129.8, 127.5, 21.5.

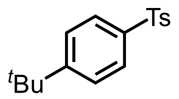

11

1-(*tert*-Butyl)-4-tosylbenzene (**11**) was prepared according to General Procedure with 1-(tert-butyl)-4-iodobenzene (1.0 equiv., 0.2 mmol, 52.0 mg) and sodium 4-methylbenzenesulfinate (3.0 equiv., 0.6 mmol, 106.9 mg). The crude product was purified by silica gel column chromatography (eluent: hexanes-DCM = 2:1 to 1:1 v/v) to give the entitled compound (24.8 mg, 43% yield).

^{1}H NMR (400 MHz, $CDCl_3$) δ 7.87 – 7.80 (m, 4H), 7.49 (d, J = 8.0 Hz, 2H), 7.29 (d, J = 8.0 Hz, 2H), 2.39 (s, 3H), 1.30 (s, 9H).

^{13}C NMR (100 MHz, $CDCl_3$) δ 156.8, 143.9, 139.0, 138.9, 129.8, 127.6, 127.3, 126.2, 35.1, 31.0, 21.5.

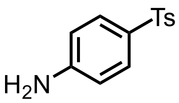

12

4-Tosylaniline (**12**) was prepared according to General Procedure with 4-iodoaniline (1.0 equiv., 0.2 mmol, 43.8 mg) and sodium 4-methylbenzenesulfinate (3.0 equiv., 0.6 mmol, 106.9 mg). The crude product was purified by silica gel column chromatography (eluent: pure DCM to DCM-MeOH = 100:1 v/v, with 2 drops of Et_3N per 500 mL solvent) to give the entitled compound (30.2 mg, 61% yield).

^{1}H NMR (400 MHz, $CDCl_3$) δ 7.77 (d, J = 8.0 Hz, 2H), 7.68 (d, J = 8.4 Hz, 2H), 7.25 (d, J = 8.0 Hz, 2H), 6.64 (d, J = 8.4 Hz, 2H), 4.14 (br, 2H), 2.37 (s, 3H).

^{13}C NMR (100 MHz, $CDCl_3$) δ 150.8, 143.3, 140.0, 129.9, 129.7 (2C), 127.1, 114.1, 21.5.

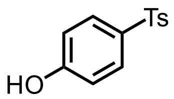

13

4-Tosylphenol (**13**) was prepared according to General Procedure with 4-iodophenol (1.0 equiv., 0.2 mmol, 44.0 mg) and sodium 4-methylbenzenesulfinate (3.0 equiv., 0.6 mmol, 106.9 mg). The crude product was purified by silica gel column chromatography (eluent: pure DCM to DCM-MeOH = 100:1 v/v) to give the entitled compound (19.9 mg, 40% yield).

^{1}H NMR (400 MHz, $CDCl_3$) δ 7.82 – 7.73 (m, 4H), 7.27 (d, J = 8.8 Hz, 2H), 6.89 (d, J = 8.8 Hz, 2H), 6.46 (br, 1H), 2.38 (s, 3H).

^{13}C NMR (100 MHz, $CDCl_3$) δ 160.5, 143.9, 139.0, 132.7, 129.9, 129.8, 127.2, 116.1, 21.5.

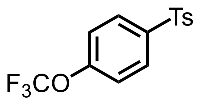

14

1-Methyl-4-((4-(trifluoromethoxy)phenyl)sulfonyl)benzene (**14**) was prepared according to General Procedure with 1-iodo-4-(trifluoromethoxy)benzene (1.0 equiv., 0.2 mmol, 57.9 mg) and sodium 4-methylbenzenesulfinate (3.0 equiv., 0.6 mmol, 106.9 mg). The crude product was purified by silica gel column chromatography (eluent: hexanes-DCM = 2:1 v/v) to give the entitled compound (34.2 mg, 54% yield).

^{1}H NMR (400 MHz, $CDCl_3$) δ 7.97 (d, J = 8.8 Hz, 2H), 7.82 (d, J = 8.0 Hz, 2H), 7.35 – 7.27 (m, 4H), 2.40 (s, 3H).

^{13}C NMR (100 MHz, $CDCl_3$) δ 152.4 (q, J = 1.8 Hz), 144.6, 140.3, 138.1, 130.1, 129.7, 127.7, 121.0, 120.1 (q, J = 257.9 Hz), 21.5.

^{19}F NMR (376 MHz, $CDCl_3$) δ −57.7.

HRMS (ESI) calculated for $C_{14}H_{12}F_3O_3S^+$ $[M+H]^+$: 317.0454, found: 317.0456.

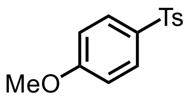

15

1-Methoxy-4-tosylbenzene (**15**) was prepared according to General Procedure with 1-iodo-4-methoxybenzene (1.0 equiv., 0.2 mmol, 46.8 mg) and sodium 4-methylbenzenesulfinate (3.0 equiv., 0.6 mmol, 106.9 mg). The crude product was purified by silica gel column chromatography (eluent: hexanes-DCM = 1:1 v/v to pure DCM) to give the entitled compound (32.0 mg, 61% yield).

^{1}H NMR (400 MHz, $CDCl_3$) δ 7.88 (d, J = 8.4 Hz, 2H), 7.81 (d, J = 8.0 Hz, 2H), 7.29 (d, J = 8.0 Hz, 2H), 6.97 (d, J = 8.4 Hz, 2H), 3.85 (s, 3H), 2.40 (s, 3H).

^{13}C NMR (100 MHz, $CDCl_3$) δ 163.2, 143.7, 139.4, 133.5, 129.8, 129.7, 127.3, 114.4, 55.6, 21.5.

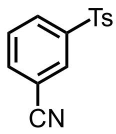

16

3-Tosylbenzonitrile (**16**) was prepared according to General Procedure with 3-iodobenzonitrile (1.0 equiv., 0.2 mmol, 45.8 mg) and sodium 4-methylbenzenesulfinate (3.0 equiv., 0.6 mmol, 106.9 mg). The crude product was purified by silica gel column chromatography (eluent: hexanes-DCM = 1:1 v/v to pure DCM) to give the entitled compound (32.9 mg, 64% yield).

^{1}H NMR (400 MHz, $CDCl_3$) δ 8.18 (s, 1H), 8.15 (d, J = 8.0 Hz, 1H), 7.86 – 7.77 (m, 3H), 7.67 – 7.60 (m, 1H), 7.34 (d, J = 8.0 Hz, 2H), 2.42 (s, 3H).

^{13}C NMR (100 MHz, $CDCl_3$) δ 145.2, 143.8, 137.1, 136.0, 131.3, 131.1, 130.3 (2C), 127.9, 117.0, 113.8, 21.6.

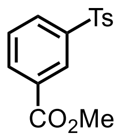

17

Methyl 3-tosylbenzoate (**17**) was prepared according to General Procedure with methyl 3-iodobenzoate (1.0 equiv., 0.2 mmol, 52.4 mg) and sodium 4-methylbenzenesulfinate (3.0 equiv., 0.6 mmol, 106.9 mg). The crude product was purified by silica gel column chromatography (eluent: hexanes-DCM = 1:1 v/v to DCM) to give the entitled compound (38.3 mg, 66% yield).

^{1}H NMR (400 MHz, $CDCl_3$) δ 8.57 (s, 1H), 8.20 (d, J = 7.6 Hz, 1H), 8.11 (d, J = 7.6 Hz, 1H), 7.84 (d, J = 8.0 Hz, 2H), 7.60 – 7.56 (m, 1H), 7.30 (d, J = 8.0 Hz, 2H), 3.93 (s, 3H), 2.39 (s, 3H).

^{13}C NMR (100 MHz, $CDCl_3$) δ 165.3, 144.6, 142.7, 138.0, 133.8, 131.5, 130.0, 129.5, 128.6, 127.8, 52.6, 21.5.

HRMS (ESI) calculated for $C_{15}H_{15}O_4S$ $[M+H]^+$: 291.0686, found: 291.0688.

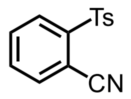

18

2-Tosylbenzonitrile (**18**) was prepared according to General Procedure with 3-iodobenzonitrile (1.0 equiv., 0.2 mmol, 45.8 mg) and sodium 4-methylbenzenesulfinate (3.0 equiv., 0.6 mmol, 106.9 mg). The crude product was purified by silica gel column chromatography (eluent: hexanes-DCM = 2:1 to 1:2 v/v) to give the entitled compound (11.8 mg, 23% yield).

^{1}H NMR (400 MHz, $CDCl_3$) δ 8.33 (d, J = 7.8 Hz, 1H), 7.96 (d, J = 8.0 Hz, 2H), 7.84 – 7.75 (m, 2H), 7.71 – 7.63 (m, 1H), 7.35 (d, J = 8.0 Hz, 2H), 2.42 (s, 3H).

^{13}C NMR (100 MHz, $CDCl_3$) δ 145.4, 144.1, 136.5, 135.6, 133.2, 133.1, 130.0, 129.6, 128.7, 115.6, 111.3, 21.7.

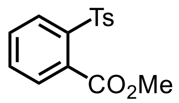

19

Methyl 2-tosylbenzoate (**19**) was prepared according to General Procedure with methyl 3-iodobenzoate (1.0 equiv., 0.2 mmol, 52.4 mg) and sodium 4-methylbenzenesulfinate (3.0 equiv., 0.6 mmol, 106.9 mg). The crude product was purified by silica gel column chromatography (eluent: hexanes-DCM = 1:1 v/v to pure DCM) to give the entitled compound (43.0 mg, 74% yield).

^{1}H NMR (400 MHz, $CDCl_3$) δ 8.16 – 8.06 (m, 1H), 7.85 (d, J = 8.0 Hz, 2H), 7.64 – 7.57 (m, 2H), 7.57 – 7.49 (m, 1H), 7.30 (d, J = 8.0 Hz, 2H), 3.93 (s, 3H), 2.39 (s, 3H).

^{13}C NMR (100 MHz, $CDCl_3$) δ 167.7, 144.2, 139.3, 138.4, 133.0 (2C), 130.8, 130.0, 129.5, 129.0, 127.9, 53.0, 21.5.

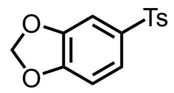

20

5-Tosylbenzo[*d*][1,3]dioxole (**20**) was prepared according to General Procedure with 5-iodobenzo[*d*][1,3]dioxole (1.0 equiv., 0.2 mmol, 49.6 mg) and sodium 4-methylbenzenesulfinate (3.0 equiv., 0.6 mmol, 106.9 mg). The crude product was purified by silica gel column chromatography (eluent: hexanes-DCM = 2:1 to 1:1 v/v) to give the entitled compound (19.9 mg, 36% yield).

^{1}H NMR (400 MHz, $CDCl_3$) δ 7.78 (d, J = 8.0 Hz, 2H), 7.52 (d, J = 8.0 Hz, 1H), 7.34 – 7.25 (m, 3H), 6.86 (d, J = 8.0 Hz, 1H), 6.03 (s, 2H), 2.39 (s, 3H).

^{13}C NMR (100 MHz, $CDCl_3$) δ 151.7, 148.3, 143.9, 139.0, 135.3, 129.8, 127.4, 123.3, 108.5, 107.7, 102.3, 21.5.

HRMS (ESI) calculated for $C_{14}H_{13}O_4S^+$ $[M+H]^+$: 277.0529, found: 277.0529.

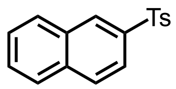

21

2-Tosylnaphthalene (**21**) was prepared according to General Procedure with 2-iodonaphthalene (1.0 equiv., 0.2 mmol, 50.8 mg) and sodium 4-methylbenzenesulfinate (3.0 equiv., 0.6 mmol, 106.9 mg). The crude product was purified by silica gel column chromatography (eluent: hexanes-DCM = 1:1 v/v to pure DCM) to give the entitled compound (36.1 mg, 64% yield).

^{1}H NMR (400 MHz, $CDCl_3$) δ 8.56 (s, 1H), 7.97 (d, J = 7.6 Hz, 1H), 7.93 – 7.81 (m, 5H), 7.66 – 7.55 (m, 2H), 7.29 (d, J = 8.0 Hz, 2H), 2.38 (s, 3H).

^{13}C NMR (100 MHz, $CDCl_3$) δ 144.1, 138.7, 138.6, 134.9, 132.2, 129.9, 129.5, 129.3, 129.0, 128.8, 127.9, 127.7, 127.5, 122.6, 21.5.

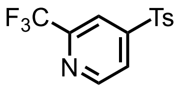

22

4-Tosyl-2-(trifluoromethyl)pyridine (**22**) was prepared according to General Procedure with 4-iodo-2-(trifluoromethyl)pyridine (1.0 equiv., 0.2 mmol, 54.5 mg) and sodium 4-methylbenzenesulfinate (3.0 equiv., 0.6 mmol, 106.9 mg). The crude product was purified by silica gel column chromatography (eluent: hexanes-DCM = 2:1 to 1:1 v/v, with 2 drops of Et_3N per 500 mL) to give the entitled compound (42.2 mg, 70% yield).

^{1}H NMR (400 MHz, $CDCl_3$) δ 8.92 (d, J = 5.2 Hz, 1H), 8.10 (s, 1H), 7.95 (d, J = 5.2 Hz, 1H), 7.86 (d, J = 8.0 Hz, 2H), 7.38 (d, J = 8.0 Hz, 2H), 2.44 (s, 3H).

^{13}C NMR (100 MHz, $CDCl_3$) δ 152.2, 151.5, 149.8 (q, J = 35.5 Hz), 146.1, 135.8, 130.5, 128.4, 123.4, 120.7 (q, J = 272.8 Hz), 117.7 (q, J = 2.8 Hz), 21.7.

^{19}F NMR (376 MHz, $CDCl_3$) δ −68.1.

HRMS (ESI) calculated for $C_{13}H_{11}F_3NO_2S^+$ $[M+H]^+$: 302.0457, found: 302.0458.

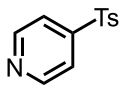

23

4-Tosylpyridine (**23**) was prepared according to General Procedure with 4-iodopyridine (1.0 equiv., 0.2 mmol, 41.0 mg) and sodium 4-methylbenzenesulfinate (3.0 equiv., 0.6 mmol, 106.9 mg). The crude product was purified by silica gel column chromatography (eluent: DCM-MeOH = 100:1 v/v, with 2 drops of Et_3N per 500 mL) to give the entitled compound (12.1 mg, 26% yield).

^{1}H NMR (400 MHz, $CDCl_3$) δ 8.85 – 8.76 (m, 2H), 7.84 (d, J = 8.4 Hz, 2H), 7.77 – 7.71 (m, 2H), 7.35 (d, J = 8.4 Hz, 2H), 2.43 (s, 3H).

^{13}C NMR (100 MHz, $CDCl_3$) δ 151.1, 150.1, 145.4, 136.6, 130.3, 128.2, 120.5, 21.6.

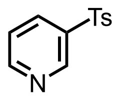

24

3-Tosylpyridine (**24**) was prepared according to General Procedure with 3-iodopyridine (1.0 equiv., 0.2 mmol, 41.0 mg) and sodium 4-methylbenzenesulfinate (3.0 equiv., 0.6 mmol, 106.9 mg). The crude product was purified by silica gel column chromatography (eluent: hexanes-DCM = 1:1 v/v to pure DCM, with 2 drops of Et_3N per 500 mL) to give the entitled compound (20.1 mg, 43% yield).

^{1}H NMR (400 MHz, $CDCl_3$) δ 9.12 (s, 1H), 8.76 (d, J = 4.8 Hz, 1H), 8.19 (d, J = 8.0 Hz, 1H), 7.84 (d, J = 8.0 Hz, 2H), 7.43 (dd, J = 8.0, 4.8 Hz, 1H), 7.33 (d, J = 8.0 Hz, 2H), 2.41 (s, 3H).

^{13}C NMR (100 MHz, $CDCl_3$) δ 153.4, 148.6, 145.0, 138.6, 137.7, 135.0, 130.2, 127.8, 123.8, 21.6.

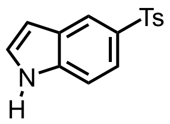

25

5-Tosyl-1*H*-indole (**25**) was prepared according to General Procedure with 5-iodo-1*H*-indole (1.0 equiv., 0.2 mmol, 48.6 mg) and sodium 4-methylbenzenesulfinate (3.0 equiv., 0.6 mmol, 106.9 mg). The crude product was purified by silica gel column chromatography (eluent: hexanes-DCM = 1:1 v/v to pure DCM, with 2 drops of Et_3N per 500 mL) to give the entitled compound (27.1 mg, 50% yield).

^{1}H NMR (400 MHz, $CDCl_3$) δ 8.74 (br s, 1H), 8.30 (s, 1H), 7.83 (d, J = 8.0 Hz, 2H), 7.69 (d, J = 8.4 Hz, 1H), 7.43 (d, J = 8.4 Hz, 1H), 7.32 – 7.31 (m, 1H), 7.25 (d, J = 8.0 Hz, 2H), 6.65 – 6.64 (m, 1H), 2.36 (s, 3H).

^{13}C NMR (100 MHz, $CDCl_3$) δ 143.4, 139.9, 137.8, 132.7, 129.7, 127.5, 127.3, 126.7, 121.7, 120.8, 111.8, 104.0, 21.5.

HRMS (ESI) calculated for $C_{15}H_{14}NO_2S^+$ $[M + H]^+$: 272.0740, found: 272.0743.

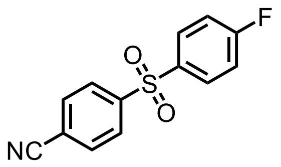

26

4-((4-Fluorophenyl)sulfonyl)benzonitrile (**26**) was prepared according to General Procedure with 4-iodobenzonitrile (1.0 equiv., 0.2 mmol, 45.8 mg) and sodium 4-fluorobenzenesulfinate (3.0 equiv., 0.6 mmol, 109.3 mg). The crude product was purified by silica gel column chromatography (eluent: hexanes-DCM = 2:1 to 1:1 v/v to pure DCM) to give the entitled compound (38.1 mg, 73% yield).

^{1}H NMR (400 MHz, $CDCl_3$) δ 8.04 (d, J = 8.0 Hz, 2H), 8.00 – 7.90 (m, 2H), 7.81 (d, J = 8.0 Hz, 2H), 7.25 – 7.17 (m, 2H).

^{13}C NMR (100 MHz, $CDCl_3$) δ 165.9 (d, J = 256.2 Hz), 145.7, 136.2 (d, J = 3.3 Hz), 133.1, 130.9 (d, J = 9.7 Hz), 128.2, 117.1, 117.04 (d, J =22.7 Hz), 117.03.

^{19}F NMR (376 MHz, $CDCl_3$) δ −102.3.

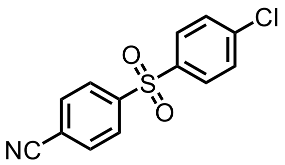

27

4-((4-Chlorophenyl)sulfonyl)benzonitrile (**27**) was prepared according to General Procedure with 4-iodobenzonitrile (1.0 equiv., 0.2 mmol, 45.8 mg) and sodium 4-chlorobenzenesulfinate (3.0 equiv., 0.6 mmol, 119.2 mg). The crude product was purified by silica gel column chromatography (eluent: hexanes-DCM = 2:1 to 1:1 v/v to pure DCM) to give the entitled compound (32.2 mg, 58% yield).

^{1}H NMR (400 MHz, $CDCl_3$) δ 8.04 (d, J = 8.4 Hz, 2H), 7.88 (d, J = 8.4 Hz, 2H), 7.81 (d, J = 8.4 Hz, 2H), 7.52 (d, J = 8.4 Hz, 2H).

^{13}C NMR (100 MHz, $CDCl_3$) δ 145.4, 140.9, 138.6, 133.2, 130.0, 129.4, 128.2, 117.1, 117.0.

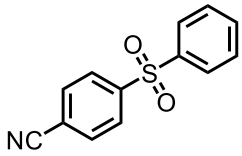

28

4-(Phenylsulfonyl)benzonitrile (**28**) was prepared according to General Procedure with 4-iodobenzonitrile (1.0 equiv., 0.2 mmol, 45.8 mg) and sodium benzenesulfinate (3.0 equiv., 0.6 mmol, 98.5 mg). The crude product was purified by silica gel column chromatography (eluent: hexanes-DCM = 2:1 to 1:1 v/v to pure DCM) to give the entitled compound (39.9 mg, 82% yield).

^{1}H NMR (400 MHz, $CDCl_3$) δ 8.05 (d, J = 8.3 Hz, 2H), 7.94 (d, J = 7.2 Hz, 2H), 7.79 (d, J = 8.3 Hz, 2H), 7.67 – 7.58 (m, 1H), 7.58 – 7.49 (m, 2H).

^{13}C NMR (100 MHz, $CDCl_3$) δ 145.8, 140.0, 134.0, 133.0, 129.6, 128.2, 127.9, 117.1, 116.8.

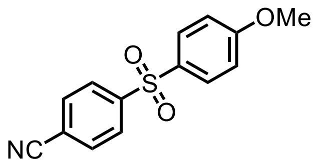

29

4-((4-Methoxyphenyl)sulfonyl)benzonitrile (**29**) was prepared according to General Procedure with 4-iodobenzonitrile (1.0 equiv., 0.2 mmol, 45.8 mg) and sodium 4-methoxybenzenesulfinate (3.0 equiv., 0.6 mmol, 116.5 mg). The crude product was purified by silica gel column chromatography (eluent: hexanes-DCM = 2:1 to 1:1 v/v to pure DCM) to give the entitled compound (25.1 mg, 46% yield).

^{1}H NMR (400 MHz, $CDCl_3$) δ 8.01 (d, J = 8.4 Hz, 2H), 7.87 (d, J = 8.8 Hz, 2H), 7.77 (d, J = 8.4 Hz, 2H), 6.99 (d, J = 8.8 Hz, 2H), 3.85 (s, 3H).

^{13}C NMR (100 MHz, $CDCl_3$) δ 164.0, 146.6, 133.0, 131.4, 130.2, 127.9, 117.2, 116.5, 114.9, 55.7.

Gram-scale reaction

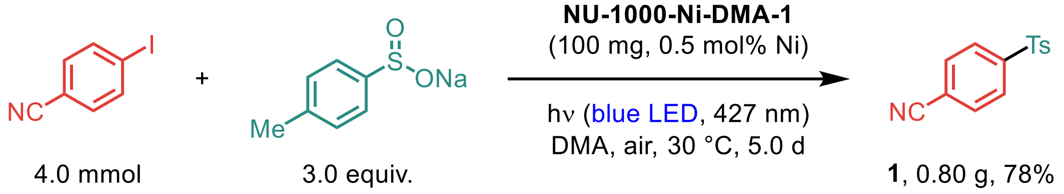

To 4-iodobenzonitrile (4.0 mmol, 0.92 g) and sodium 4-methylbenzenesulfinate (3.0 equiv., 12.0 mmol, 2.14 g) was added **NU-1000-Ni-DMA-1** (0.5 mol% Ni, 100 mg) suspension in DMA (20 mL) under air. The reaction mixture was irradiated by blue LED light (Kessil PR-160, 427 nm, 40 W, 3 lamps) for 5.0 d. A water-cooling system was used to control the reaction temperature to be at 30 °C. After the completion of the reaction, the reaction system was filtered, and the solids were washed with DMA (20 mL) 3 times. The washing DMA was combined with the filtrate and extracted with hexanes-EA (1:1 v/v, 150 mL), and the aqueous phase was extracted with hexanes-EA (1:1 v/v, 150 mL) again. All the organic phase was combined and further extracted with saturated brine (200 mL) to remove residue DMA. Finally, the organic phase was collected and concentrated under reduced pressure, and the residue was purified by silica gel column chromatography (eluent: hexanes-EA = 2:1 to 1:1 v/v) to give pure product **1** (0.80 g, 78% yield).

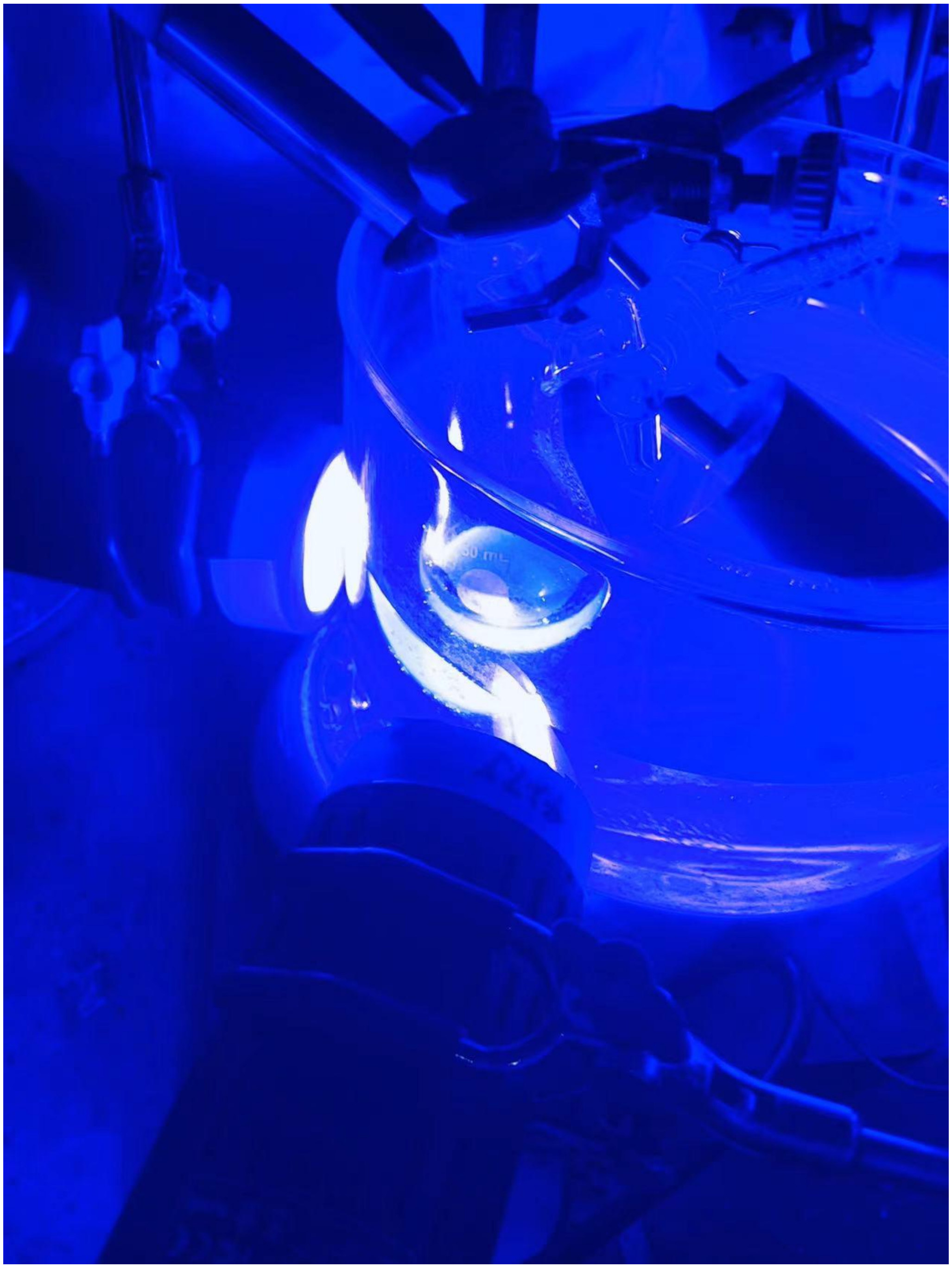

Figure S12. Gram-scale photoreaction setup (lights on).

Recycling experiments

After the completion of the reaction, the reaction system was filtered with filtering paper, and the collected heterogeneous catalyst was washed 3 times with DMA (3 mL). The recycled heterogeneous catalyst was washed down and made into its suspension with DMA (2 mL), and the suspension could be directly used for the next cycle.

Table S3. Recycling experiments.

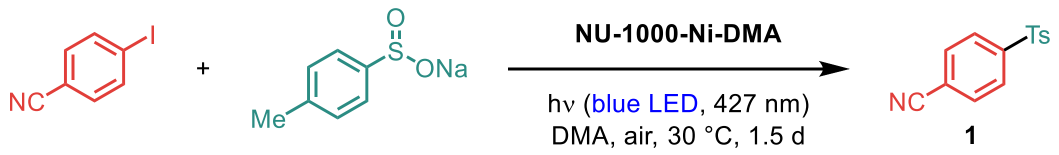

cycle	yield[a] (%)
1	85
2	95
3	95
4	93
5	94
6	95
7	90
8	88

Condition: 4-iodobenzonitrile (0.2 mmol), sodium 4-methylbenzenesulfinate (3.0 equiv.), **NU-1000-Ni-DMA** (20 mg, 5 mol% Ni), DMA (2.0 mL). [a]Determined by ^{1}H NMR with CH_2Br_2 as internal standard.

5. References

1 Yue, H., Zhu, C. & Rueping, M. Cross-coupling of sodium sulfinates with aryl, heteroaryl, and vinyl halides by nickel/photoredox dual catalysis. *Angew. Chem. Int. Ed.* **57**, 1371−1375 (2018).

2 Yan, Q. *et al.* Sulfonylation of aryl halides by visible light/copper catalysis. *Org. Lett.* **23**, 3663−3668 (2021).

3 Cavedon, C. *et al.* Intraligand charge transfer enables visible-light-mediated nickel-catalyzed cross-coupling reactions. *Angew. Chem. Int. Ed.* **61**, e202211433 (2022).

4 Zhu, Y. Y. *et al.* Merging photoredox and organometallic catalysts in a metal-organic framework significantly boosts photocatalytic activities. *Angew. Chem. Int. Ed.* **57**, 14090−14094 (2018).

5 Gao, Y. *et al.* Visible-light-induced nickel-catalyzed cross-coupling with alkylzirconocenes from unactivated alkenes. *Chem* **6**, 675−688 (2020).

6 Wang, T. C. *et al.* Scalable synthesis and post-modification of a mesoporous metal-organic framework called NU-1000. *Nat. Protoc.* **11**, 149−162 (2016).

6. NMR spectra

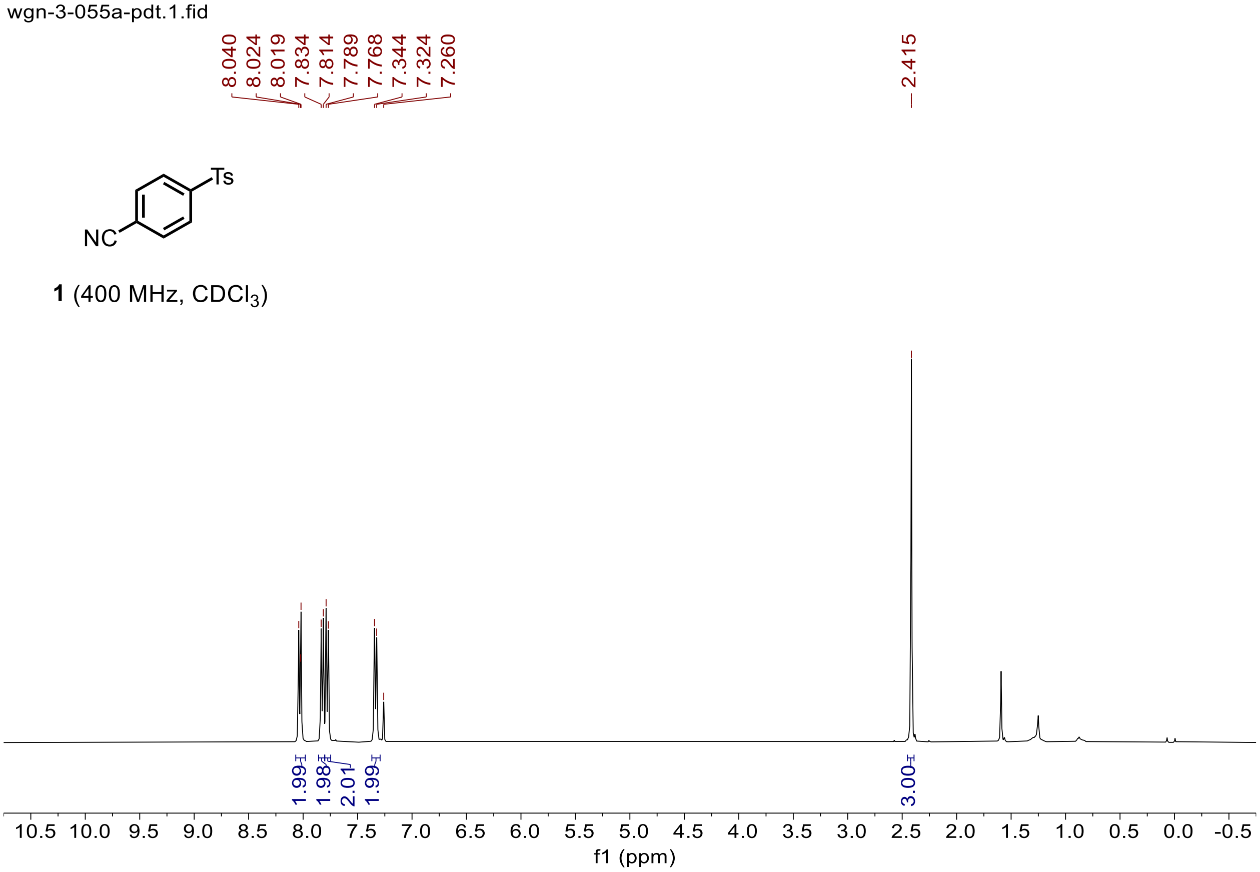

wgn-3-055a-pdt-C.1.fid

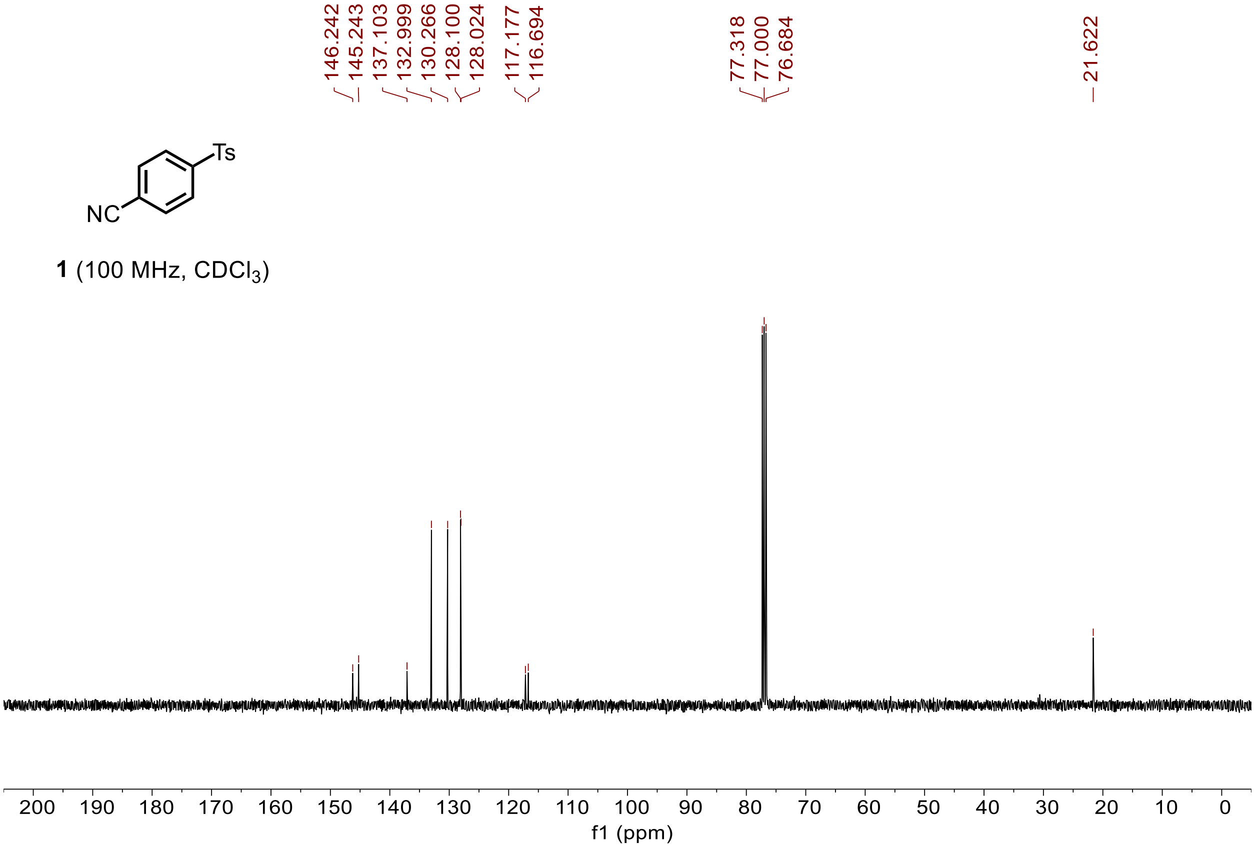

1 (100 MHz, $CDCl_3$)

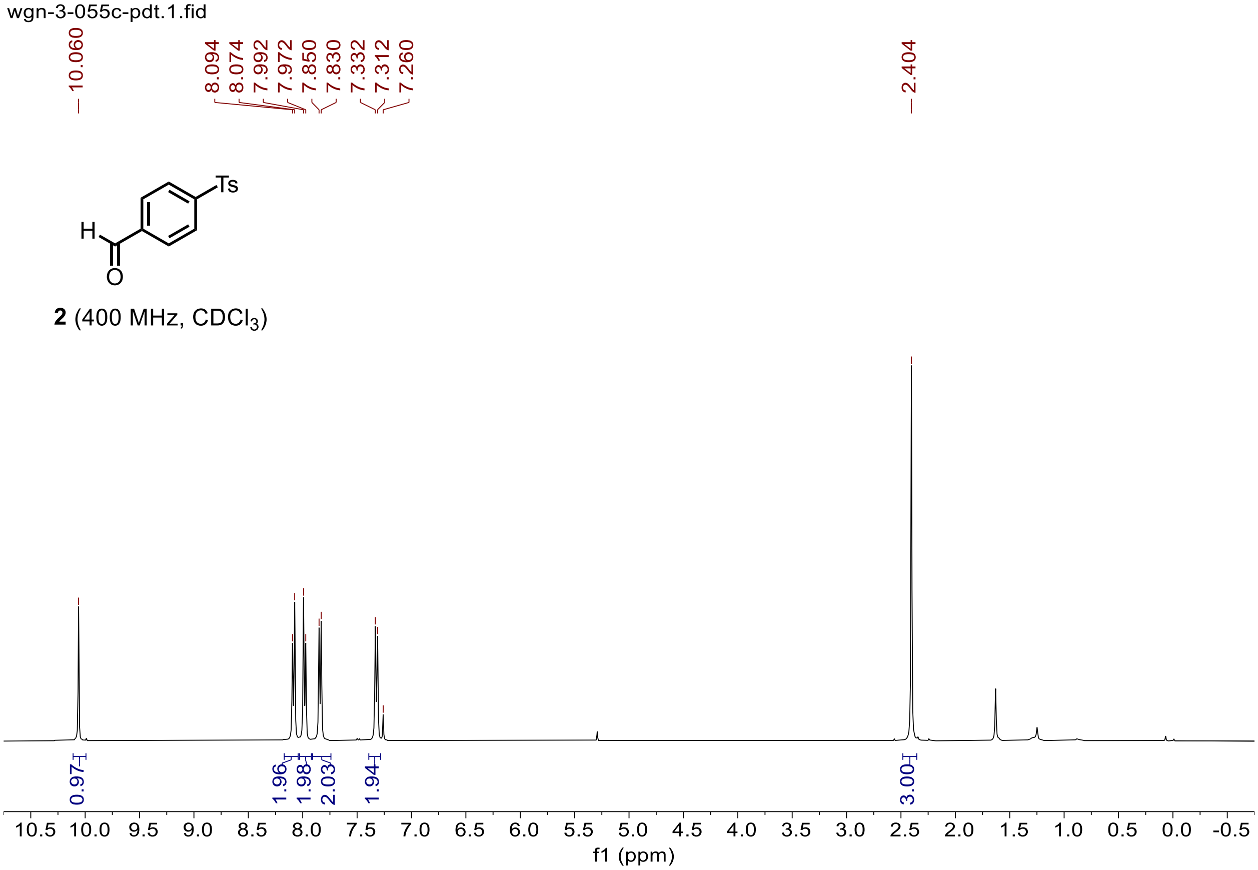
wgn-3-055c-pdt.1.fid
10.060
8.094
8.074
7.992
7.972
7.850
7.830
7.332
7.312
7.260
2.404
Ts
H
O
2 (400 MHz, CDCl3)
0.97
1.96
1.98
2.03
1.94
3.00
10.5 10.0 9.5 9.0 8.5 8.0 7.5 7.0 6.5 6.0 5.5 5.0 4.5 4.0 3.5 3.0 2.5 2.0 1.5 1.0 0.5 0.0 -0.5
f1 (ppm)

wgn-3-055c-pdt-C.1.fid

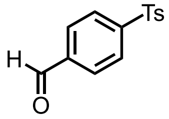

2 (100 MHz, $CDCl_3$)

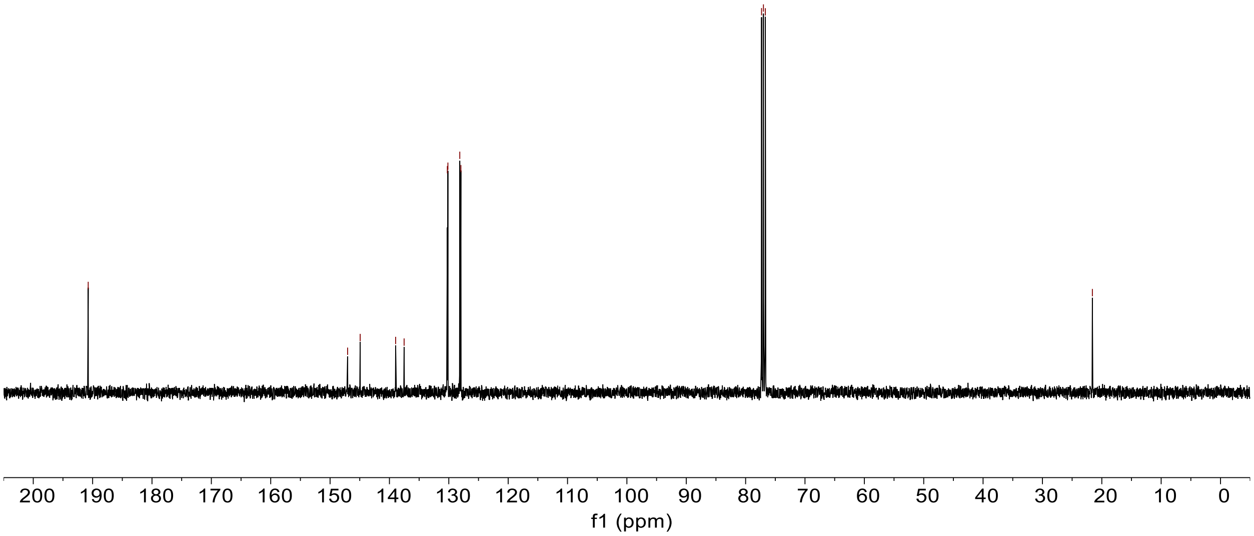

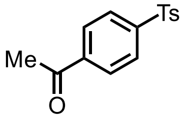

3 (400 MHz, $CDCl_3$)

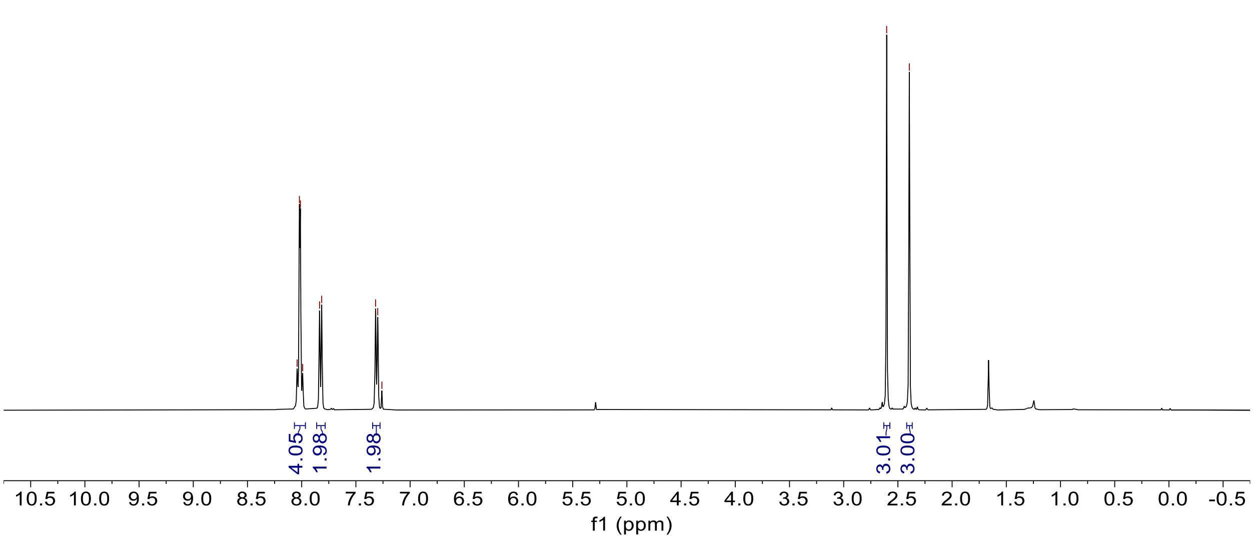

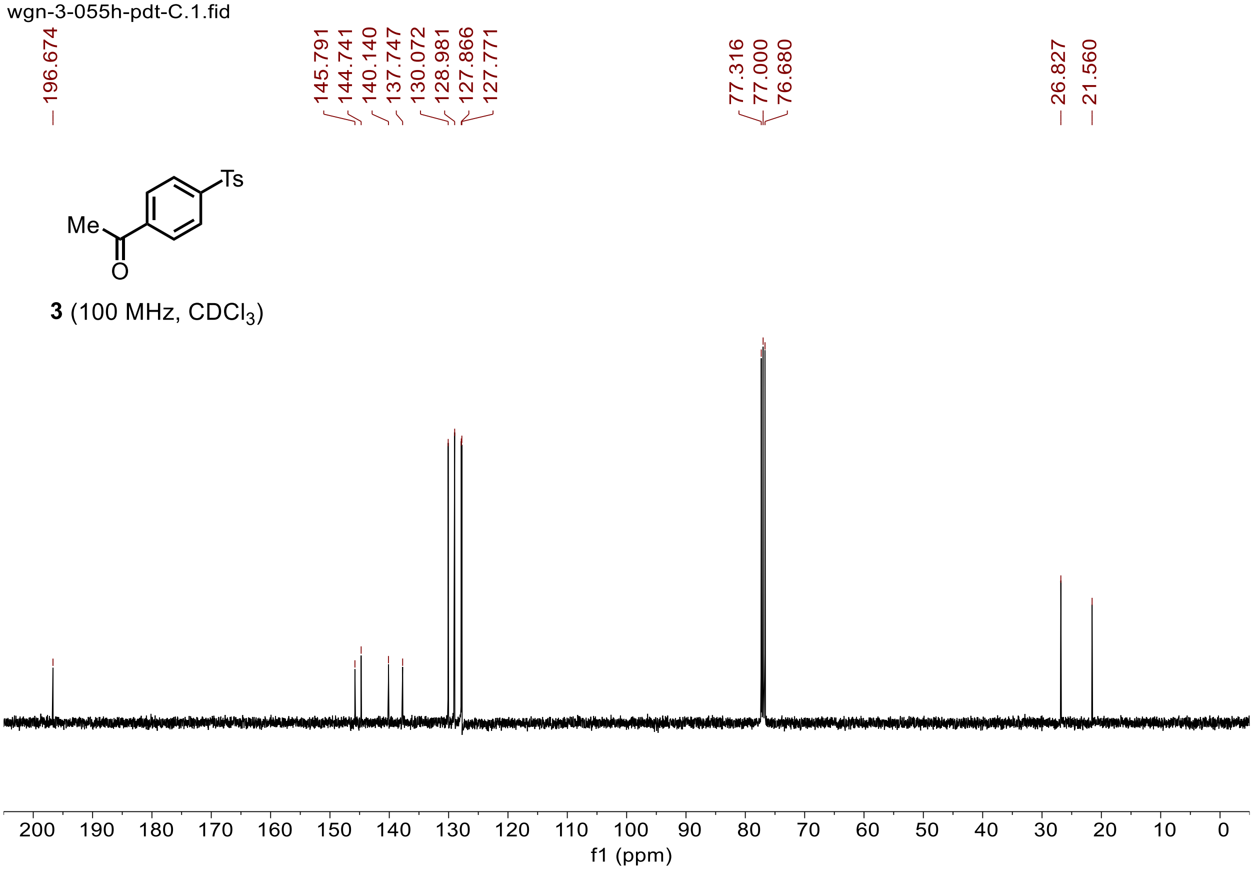
wgn-3-055h-pdt-C.1.fid
196.674
145.791
144.741
140.140
137.747
130.072
128.981
127.866
127.771
77.316
77.000
76.680
26.827
21.560
Me
Ts
O
3 (100 MHz, CDCl3)
200 190 180 170 160 150 140 130 120 110 100 90 80 70 60 50 40 30 20 10 0
f1 (ppm)

wgn-3-055q-pdt.3.fid

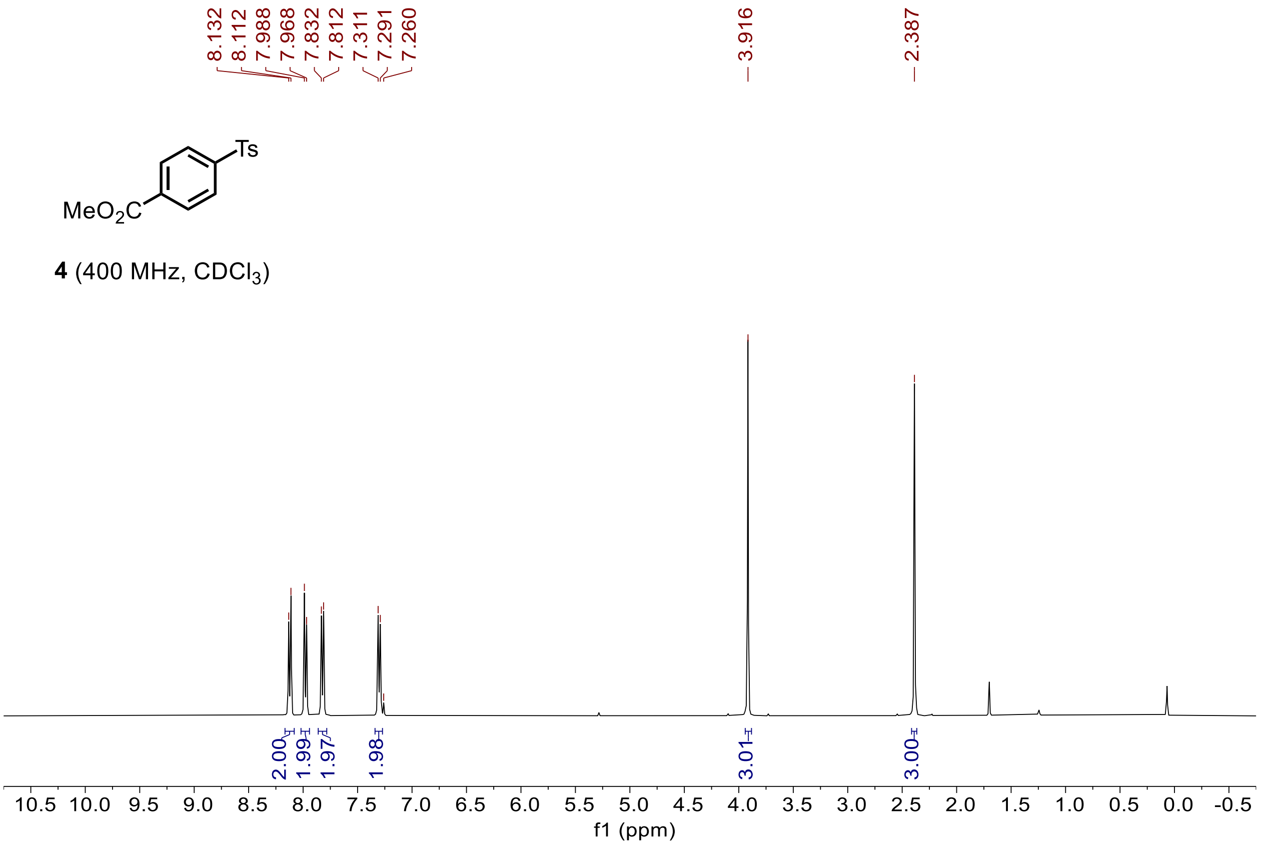

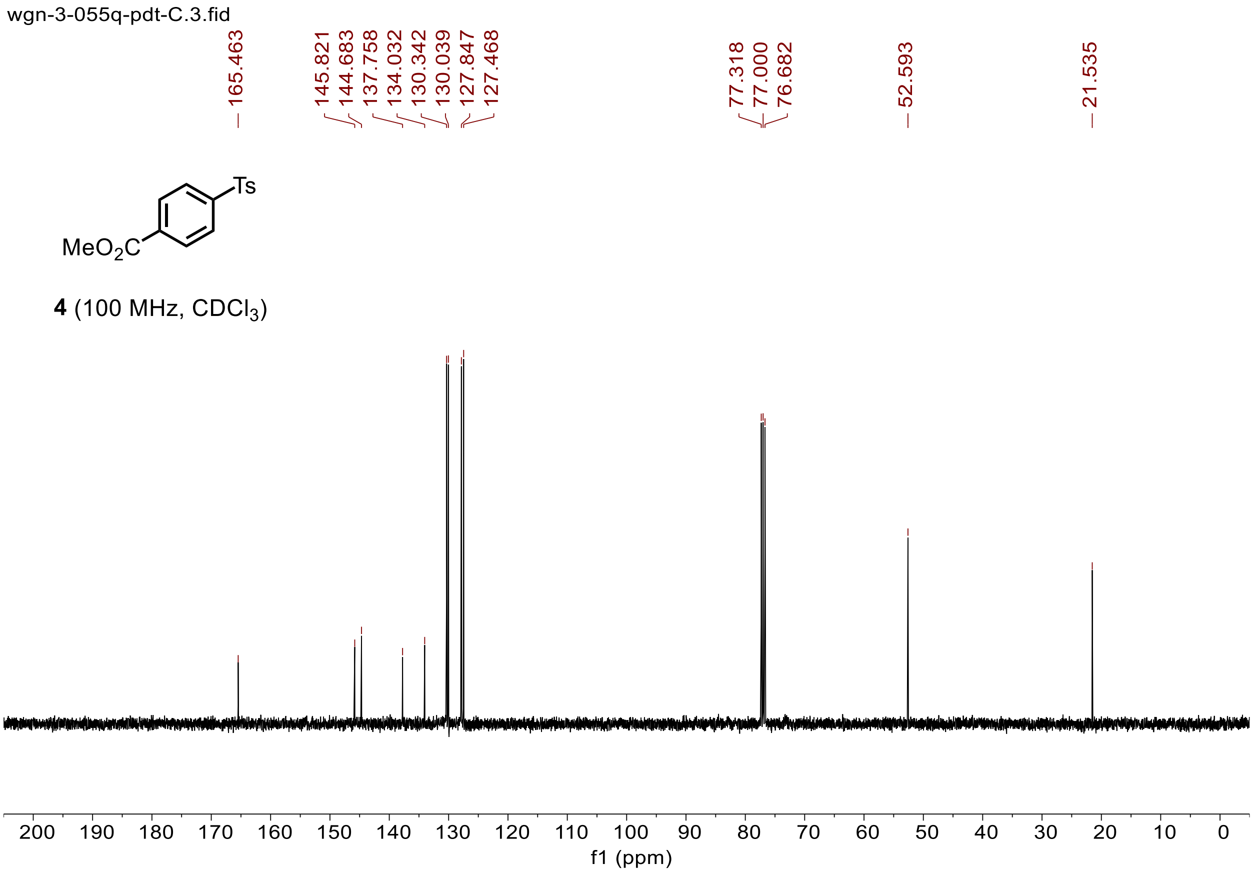
wgn-3-055q-pdt-C.3.fid
165.463
145.821
144.683
137.758
134.032
130.342
130.039
127.847
127.468
77.318
77.000
76.682
52.593
21.535
Ts
MeO_2C
4 (100 MHz, $CDCl_3$)
200 190 180 170 160 150 140 130 120 110 100 90 80 70 60 50 40 30 20 10 0
f1 (ppm)

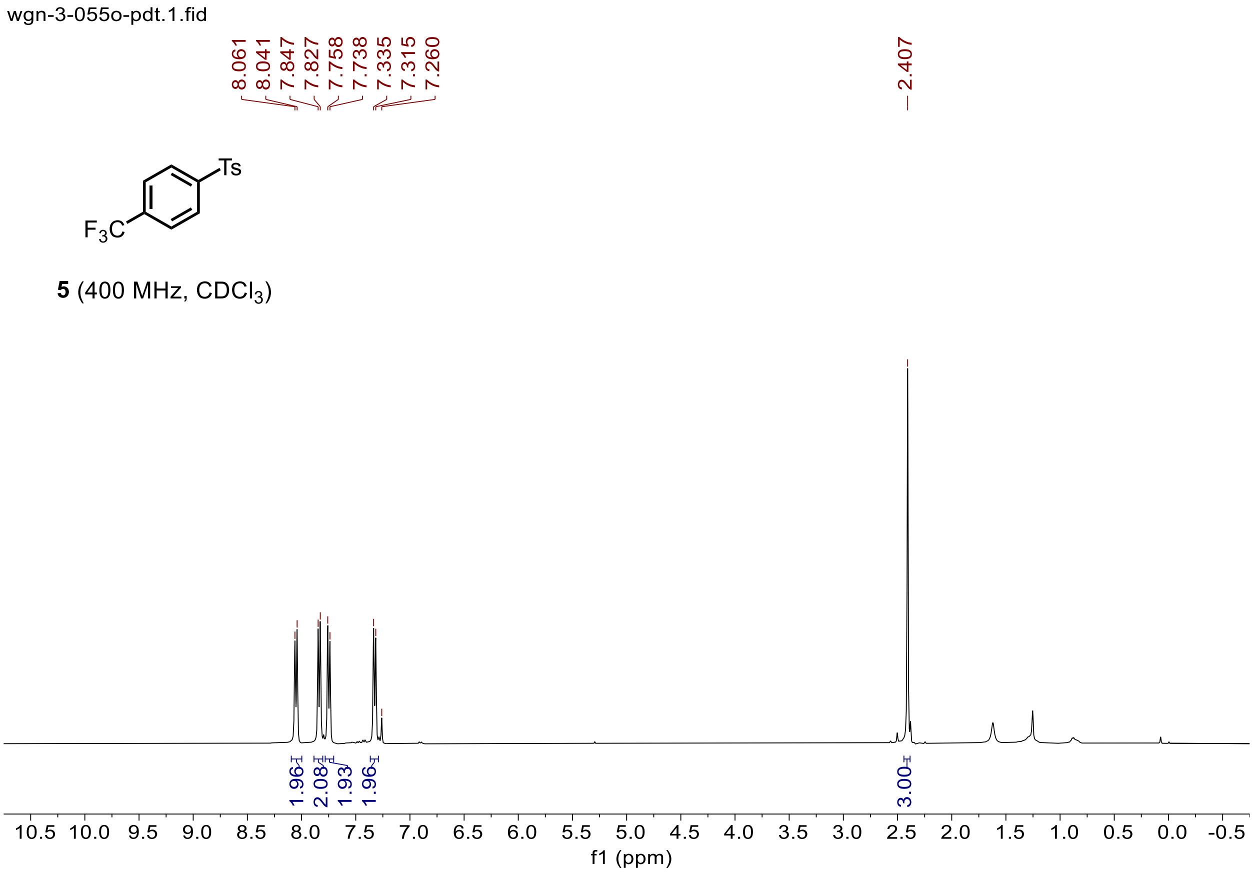
wgn-3-055o-pdt.1.fid
8.061
8.041
7.847
7.827
7.758
7.738
7.335
7.315
7.260
2.407
Ts
F3C
5 (400 MHz, CDCl3)
1.96
2.08
1.93
1.96
3.00
10.5 10.0 9.5 9.0 8.5 8.0 7.5 7.0 6.5 6.0 5.5 5.0 4.5 4.0 3.5 3.0 2.5 2.0 1.5 1.0 0.5 0.0 -0.5
f1 (ppm)

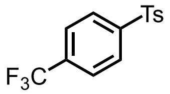

5 (100 MHz, $CDCl_3$)

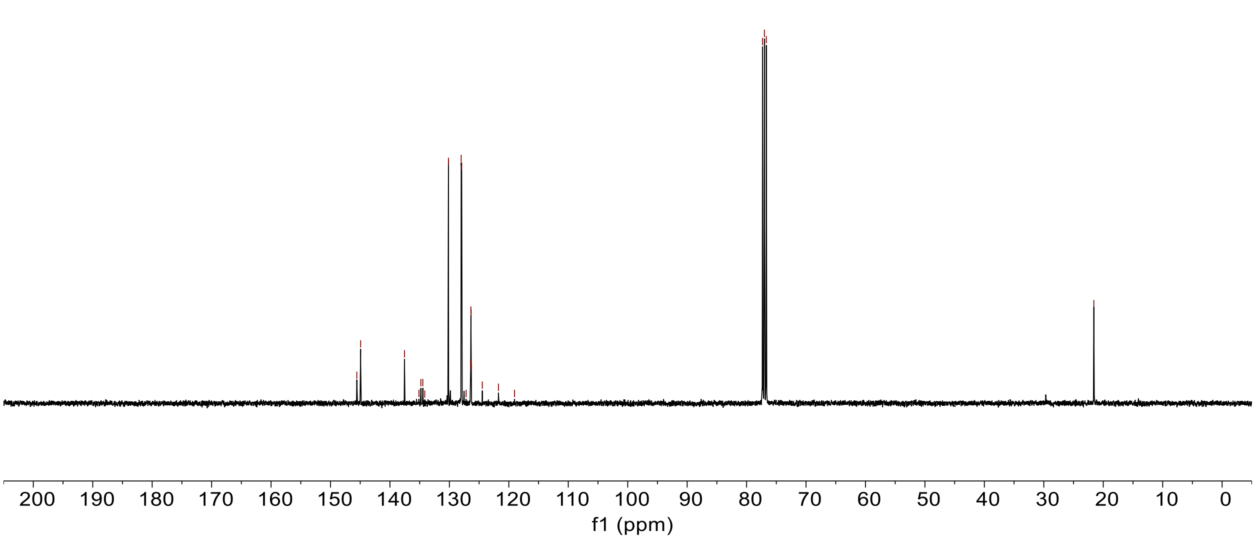

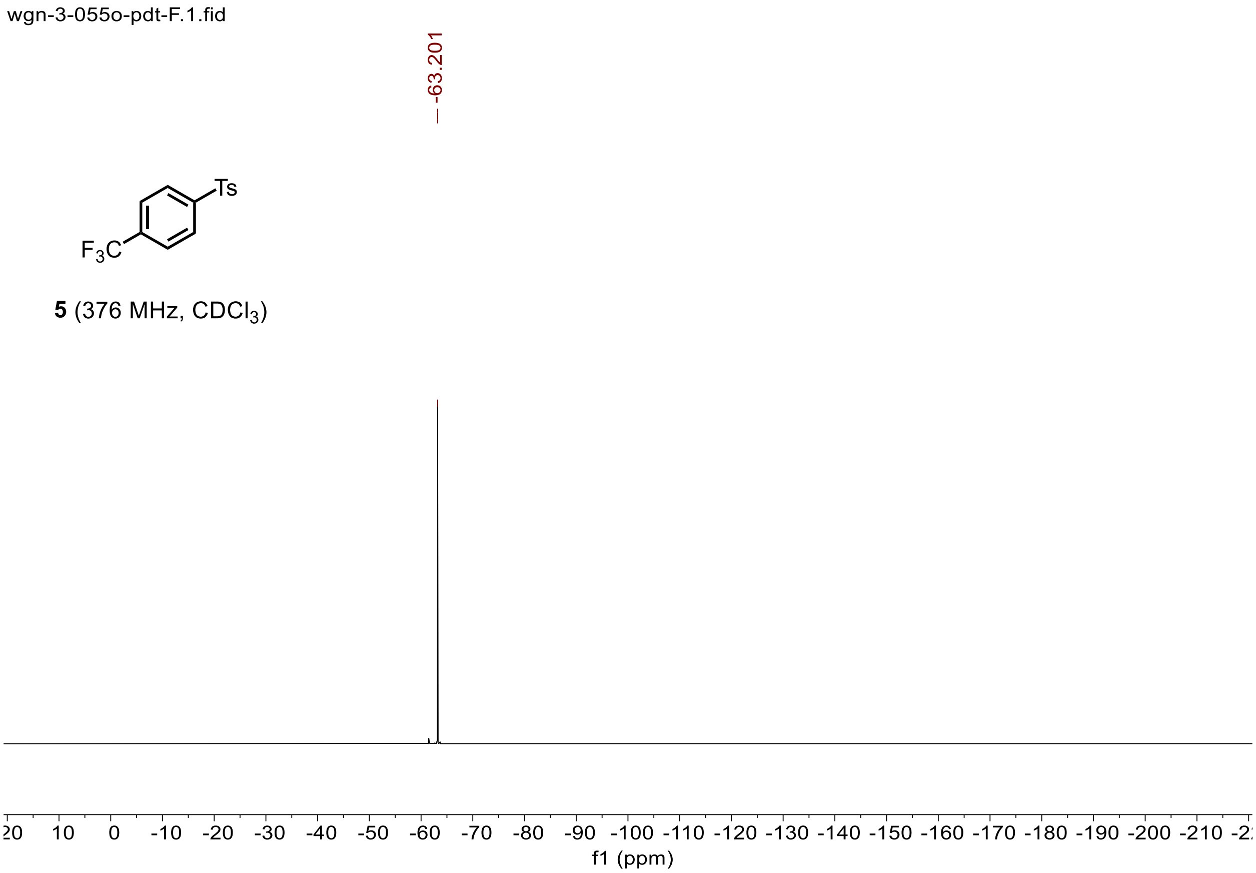
wgn-3-055o-pdt-F.1.fid
-63.201
Ts
F3C
5 (376 MHz, CDCl3)
20 10 0 -10 -20 -30 -40 -50 -60 -70 -80 -90 -100 -110 -120 -130 -140 -150 -160 -170 -180 -190 -200 -210
f1 (ppm)

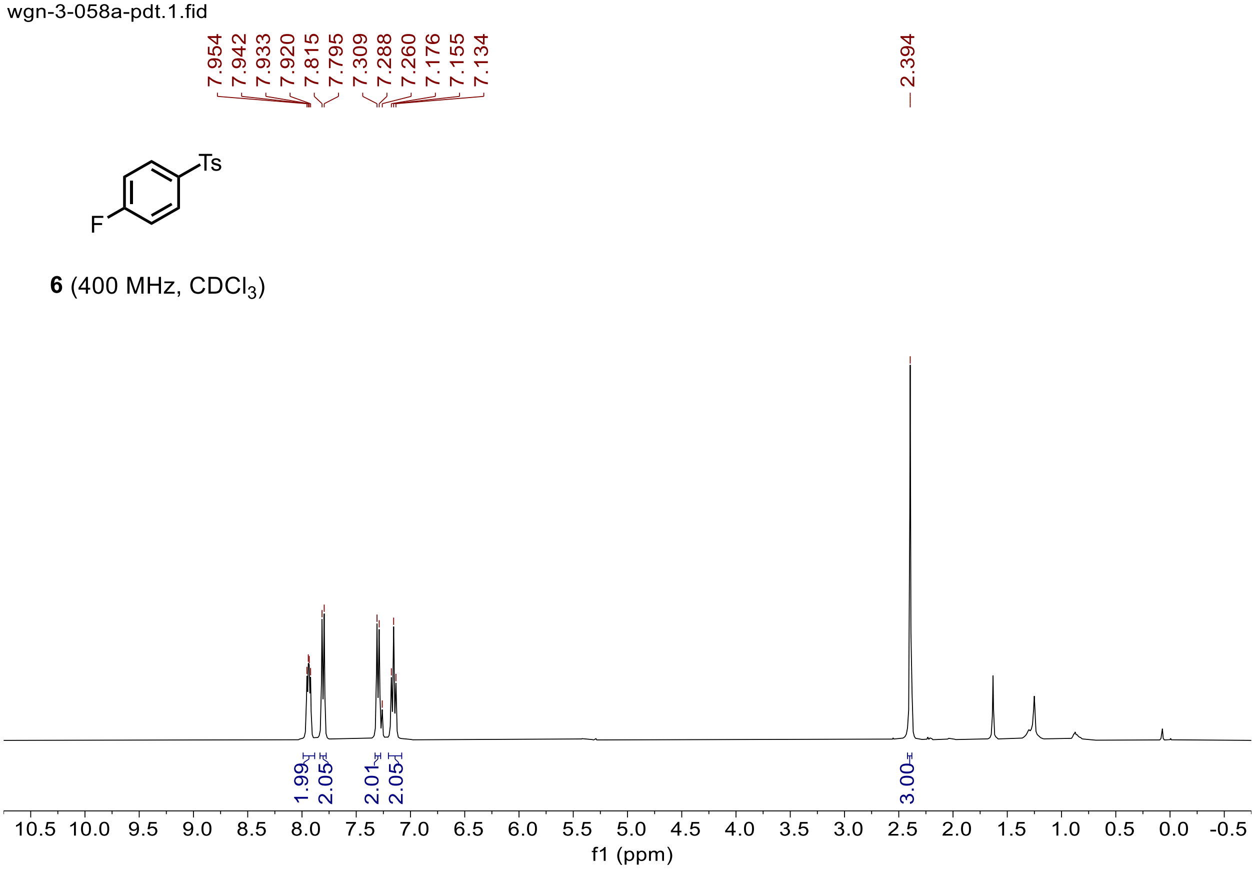

6 (400 MHz, $CDCl_3$)

wgn-3-058a-pdt-C.1.fid

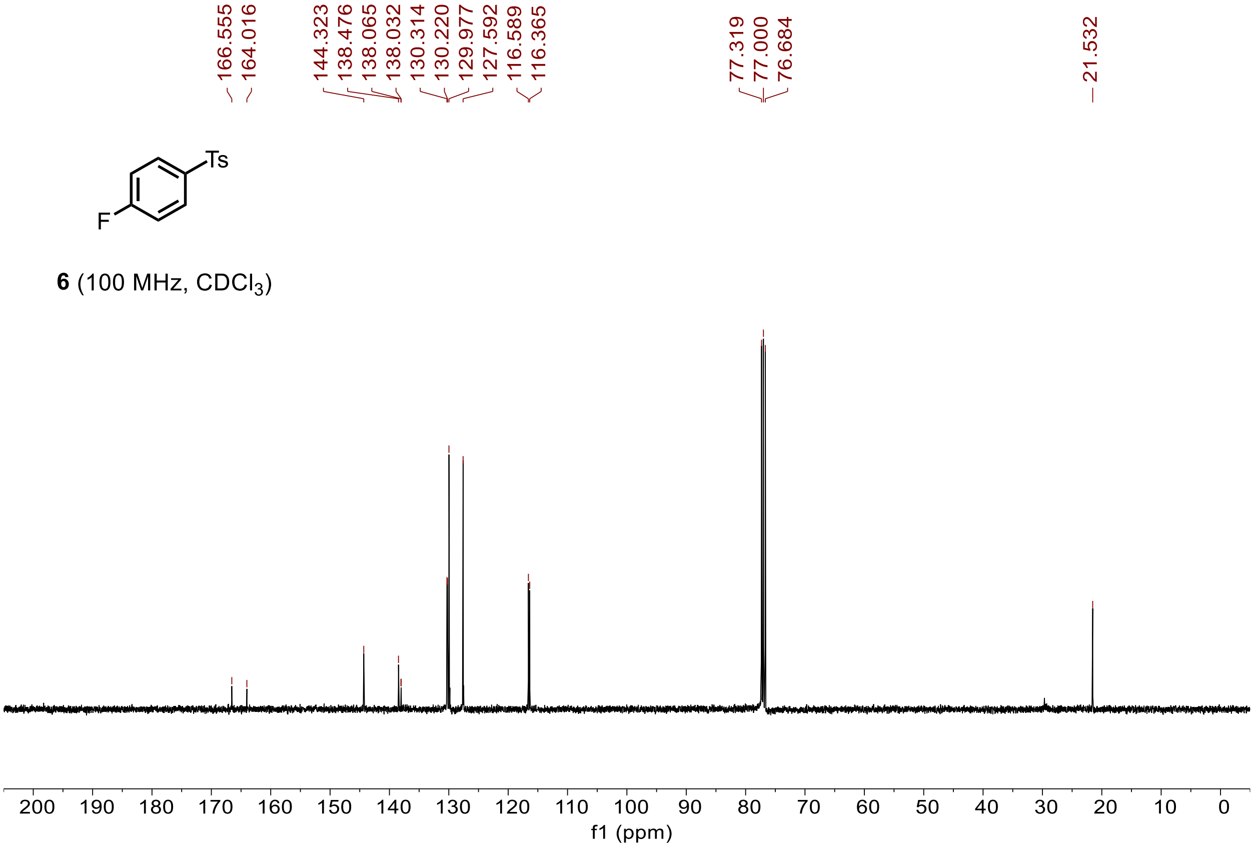

6 (100 MHz, $CDCl_3$)

wgn-3-058a-pdt-F.1.fid

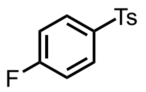

6 (376 MHz, $CDCl_3$)

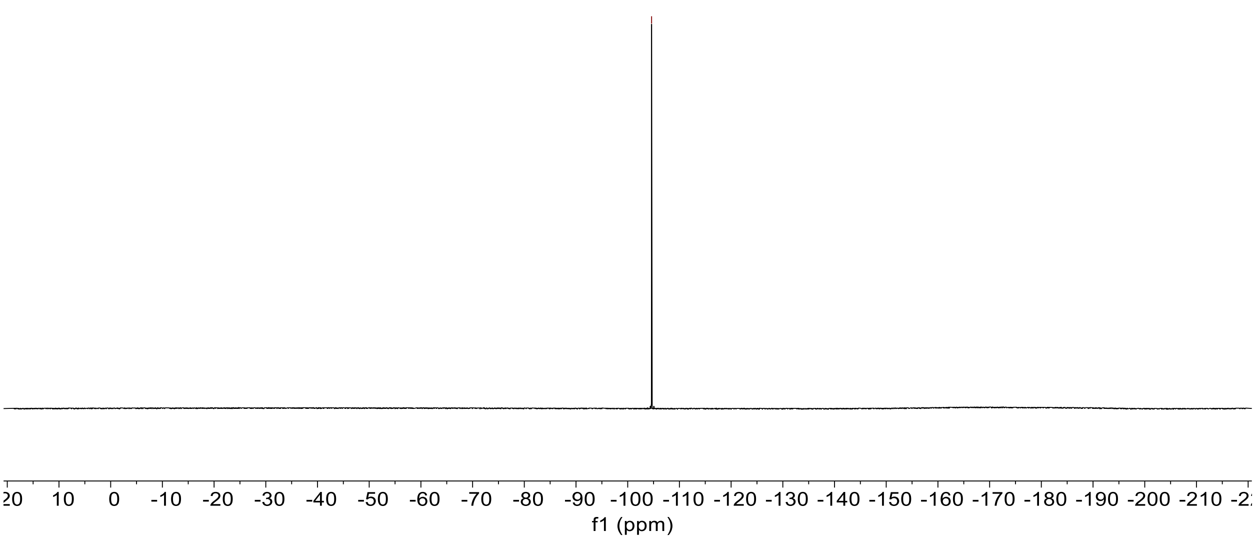

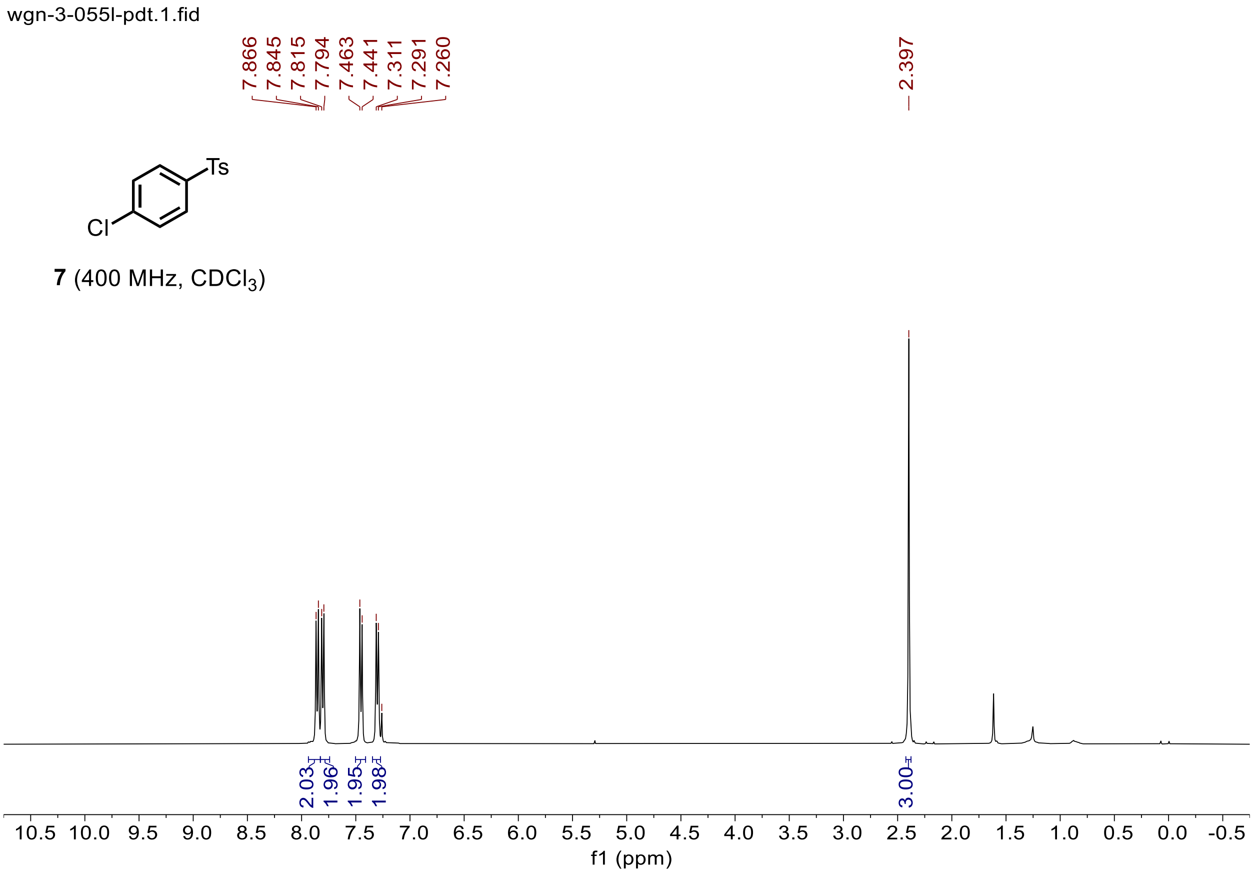
wgn-3-055l-pdt.1.fid
7.866
7.845
7.815
7.794
7.463
7.441
7.311
7.291
7.260
2.397
Ts
Cl
7 (400 MHz, CDCl3)
2.03
1.96
1.95
1.98
3.00
10.5 10.0 9.5 9.0 8.5 8.0 7.5 7.0 6.5 6.0 5.5 5.0 4.5 4.0 3.5 3.0 2.5 2.0 1.5 1.0 0.5 0.0 -0.5
f1 (ppm)

wgn-3-055l-pdt-C.1.fid

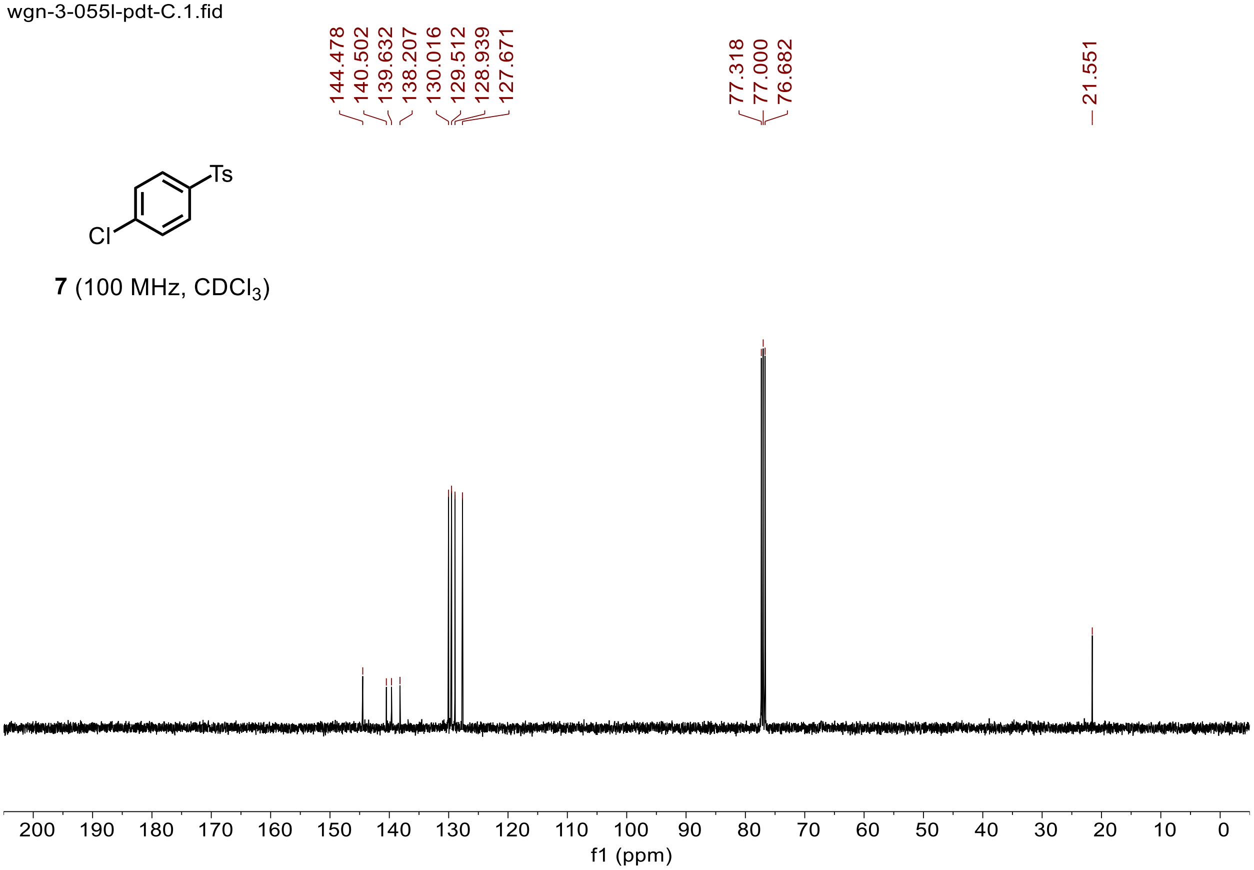

7 (100 MHz, $CDCl_3$)

wgn-3-055n-d.1.fid

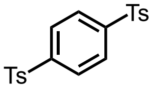

8 (400 MHz, $CDCl_3$)

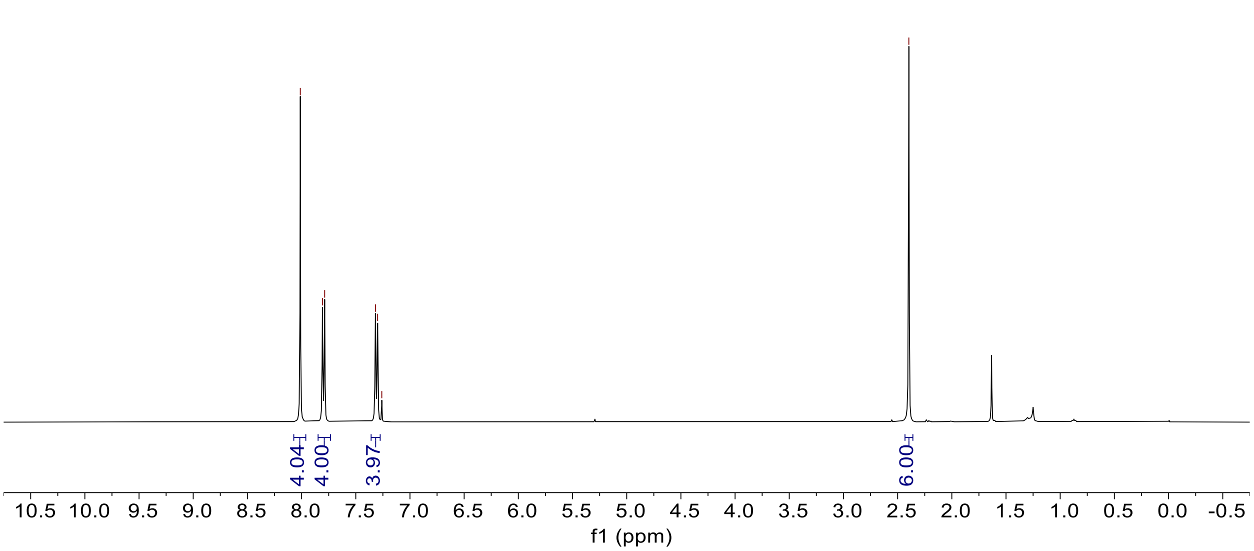

wgn-3-055n-d-C.1.fid

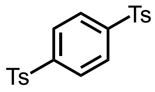

8 (100 MHz, $CDCl_3$)

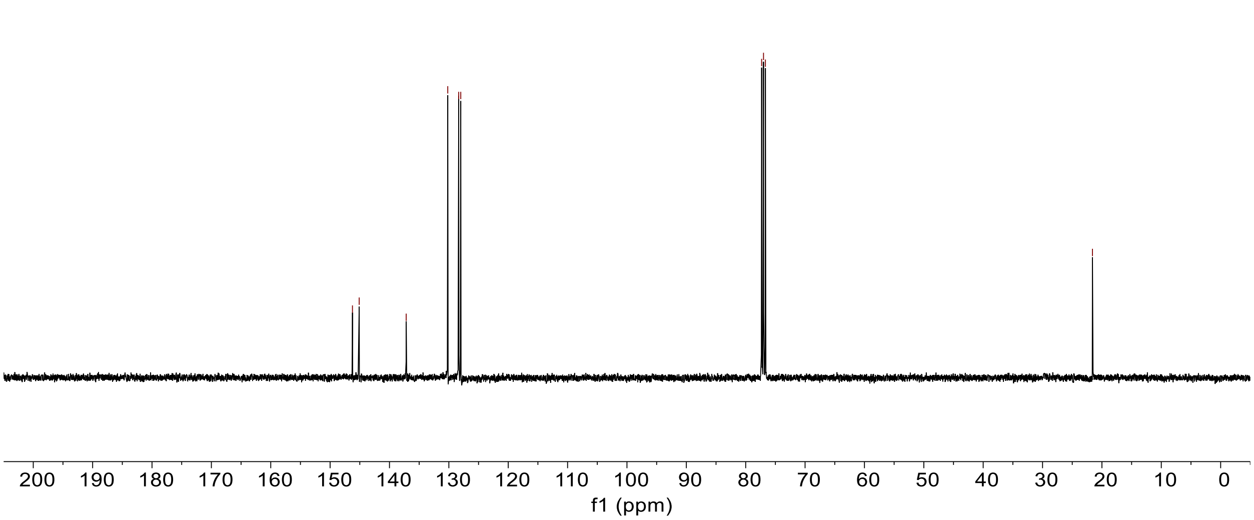

wgn-3-058b-pdt.1.fid

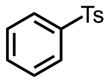

9 (400 MHz, $CDCl_3$)

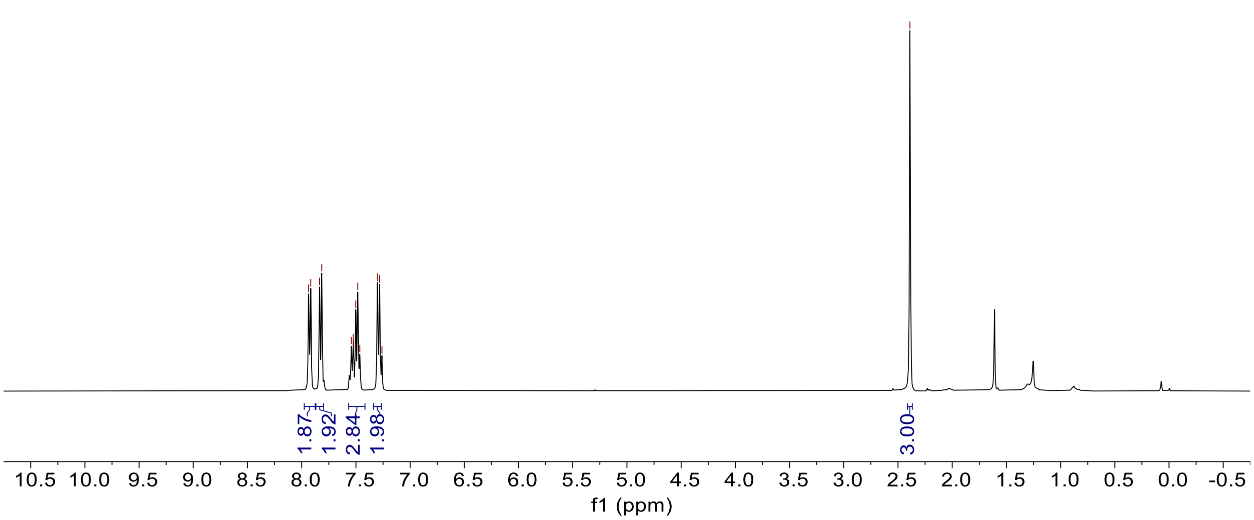

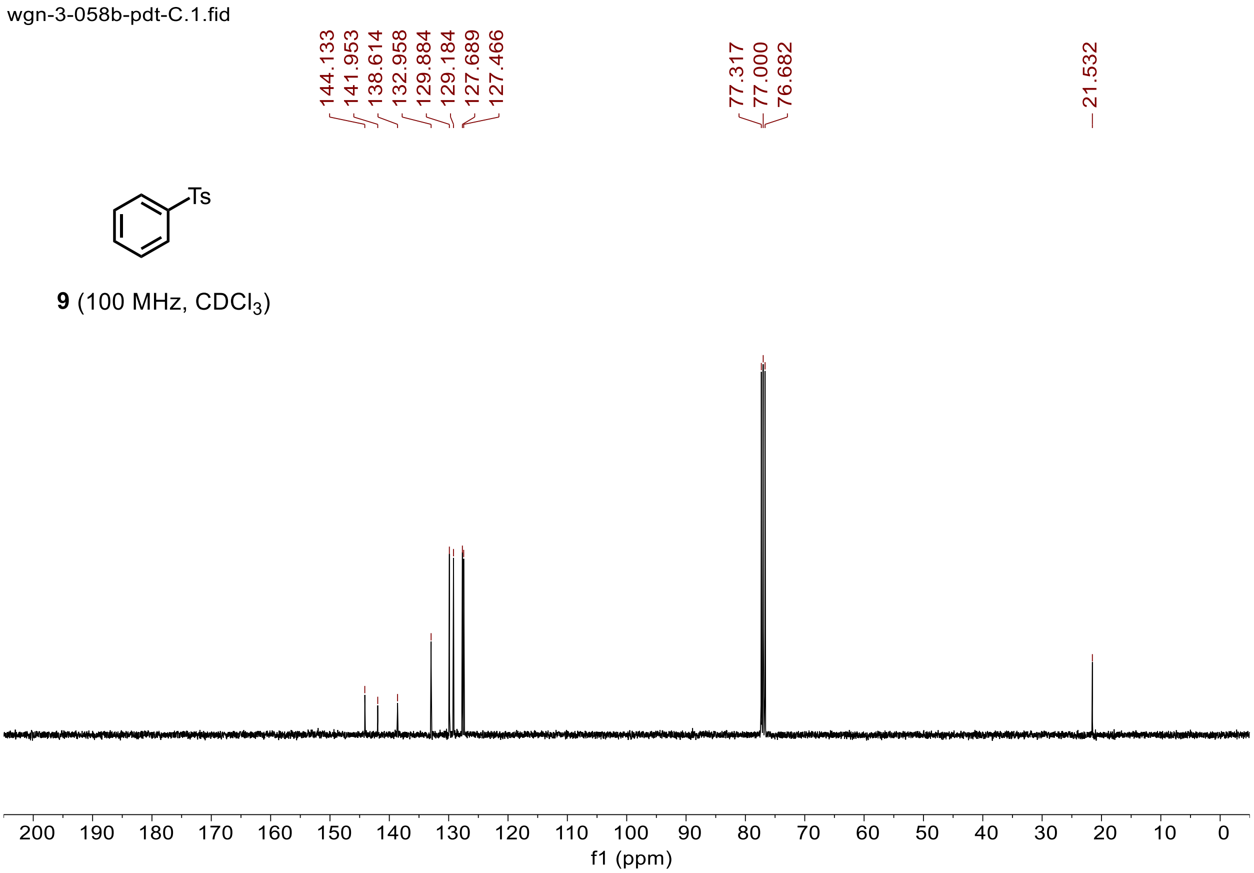
wgn-3-058b-pdt-C.1.fid
144.133
141.953
138.614
132.958
129.884
129.184
127.689
127.466
77.317
77.000
76.682
21.532
Ts
9 (100 MHz, CDCl3)
200 190 180 170 160 150 140 130 120 110 100 90 80 70 60 50 40 30 20 10 0
f1 (ppm)

wgn-3-055g-pdt.1.fid

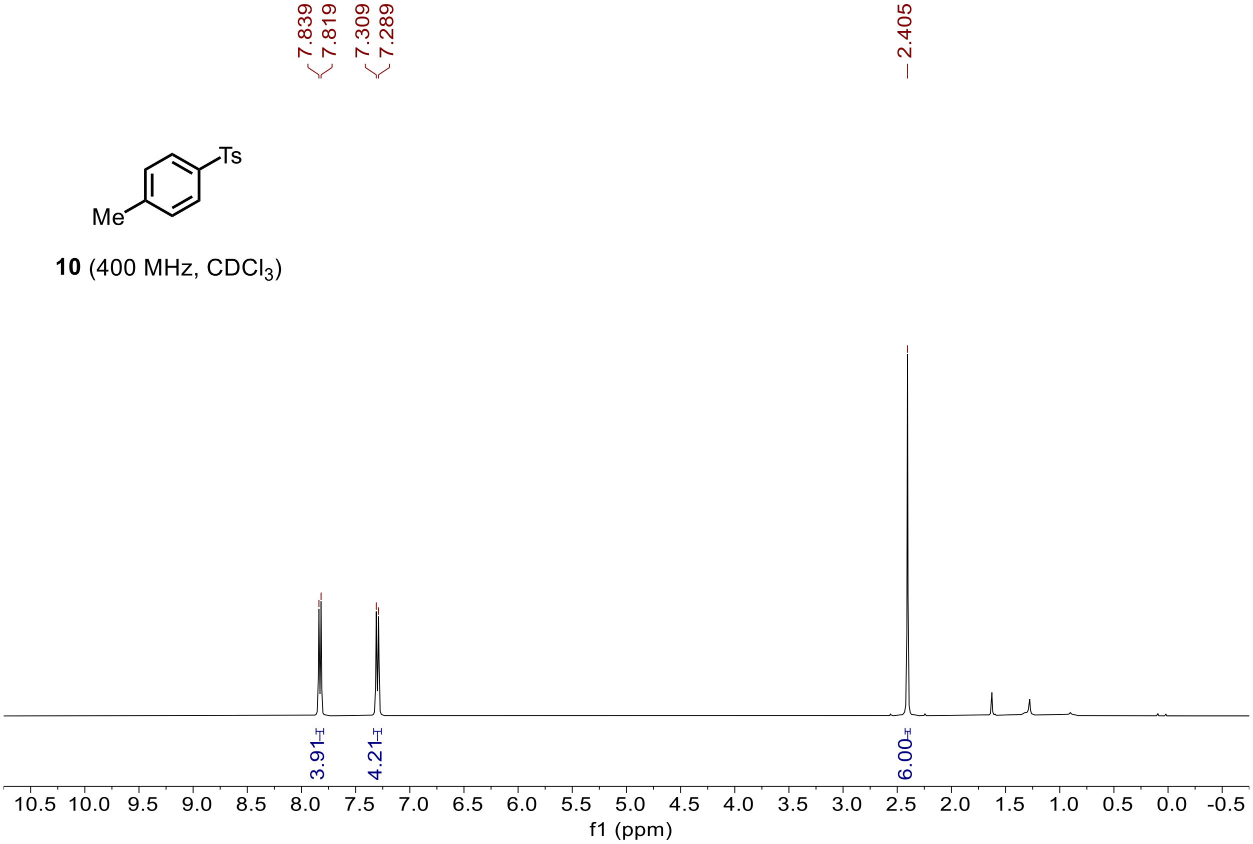

10 (400 MHz, $CDCl_3$)

wgn-3-055g-pdt-C.1.fid

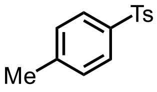

10 (100 MHz, $CDCl_3$)

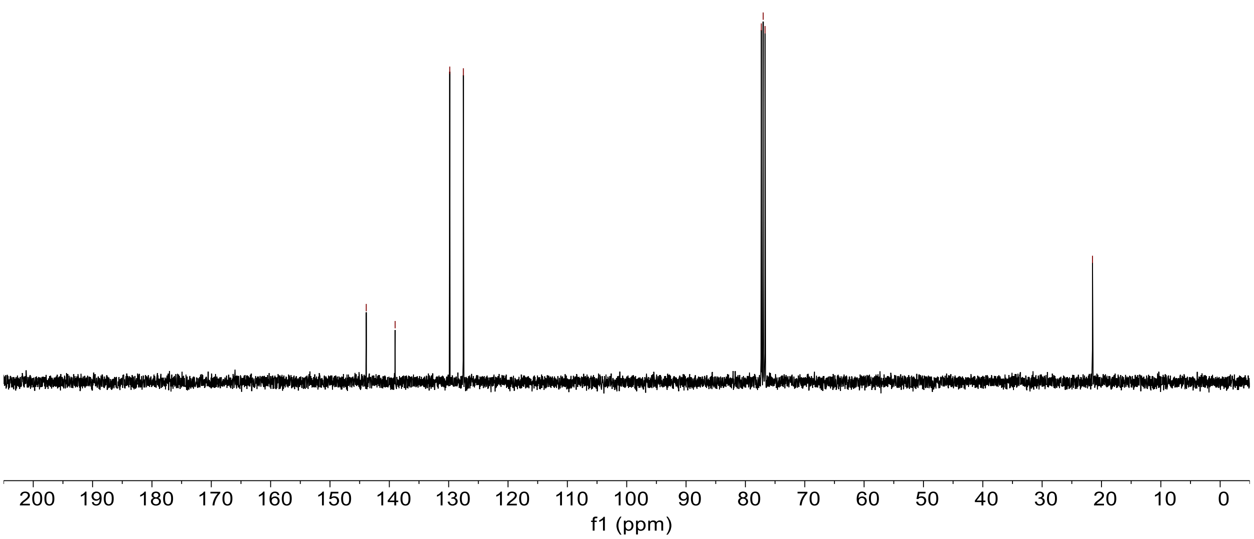

wgn-3-058k-pdt.1.fid

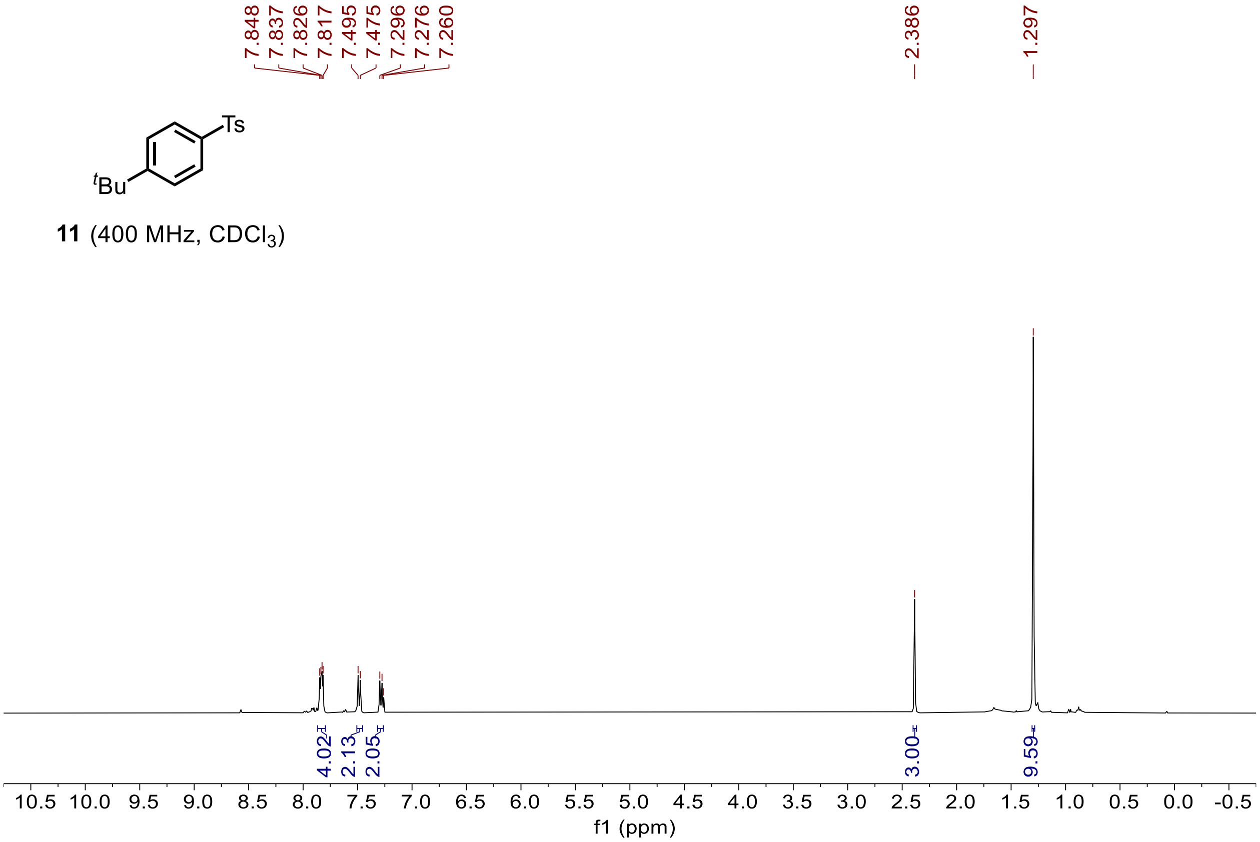

11 (400 MHz, $CDCl_3$)

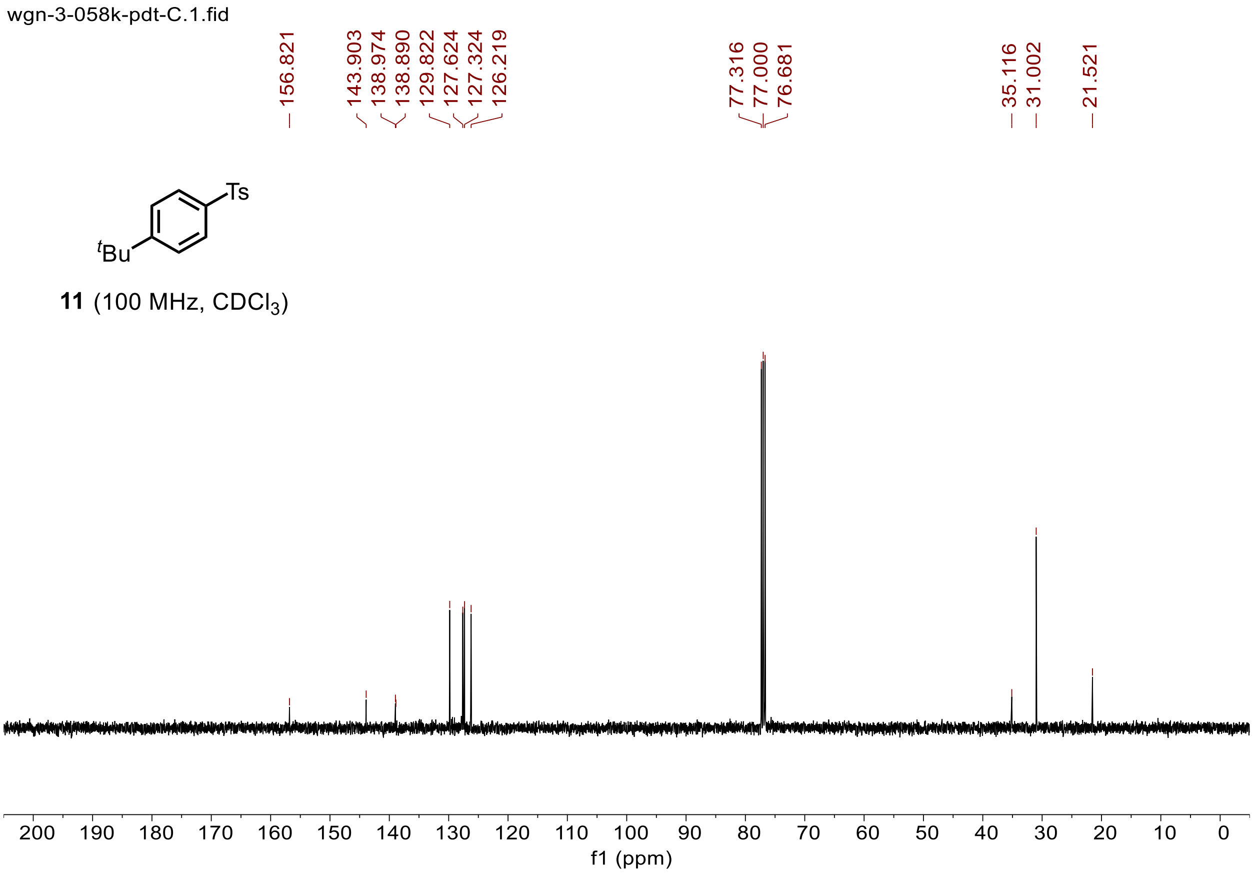

11 (100 MHz, $CDCl_3$)

wgn-3-055d-pdt.1.fid

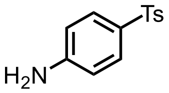

12 (400 MHz, $CDCl_3$)

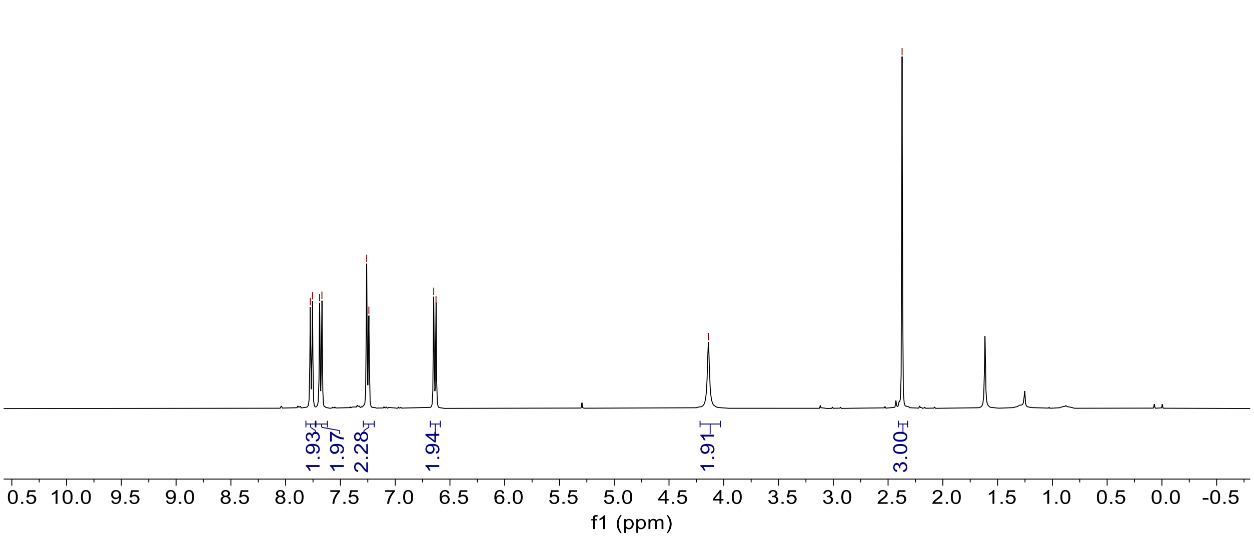

wgn-3-055d-pdt-C.1.fid

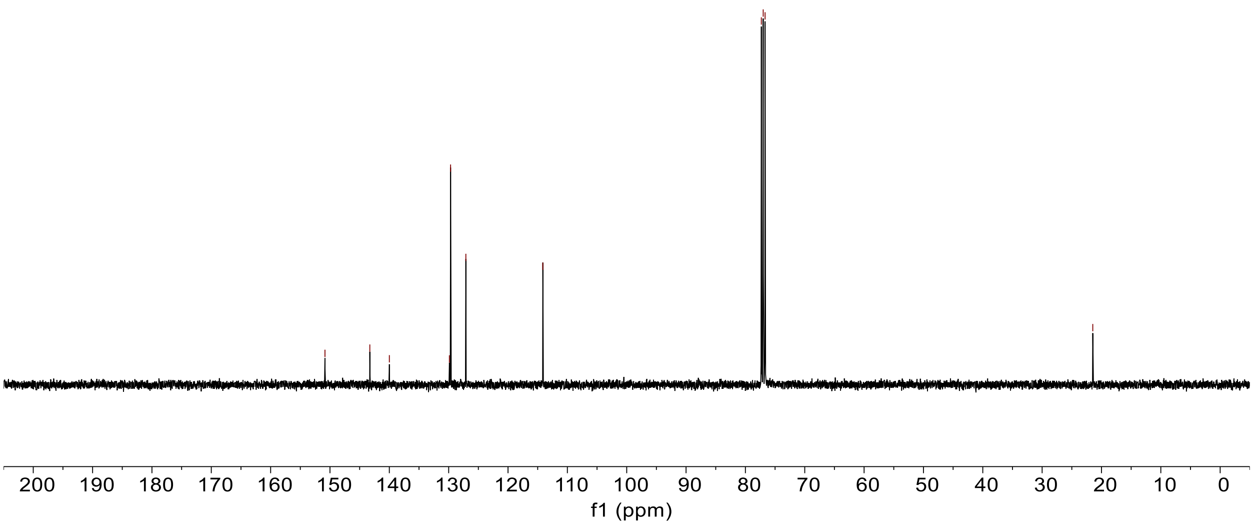

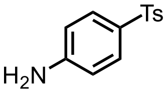

12 (100 MHz, $CDCl_3$)

wgn-3-055e-pdt.7.fid

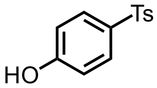

13 (400 MHz, $CDCl_3$)

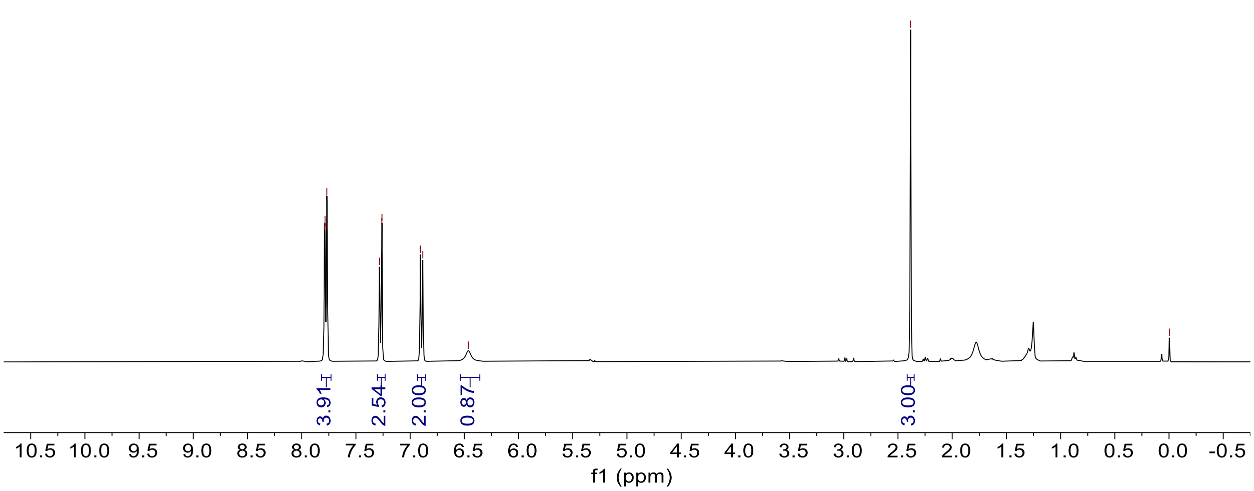

wgn-3-055e-pdt-C.4.fid

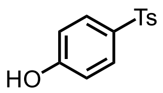

13 (100 MHz, $CDCl_3$)

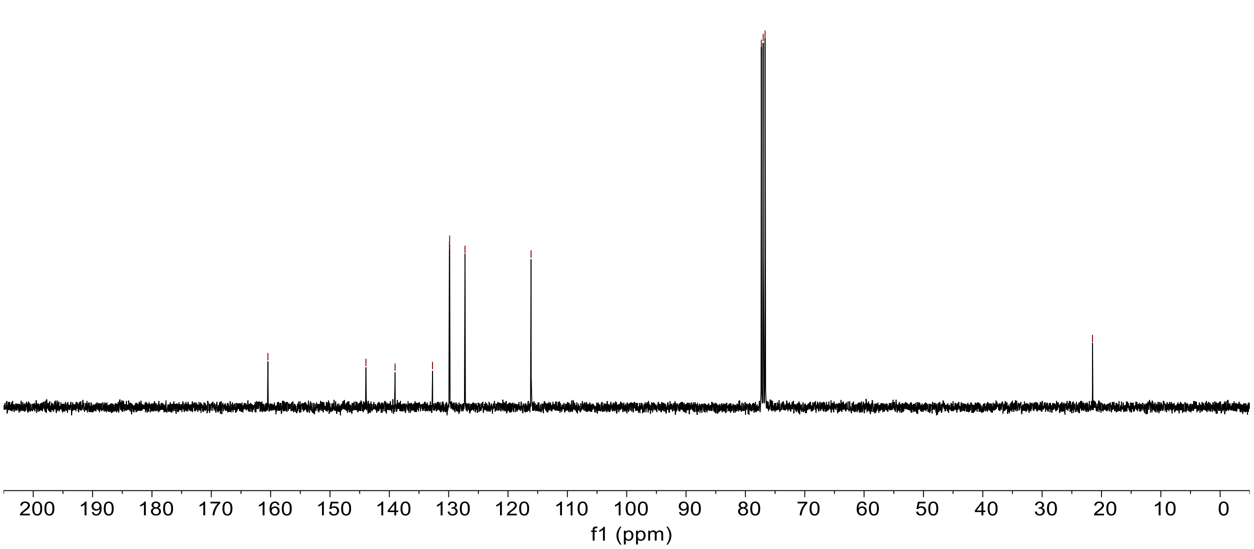

wgn-3-055j-pdt.1.fid

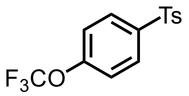

14 (400 MHz, $CDCl_3$)

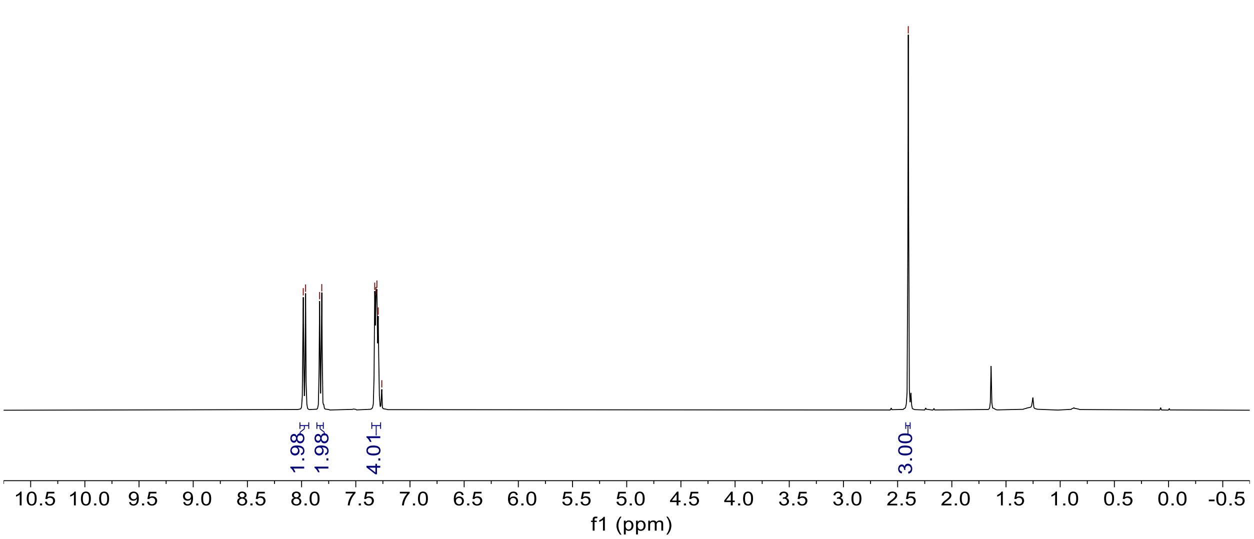

wgn-3-055j-pdt-C.1.fid

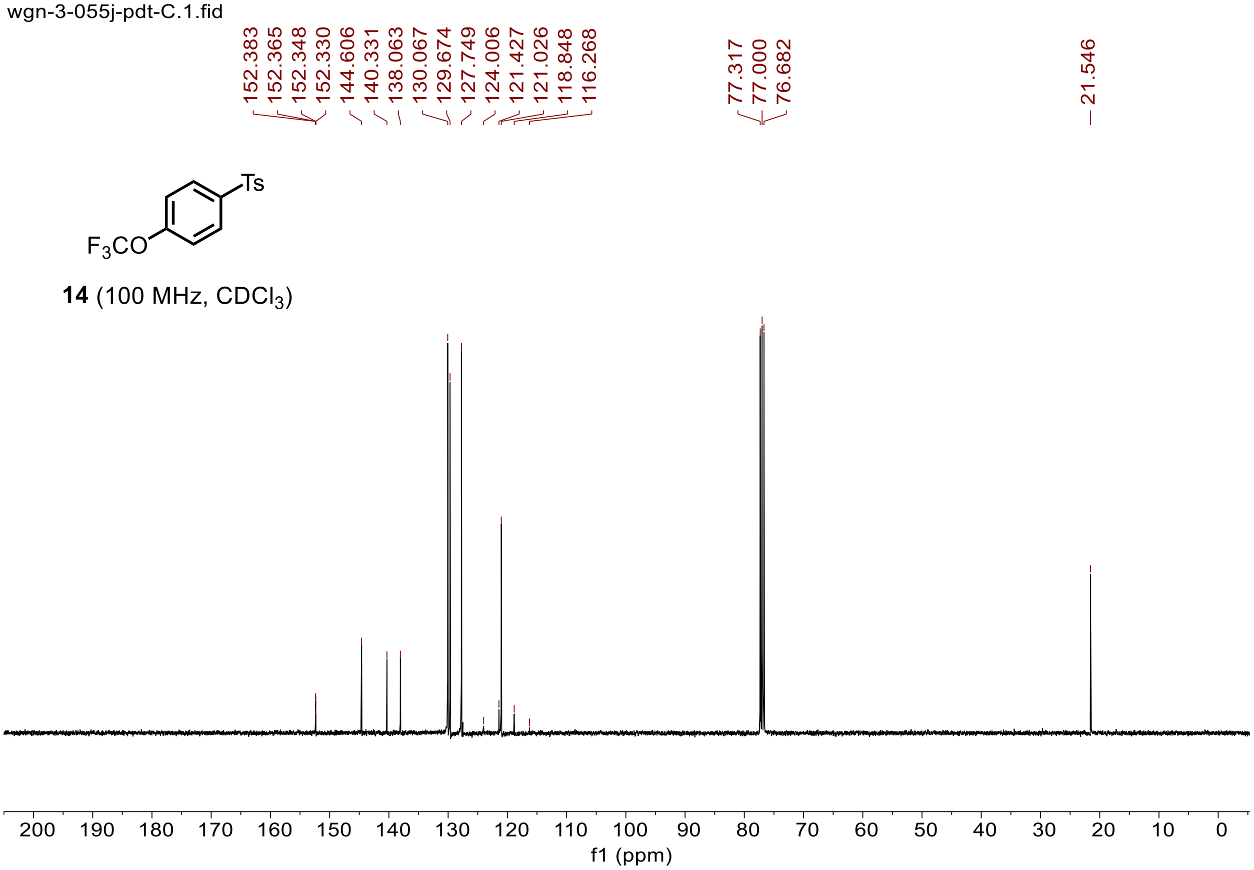

14 (100 MHz, $CDCl_3$)

wgn-3-055j-pdt-F.1.fid

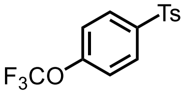

14 (376 MHz, $CDCl_3$)

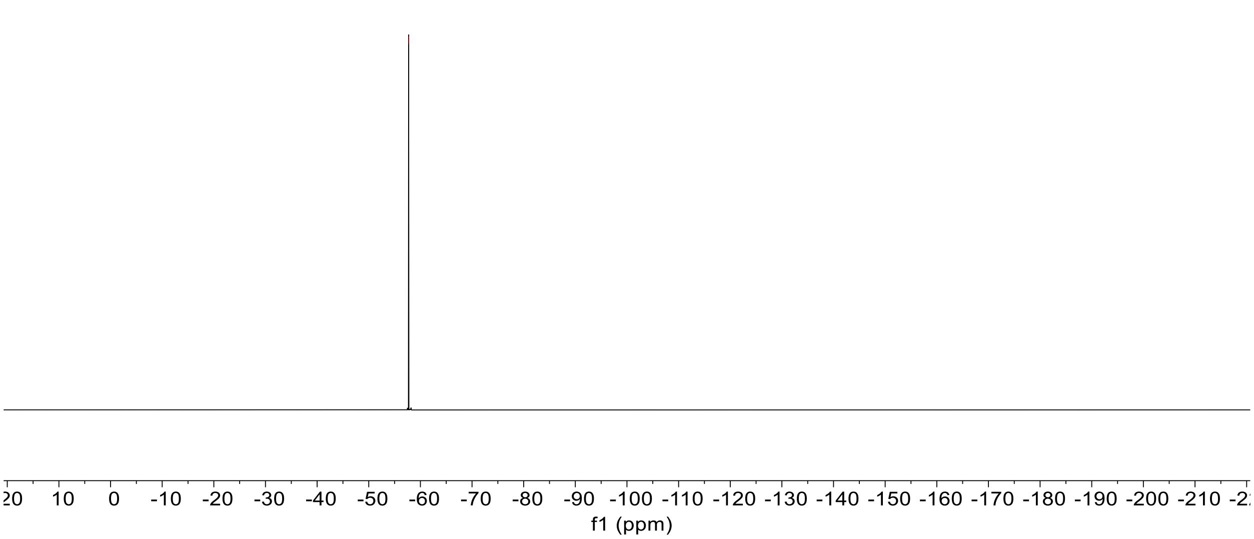

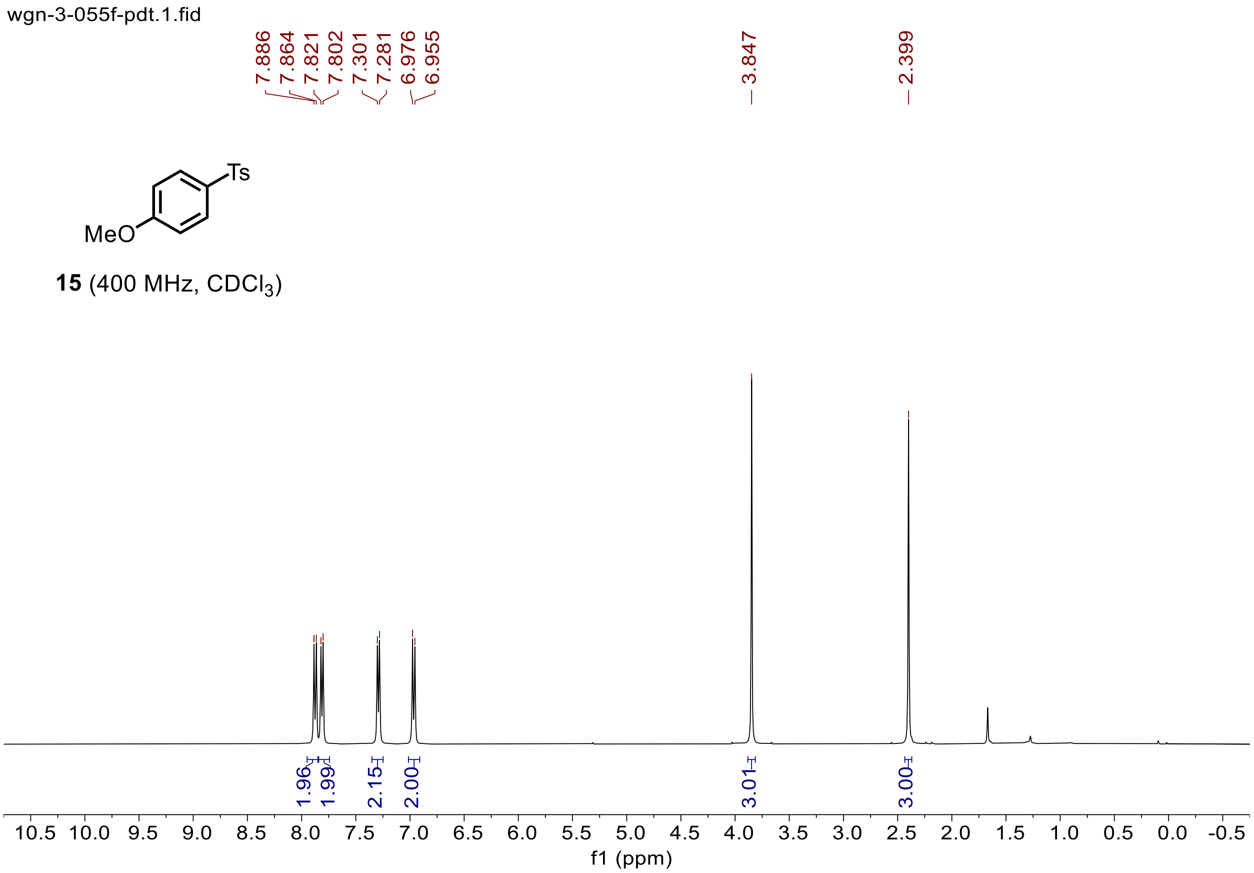
wgn-3-055f-pdt.1.fid
7.886
7.864
7.821
7.802
7.301
7.281
6.976
6.955
3.847
2.399
Ts
MeO
15 (400 MHz, CDCl3)
1.96
1.99
2.15
2.00
3.01
3.00
10.5 10.0 9.5 9.0 8.5 8.0 7.5 7.0 6.5 6.0 5.5 5.0 4.5 4.0 3.5 3.0 2.5 2.0 1.5 1.0 0.5 0.0 -0.5
f1 (ppm)

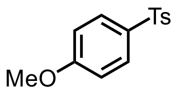

15 (100 MHz, $CDCl_3$)

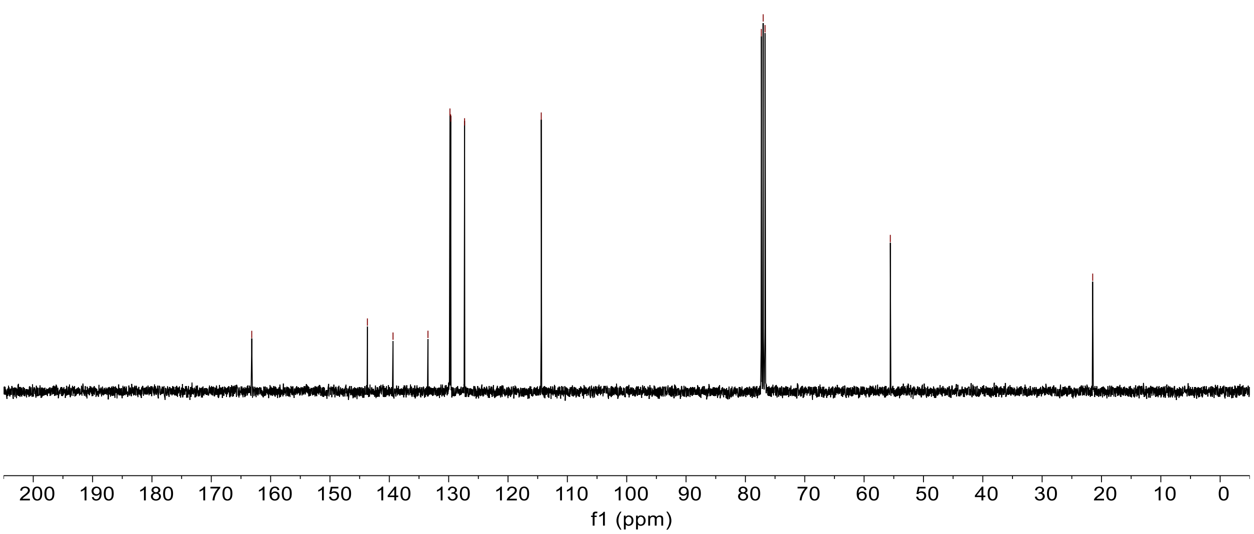

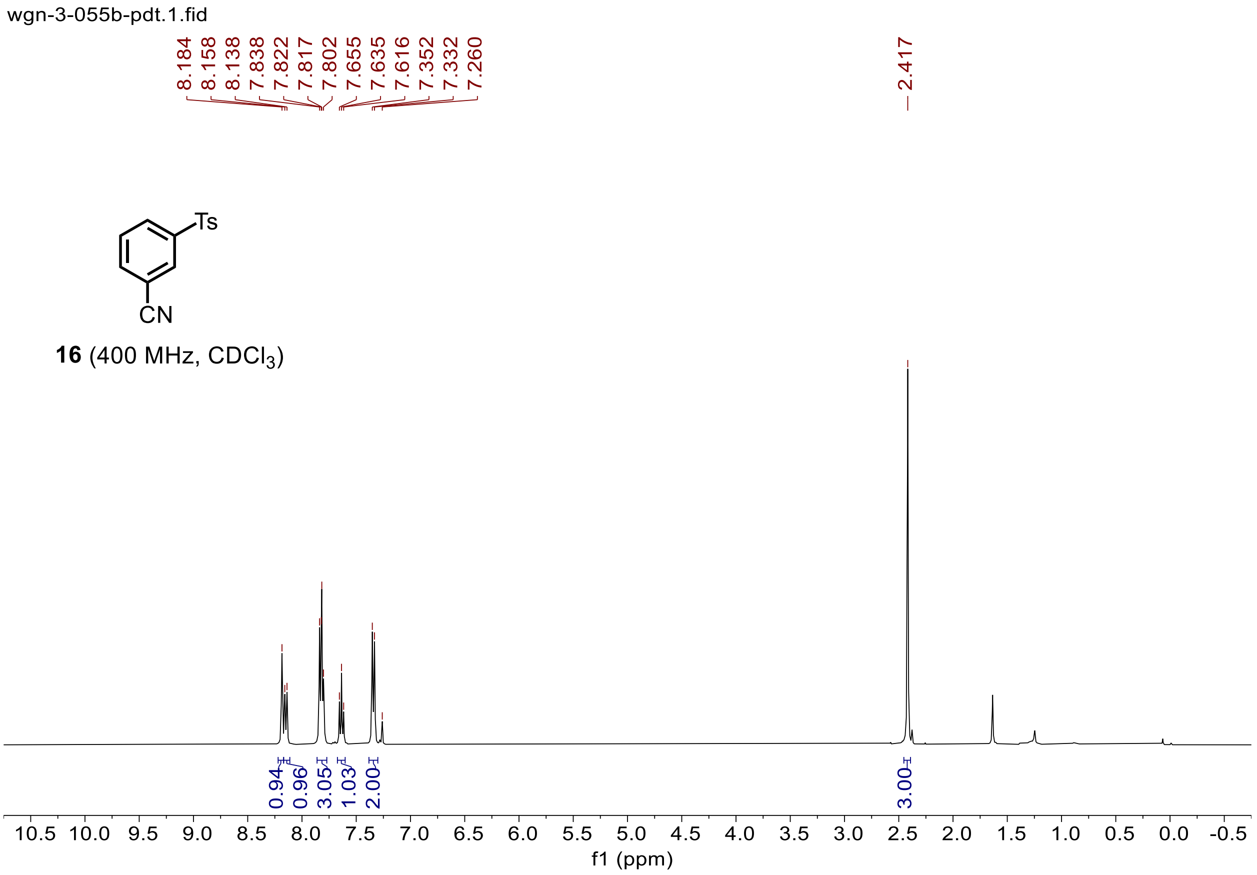

16 (400 MHz, $CDCl_3$)

wgn-3-055b-pdt-C.1.fid

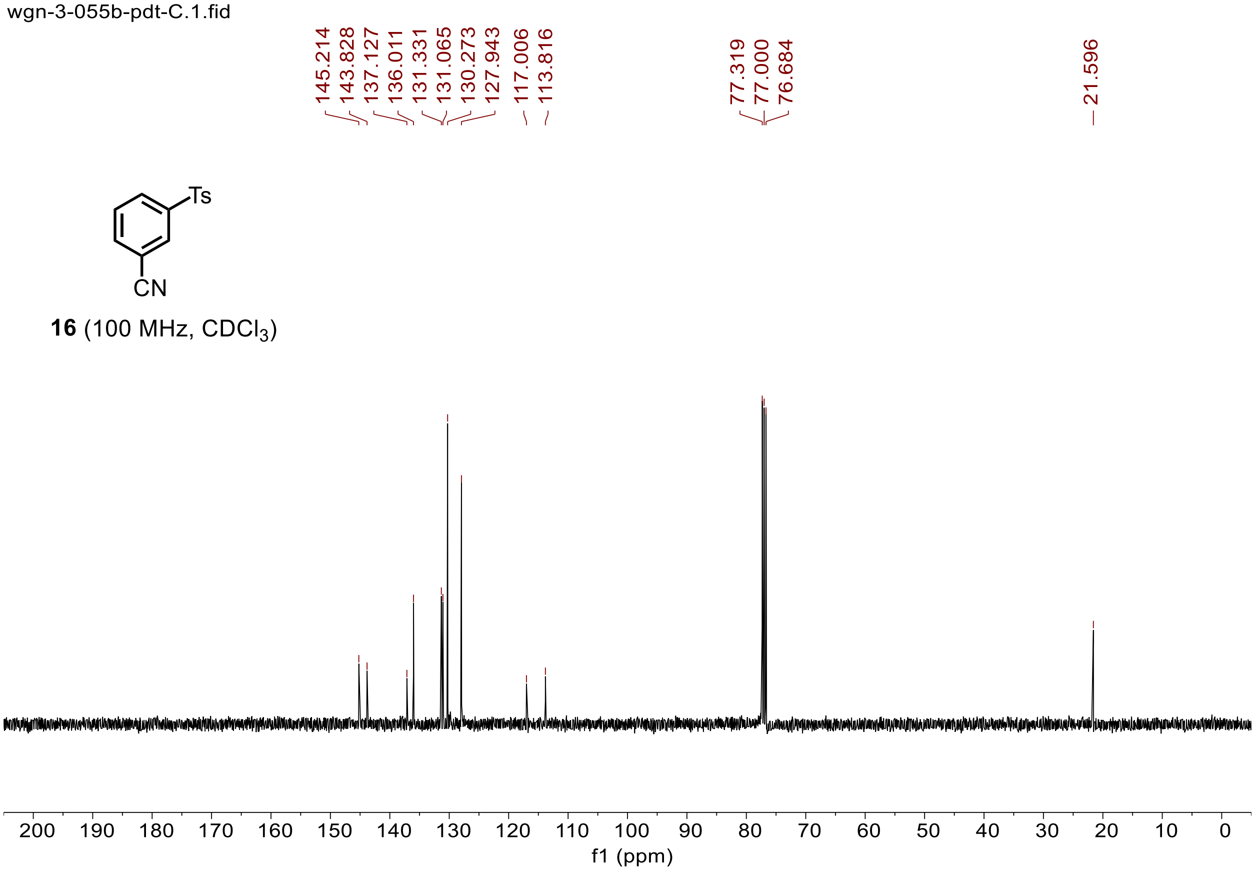

wgn-3-058c-pdt.1.fid

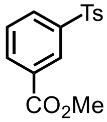

17 (400 MHz, $CDCl_3$)

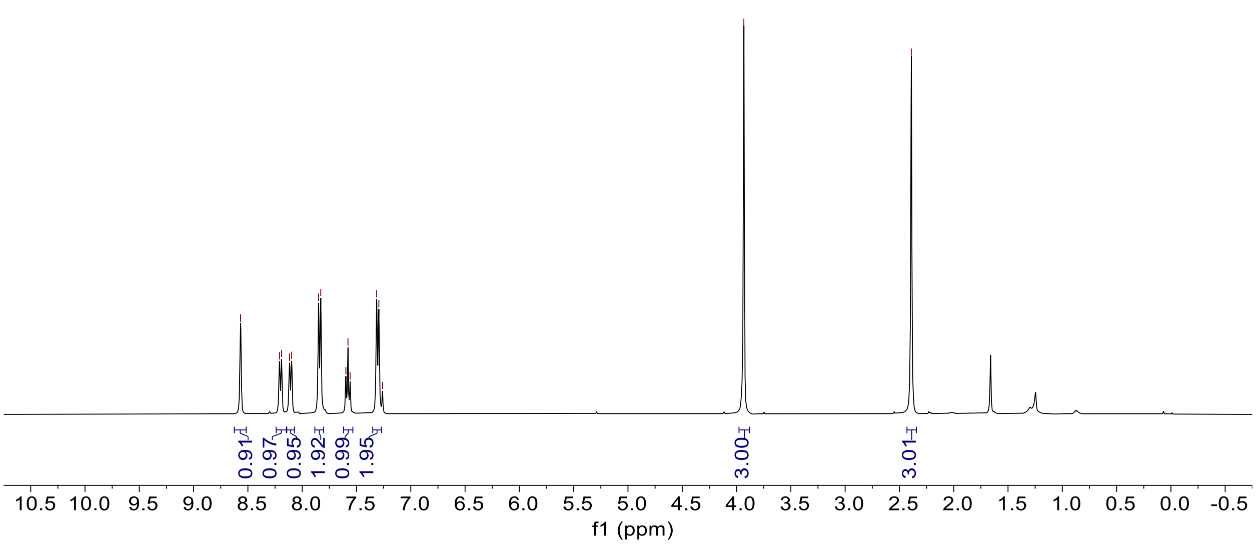

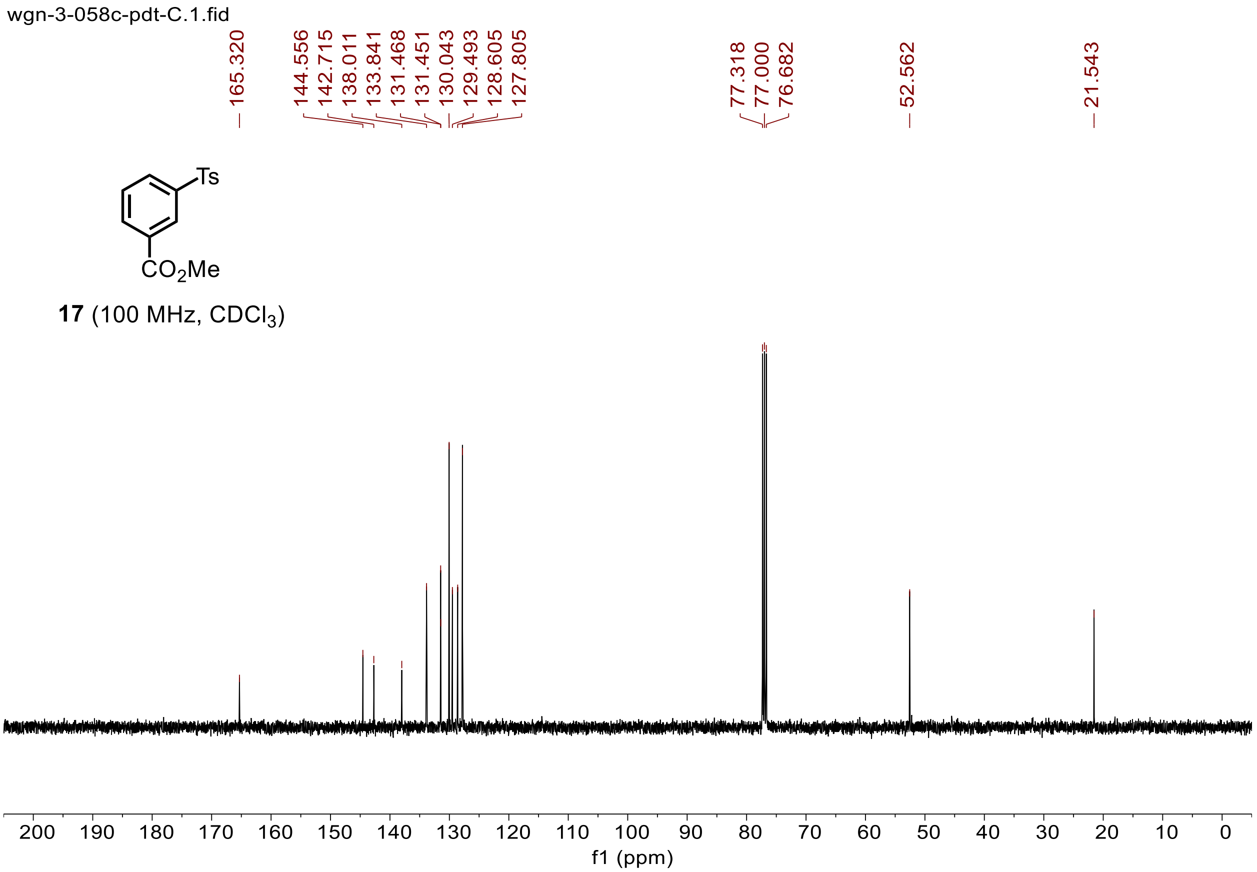

17 (100 MHz, $CDCl_3$)

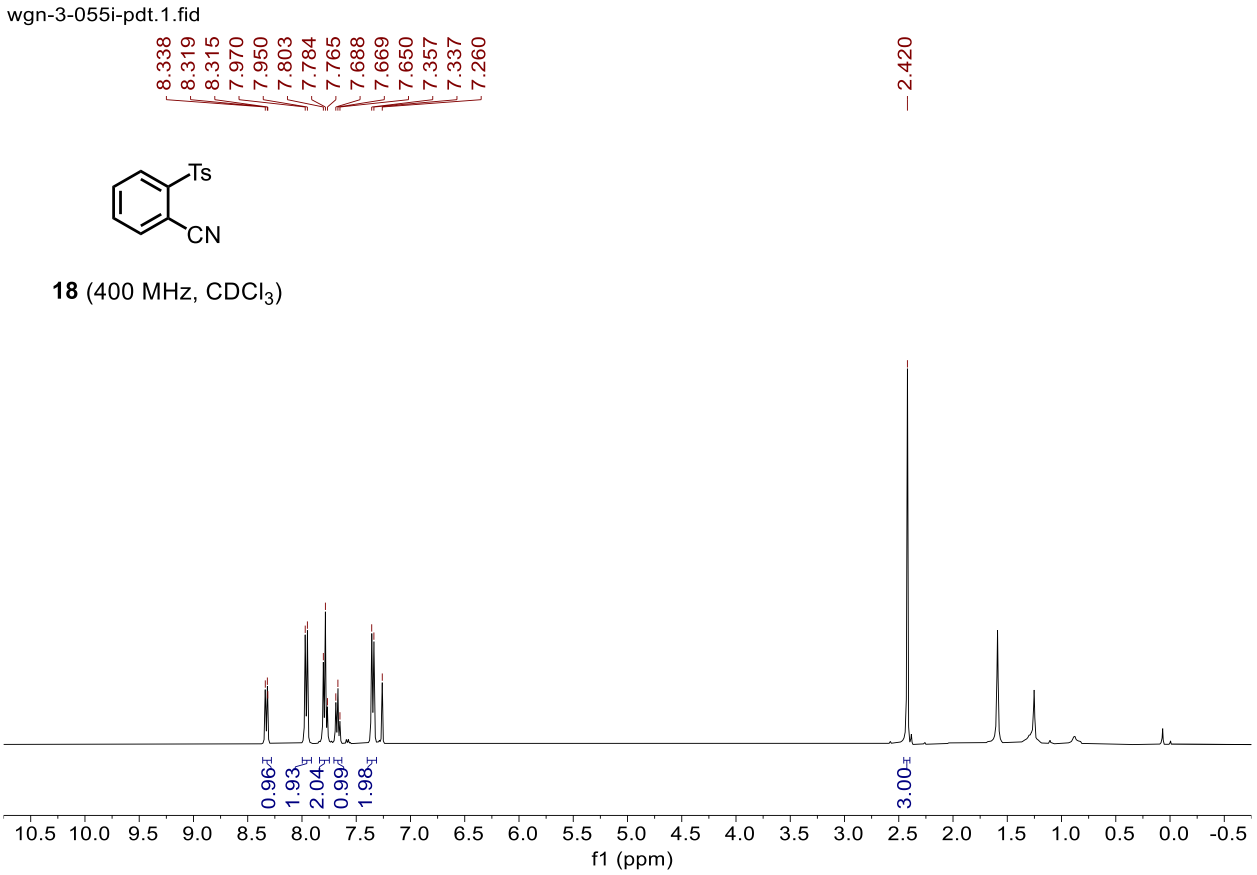

wgn-3-055i-pdt.1.fid
8.338
8.319
8.315
7.970
7.950
7.803
7.784
7.765
7.688
7.669
7.650
7.357
7.337
7.260
2.420
Ts
CN
18 (400 MHz, CDCl3)
0.96
1.93
2.04
0.99
1.98
3.00
10.5 10.0 9.5 9.0 8.5 8.0 7.5 7.0 6.5 6.0 5.5 5.0 4.5 4.0 3.5 3.0 2.5 2.0 1.5 1.0 0.5 0.0 -0.5
f1 (ppm)

wgn-3-055i-pdt-C.1.fid

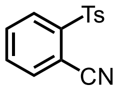

18 (100 MHz, $CDCl_3$)

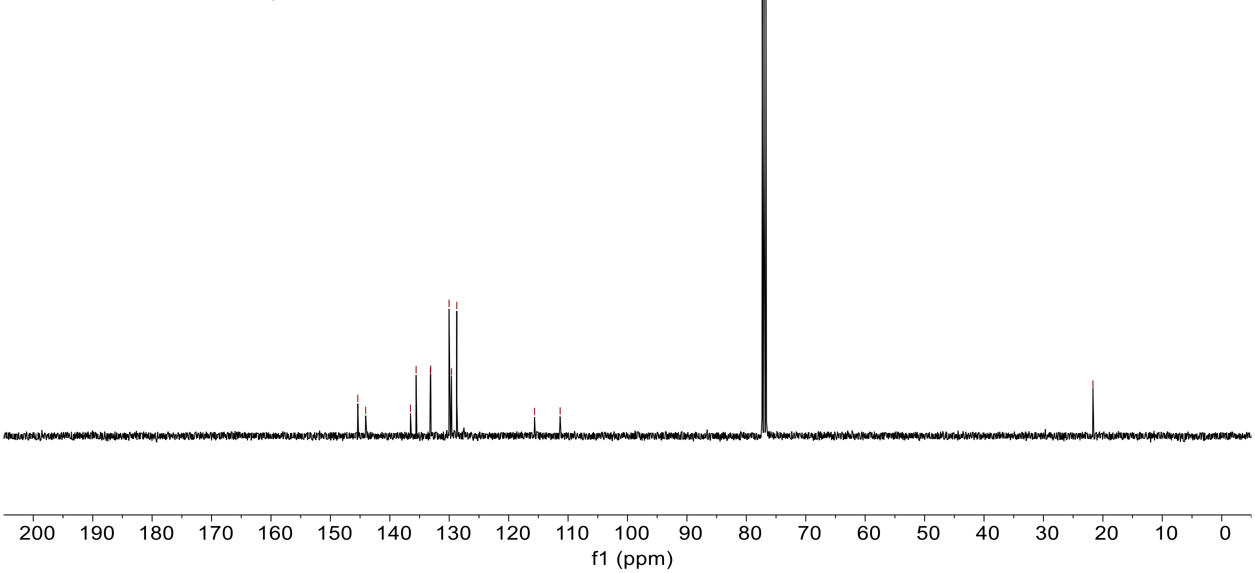

wgn-3-058d-pdt.1.fid

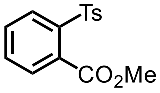

19 (400 MHz, $CDCl_3$)

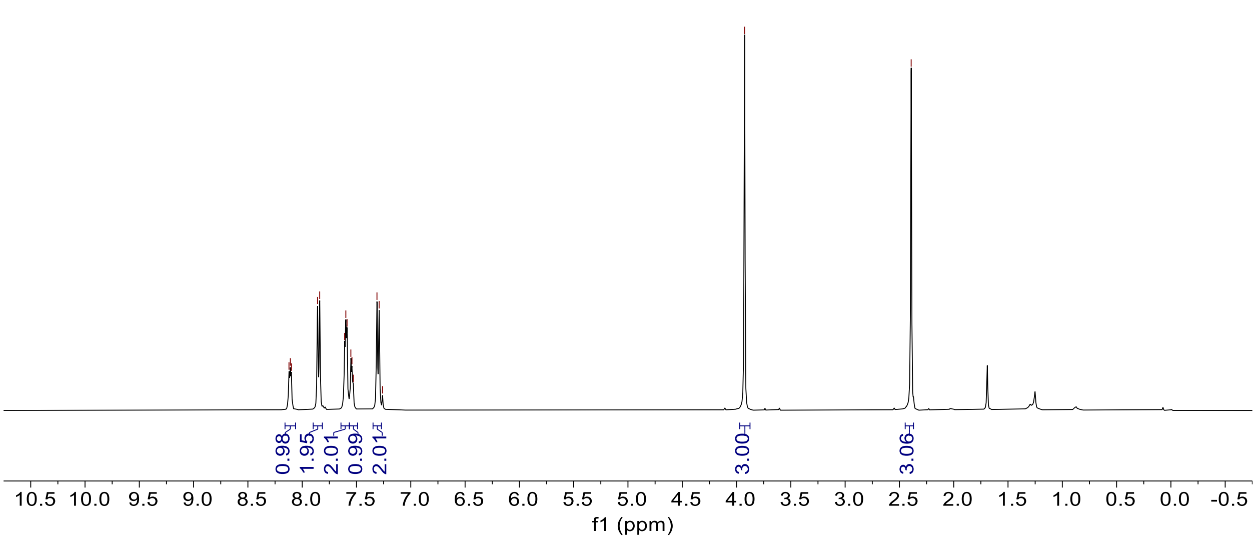

wgn-3-058d-pdt-C.2.fid

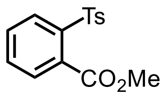

19 (100 MHz, $CDCl_3$)

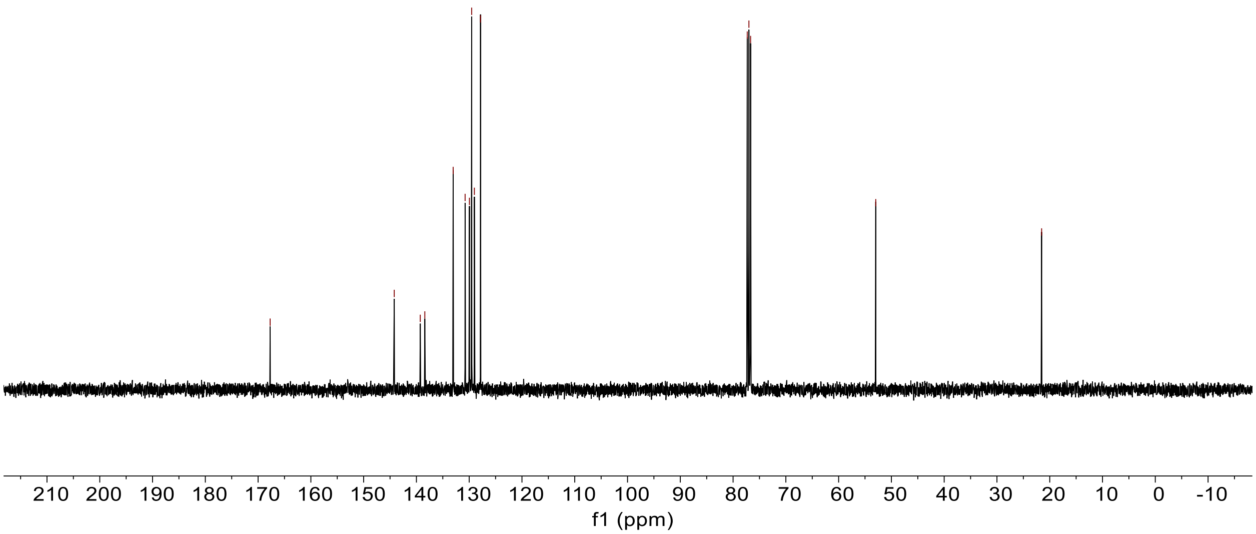

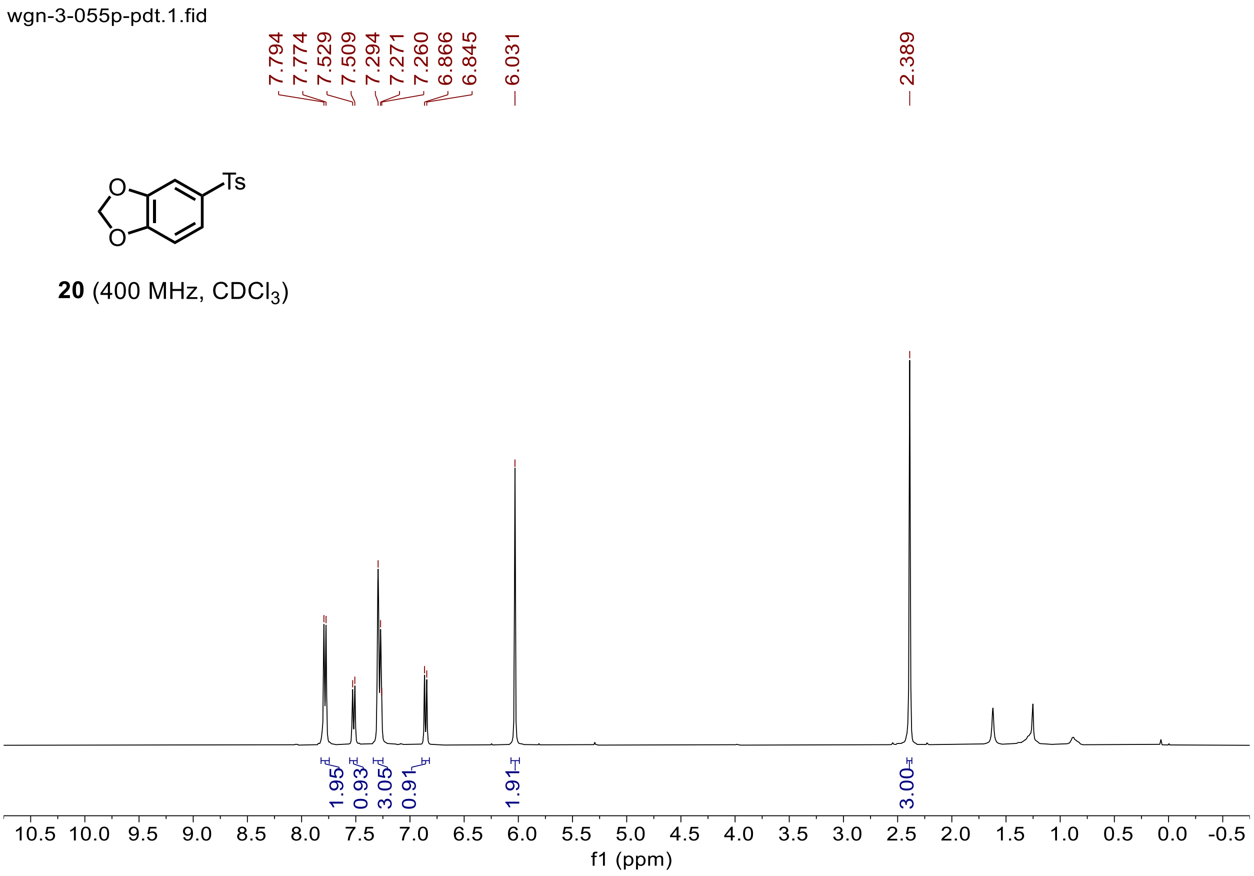
wgn-3-055p-pdt.1.fid
7.794
7.774
7.529
7.509
7.294
7.271
7.260
6.866
6.845
6.031
2.389
Ts
O
O
20 (400 MHz, CDCl3)
1.95
0.93
3.05
0.91
1.91
3.00
10.5 10.0 9.5 9.0 8.5 8.0 7.5 7.0 6.5 6.0 5.5 5.0 4.5 4.0 3.5 3.0 2.5 2.0 1.5 1.0 0.5 0.0 -0.5
f1 (ppm)

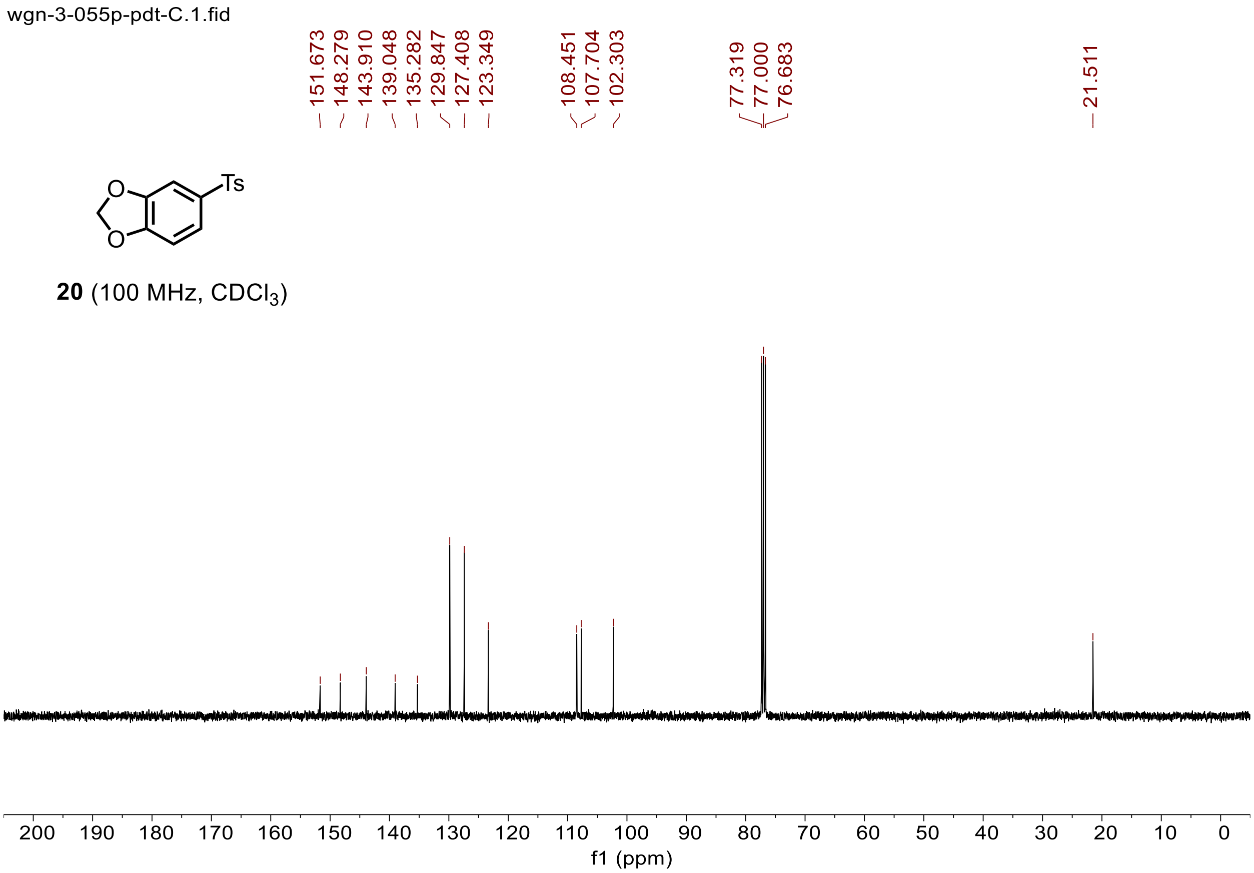
wgn-3-055p-pdt-C.1.fid
151.673
148.279
143.910
139.048
135.282
129.847
127.408
123.349
108.451
107.704
102.303
77.319
77.000
76.683
21.511
Ts
O
O
20 (100 MHz, CDCl3)
200 190 180 170 160 150 140 130 120 110 100 90 80 70 60 50 40 30 20 10 0
f1 (ppm)

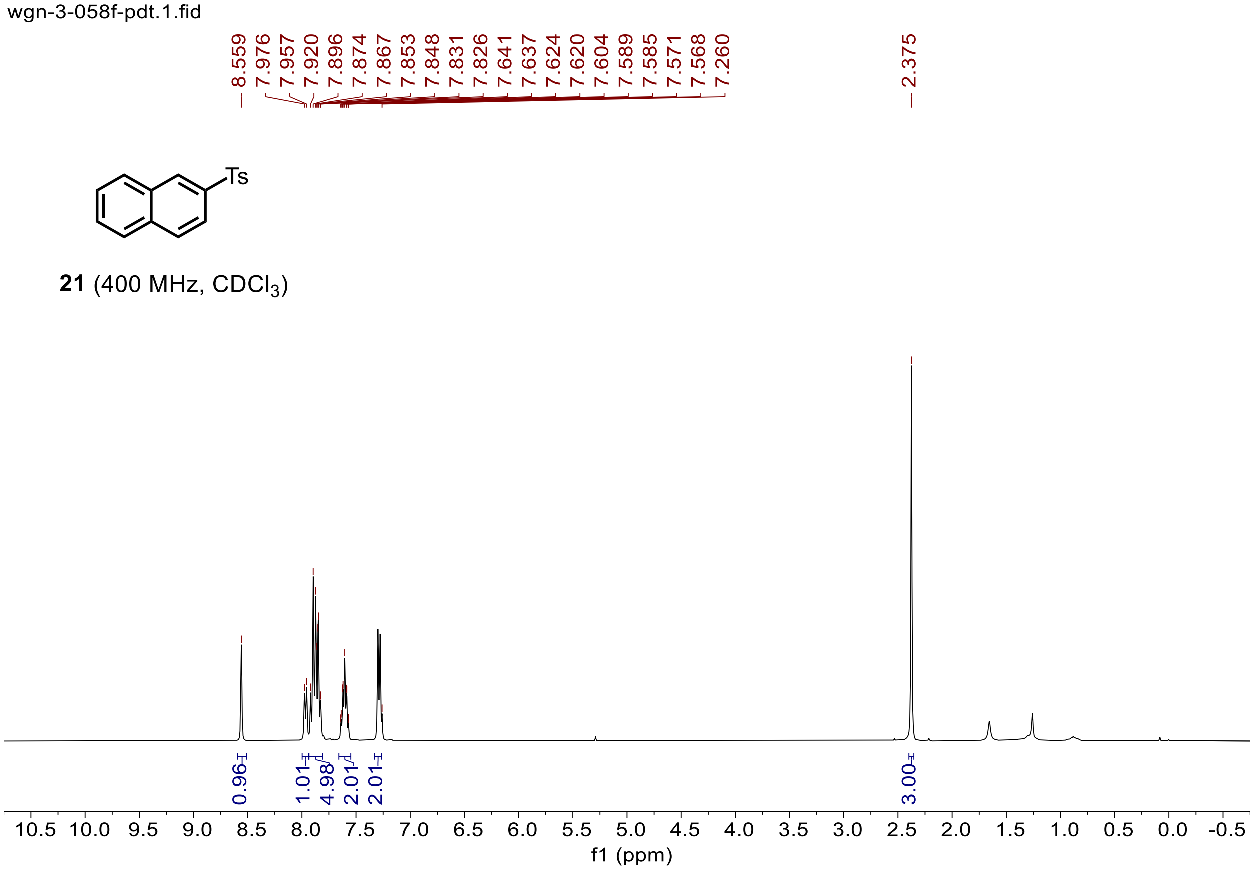
wgn-3-058f-pdt.1.fid
8.559
7.976
7.957
7.920
7.896
7.874
7.867
7.853
7.848
7.831
7.826
7.641
7.637
7.624
7.620
7.604
7.589
7.585
7.571
7.568
7.260
2.375
Ts
21 (400 MHz, $CDCl_3$)
0.96
1.01
4.98
2.01
2.01
3.00
10.5 10.0 9.5 9.0 8.5 8.0 7.5 7.0 6.5 6.0 5.5 5.0 4.5 4.0 3.5 3.0 2.5 2.0 1.5 1.0 0.5 0.0 -0.5
f1 (ppm)

wgn-3-058f-pdt-C.1.fid

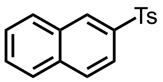

21 (100 MHz, $CDCl_3$)

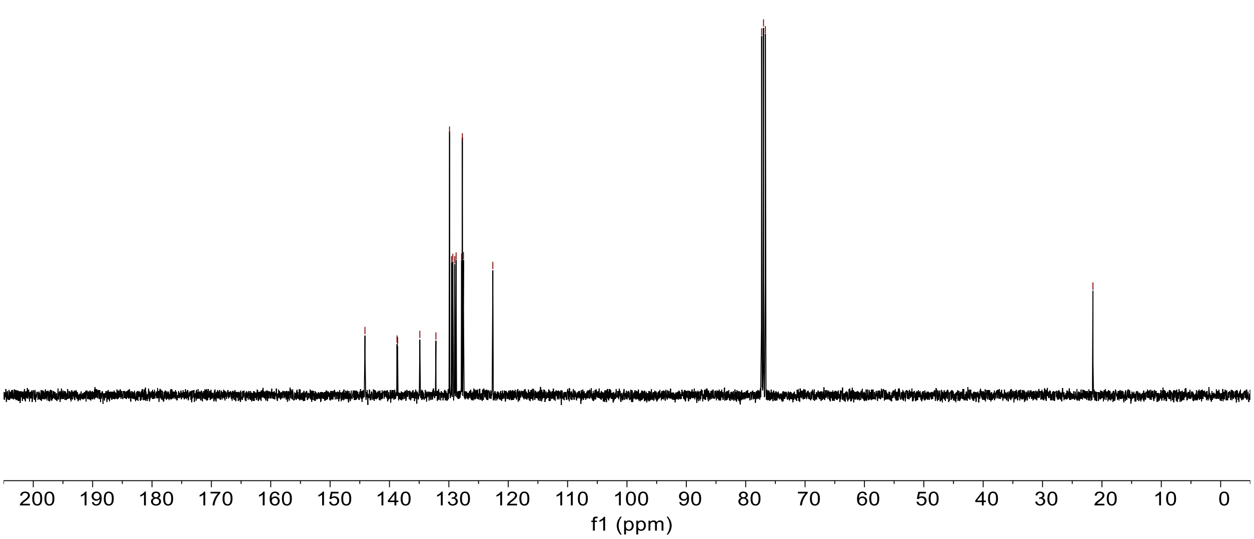

wgn-3-058h-pdt.2.fid

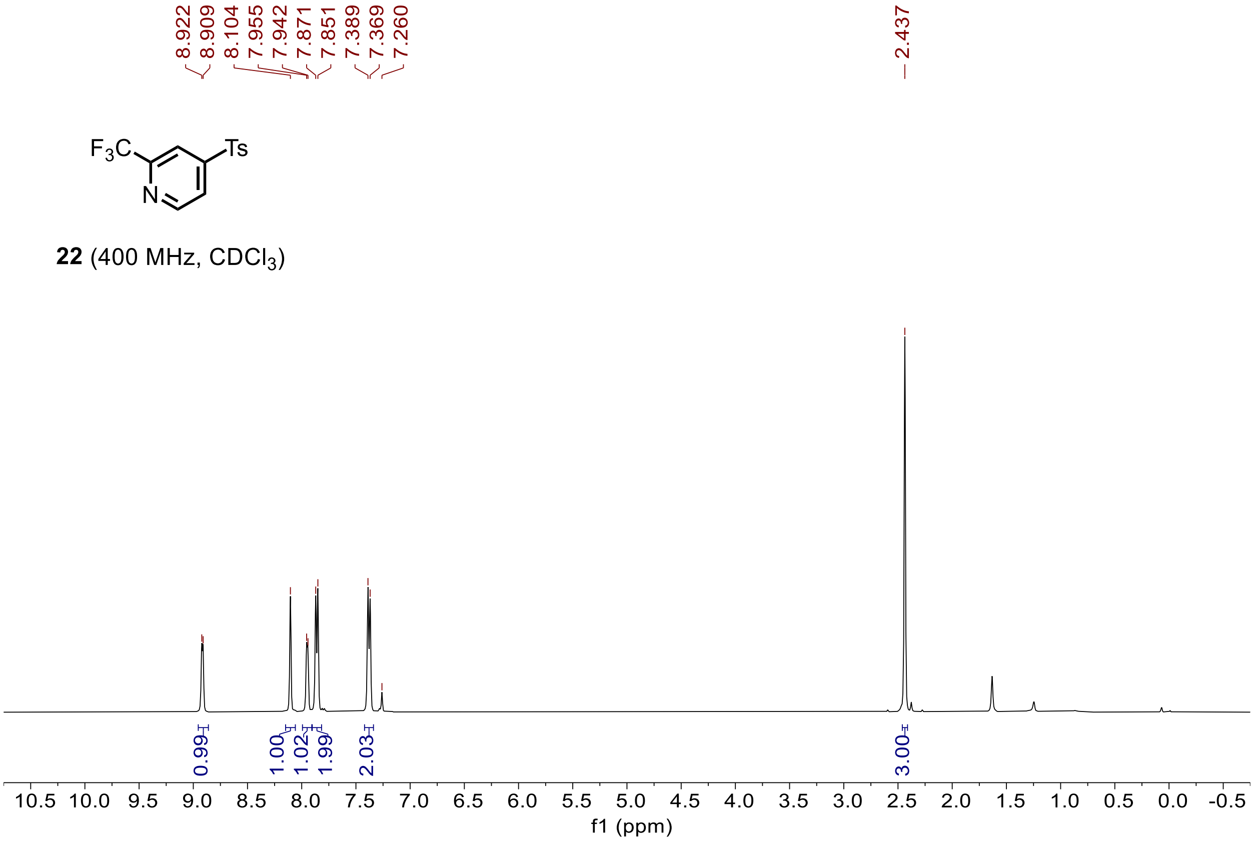

22 (400 MHz, $CDCl_3$)

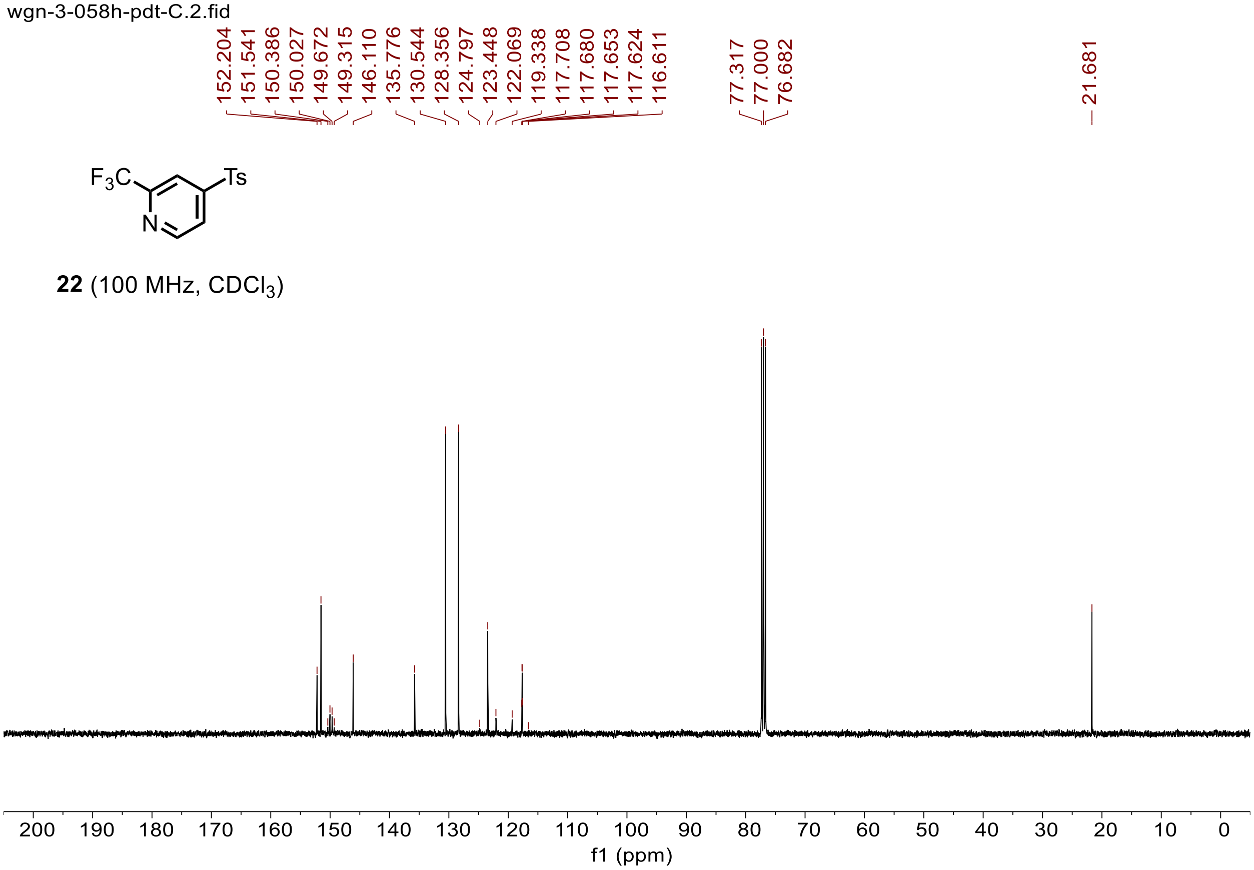

22 (100 MHz, $CDCl_3$)

wgn-3-058h-pdt-F.2.fid

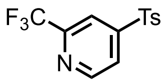

22 (376 MHz, $CDCl_3$)

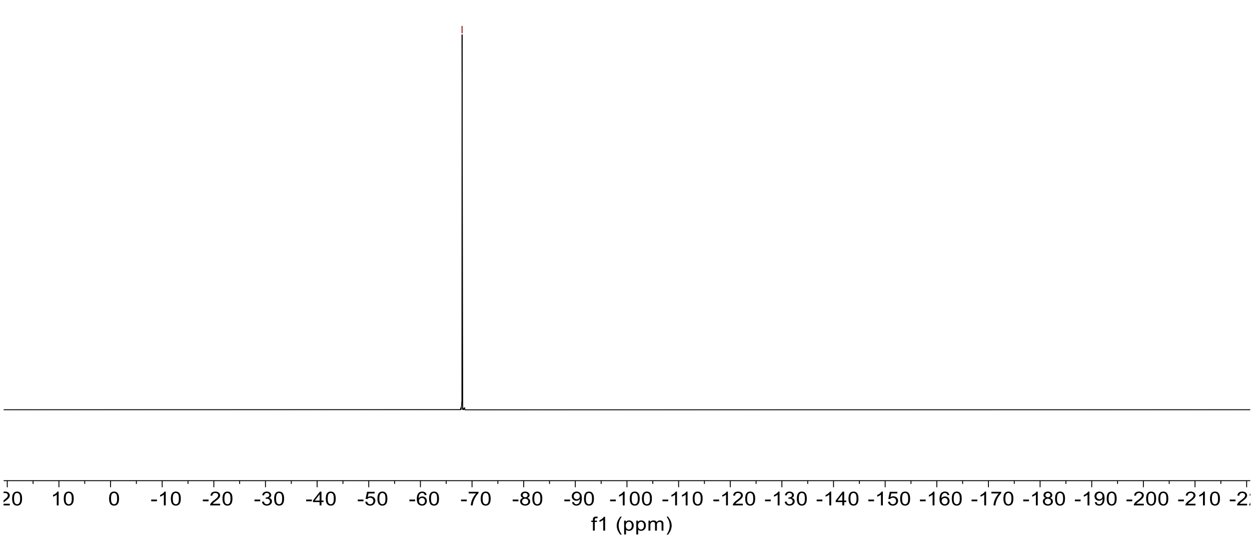

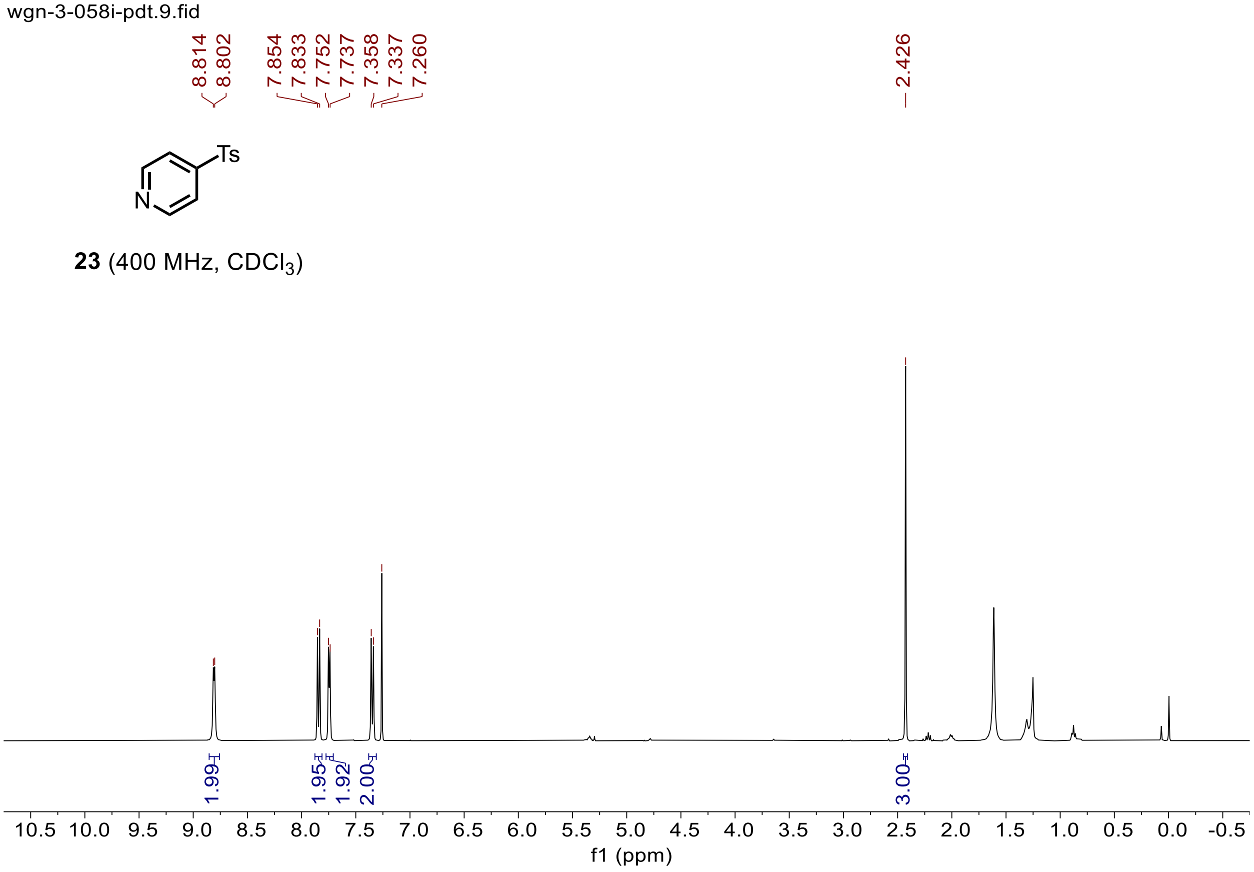
wgn-3-058i-pdt.9.fid
8.814
8.802
7.854
7.833
7.752
7.737
7.358
7.337
7.260
2.426
Ts
N
23 (400 MHz, CDCl3)
1.99
1.95
1.92
2.00
3.00
10.5 10.0 9.5 9.0 8.5 8.0 7.5 7.0 6.5 6.0 5.5 5.0 4.5 4.0 3.5 3.0 2.5 2.0 1.5 1.0 0.5 0.0 -0.5
f1 (ppm)

wgn-3-058i-pdt-C.1.fid

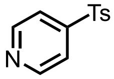

23 (100 MHz, $CDCl_3$)

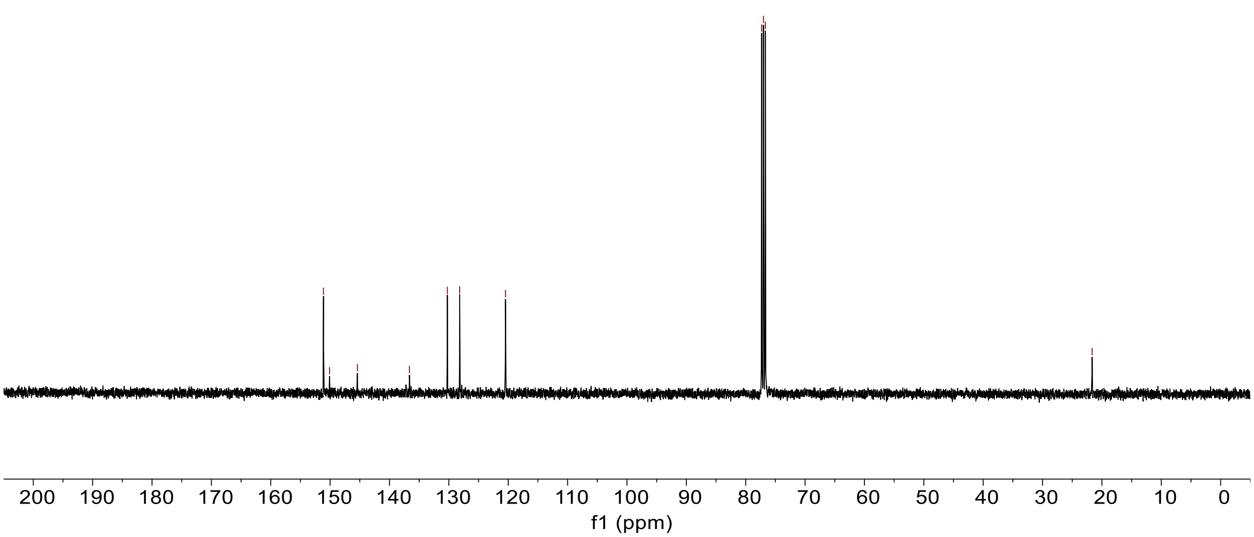

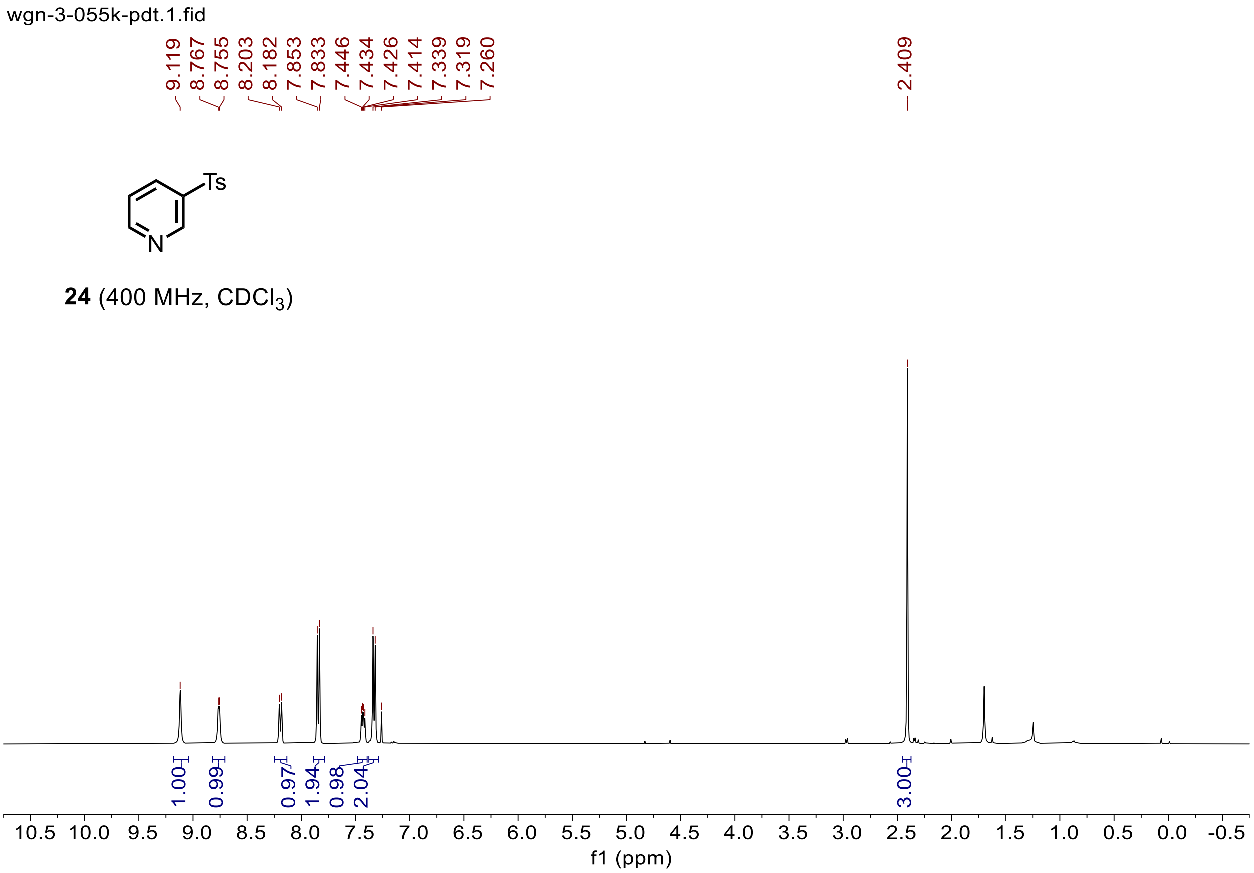

24 (400 MHz, $CDCl_3$)

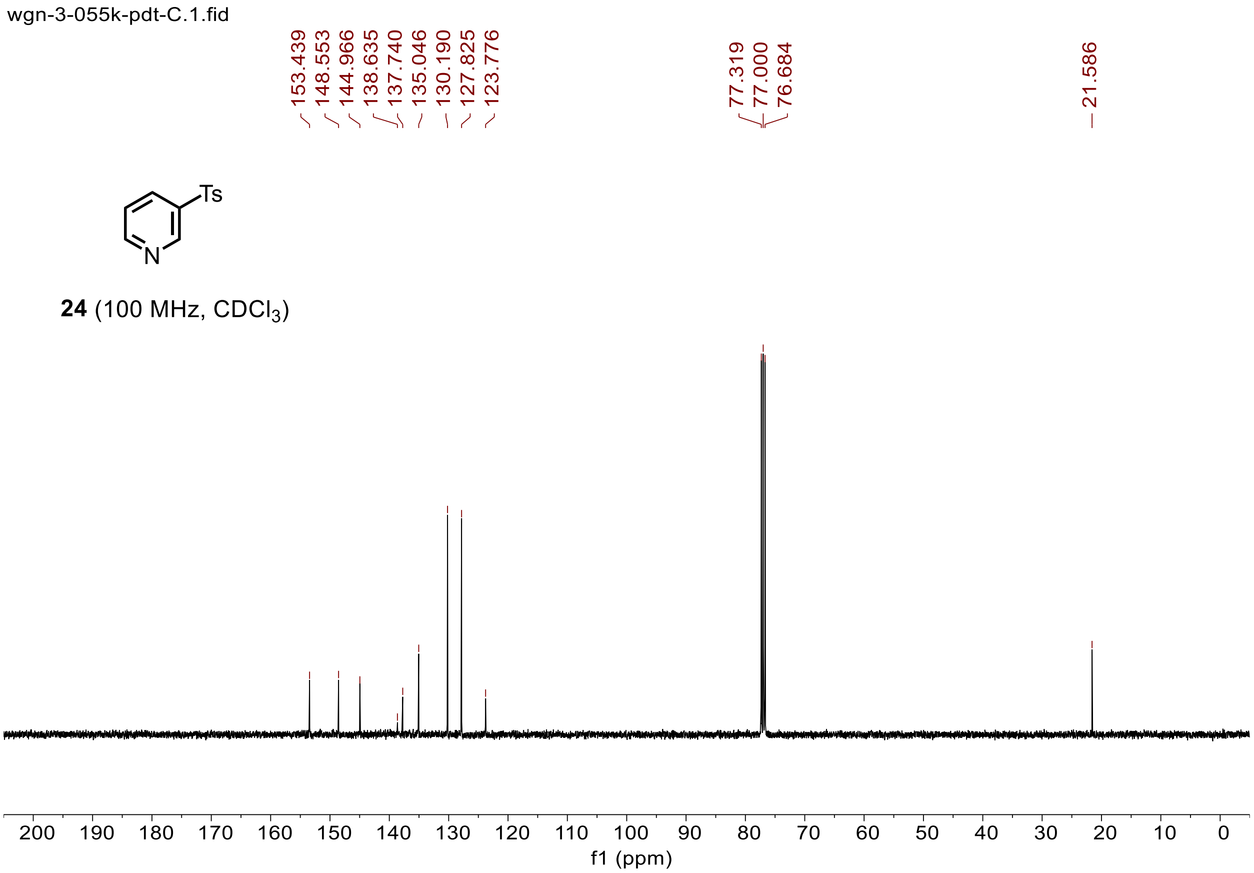
wgn-3-055k-pdt-C.1.fid
153.439
148.553
144.966
138.635
137.740
135.046
130.190
127.825
123.776
77.319
77.000
76.684
21.586
Ts
N
24 (100 MHz, CDCl3)
200 190 180 170 160 150 140 130 120 110 100 90 80 70 60 50 40 30 20 10 0
f1 (ppm)

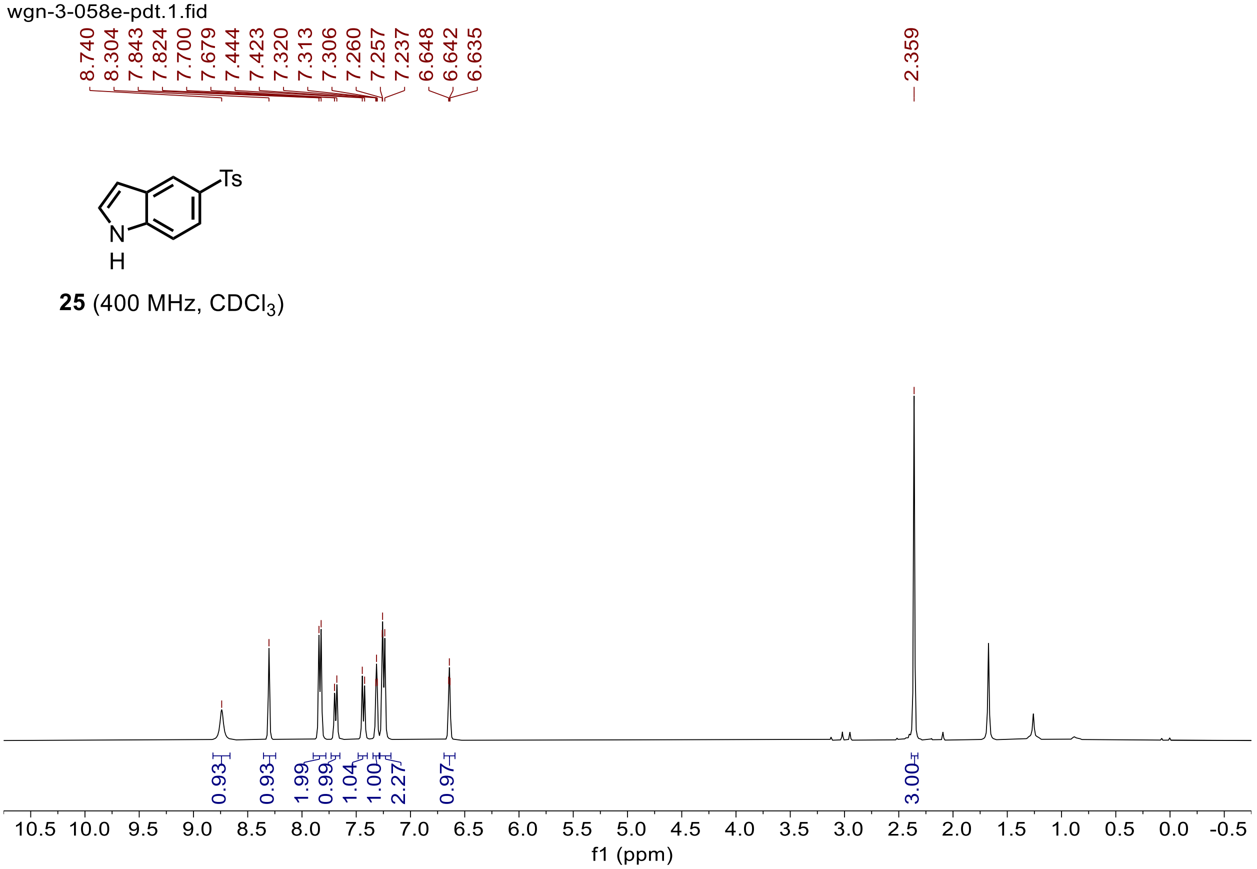
wgn-3-058e-pdt.1.fid
8.740
8.304
7.843
7.824
7.700
7.679
7.444
7.423
7.320
7.313
7.306
7.260
7.257
7.237
6.648
6.642
6.635
2.359
Ts
N
H
25 (400 MHz, CDCl3)
0.93
0.93
1.99
0.99
1.04
1.00
2.27
0.97
3.00
10.5 10.0 9.5 9.0 8.5 8.0 7.5 7.0 6.5 6.0 5.5 5.0 4.5 4.0 3.5 3.0 2.5 2.0 1.5 1.0 0.5 0.0 -0.5
f1 (ppm)

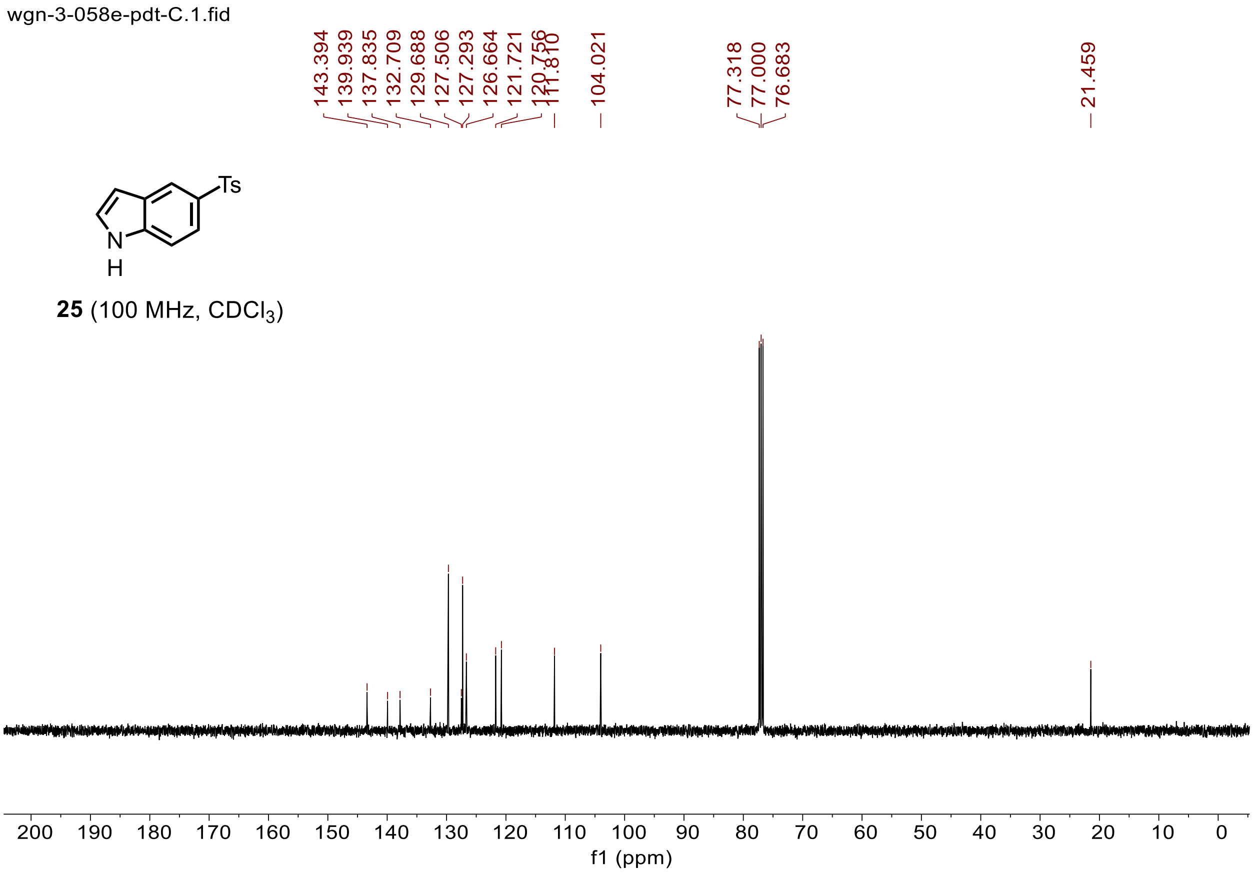
wgn-3-058e-pdt-C.1.fid
143.394
139.939
137.835
132.709
129.688
127.506
127.293
126.664
121.721
120.756
111.810
104.021
77.318
77.000
76.683
21.459
Ts
N
H
25 (100 MHz, CDCl3)
200
190
180
170
160
150
140
130
120
110
100
90
80
70
60
50
40
30
20
10
0
f1 (ppm)

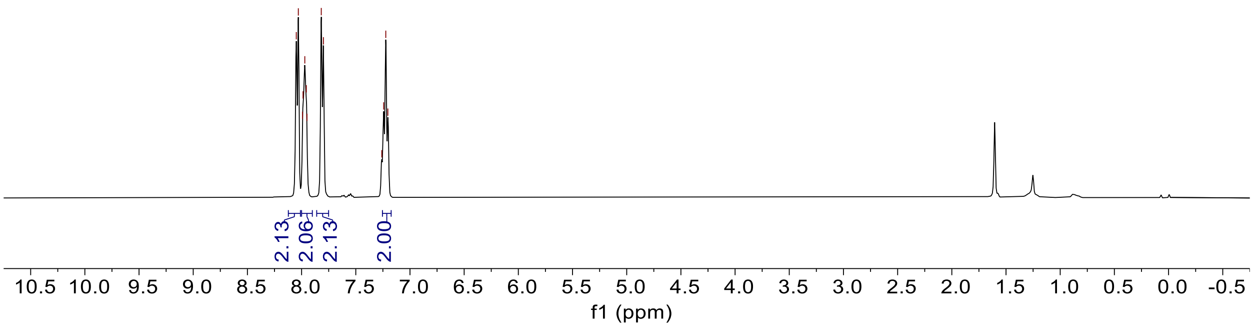

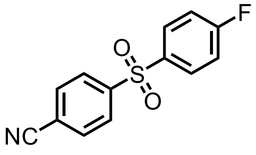

26 (400 MHz, $CDCl_3$)

wgn-3-063b-pdt-C.1.fid

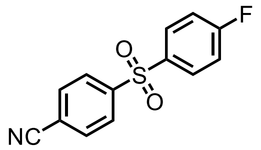

26 (100 MHz, $CDCl_3$)

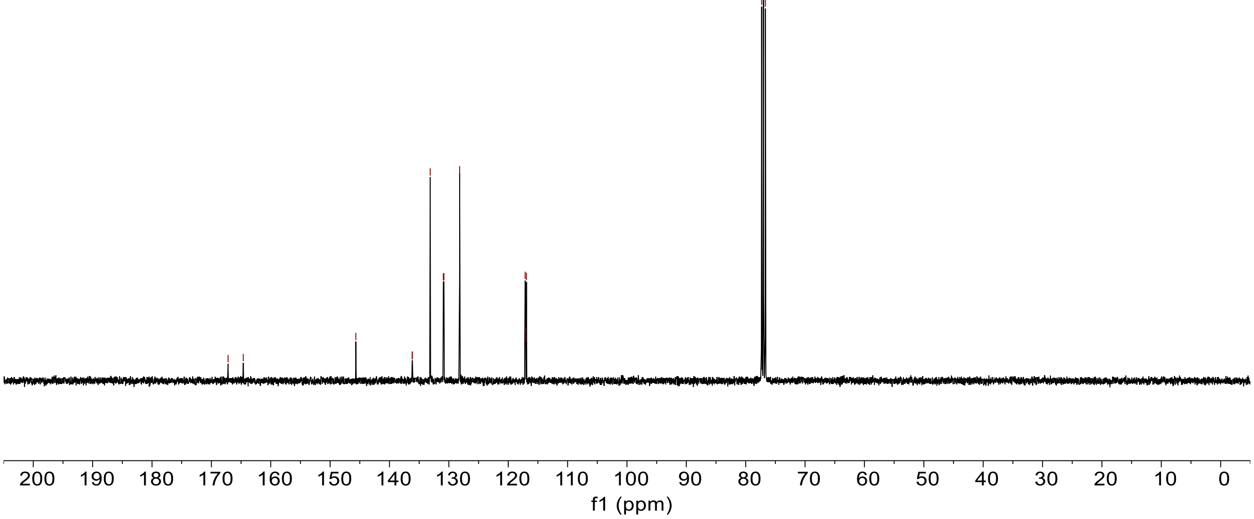

wgn-3-063b-pdt-F.1.fid

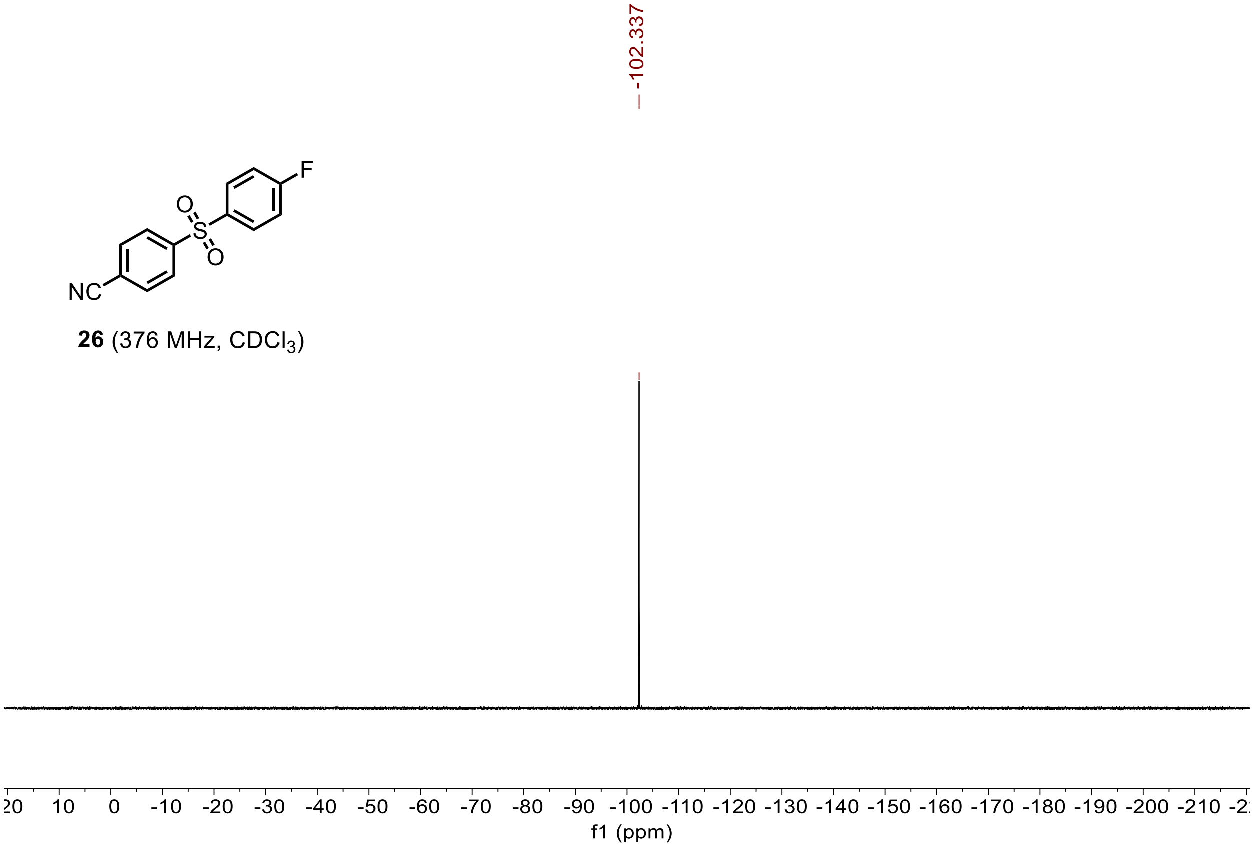

26 (376 MHz, $CDCl_3$)

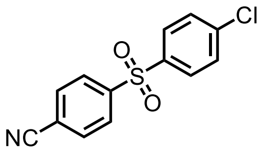

27 (400 MHz, $CDCl_3$)

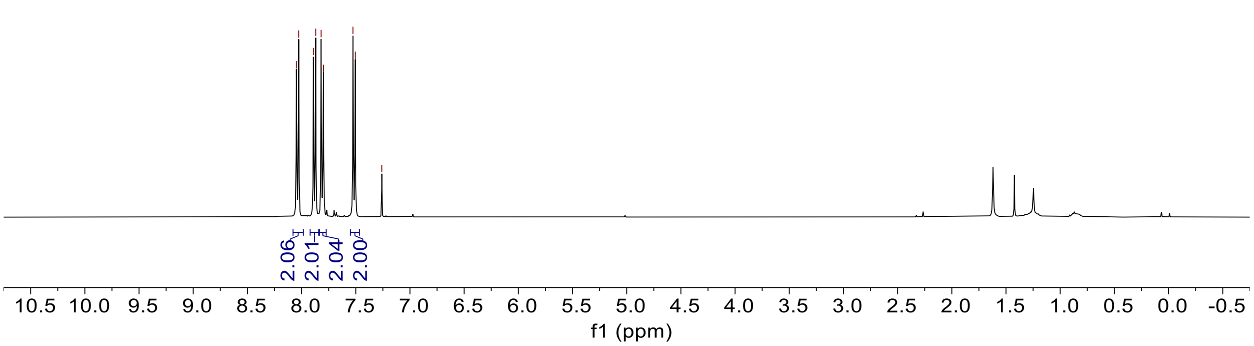

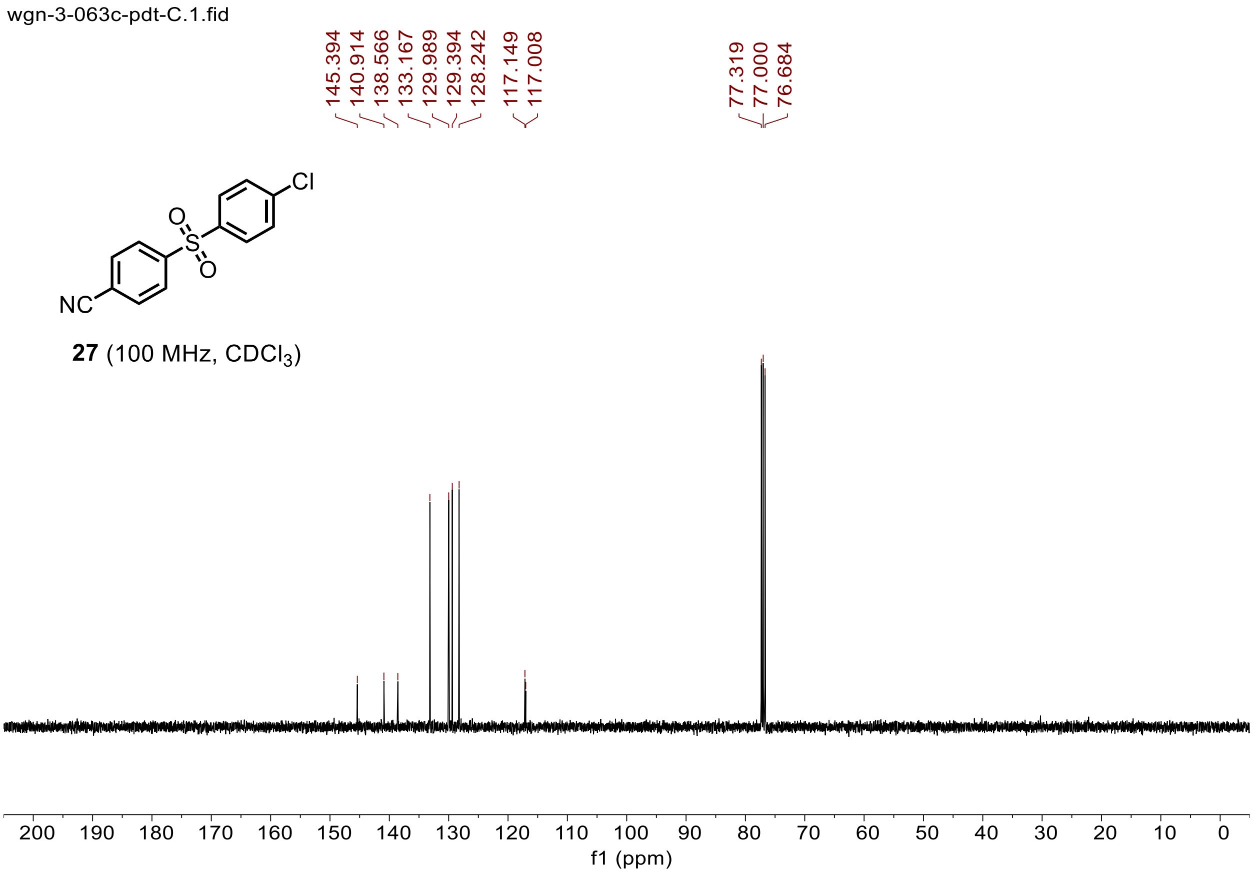

27 (100 MHz, $CDCl_3$)

wgn-3-063a-pdt.1.fid

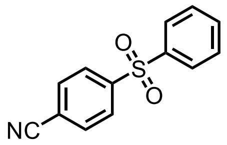

28 (400 MHz, $CDCl_3$)

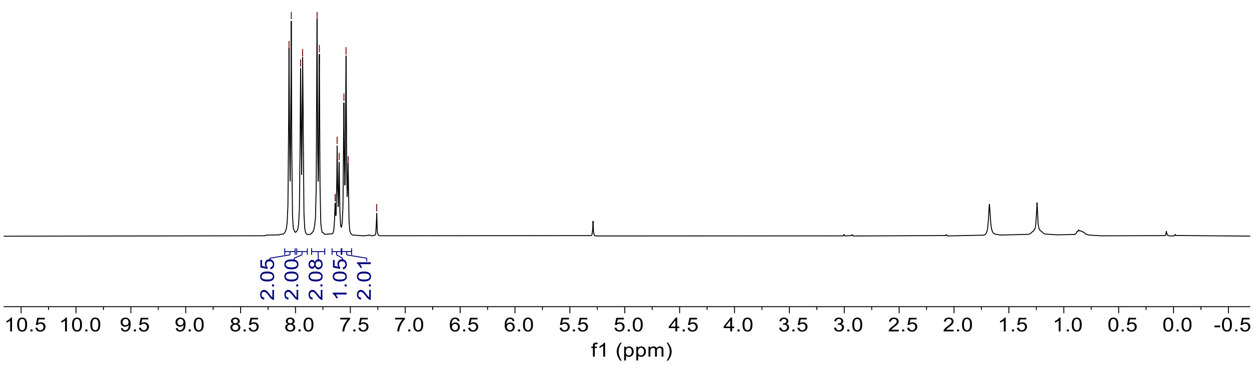

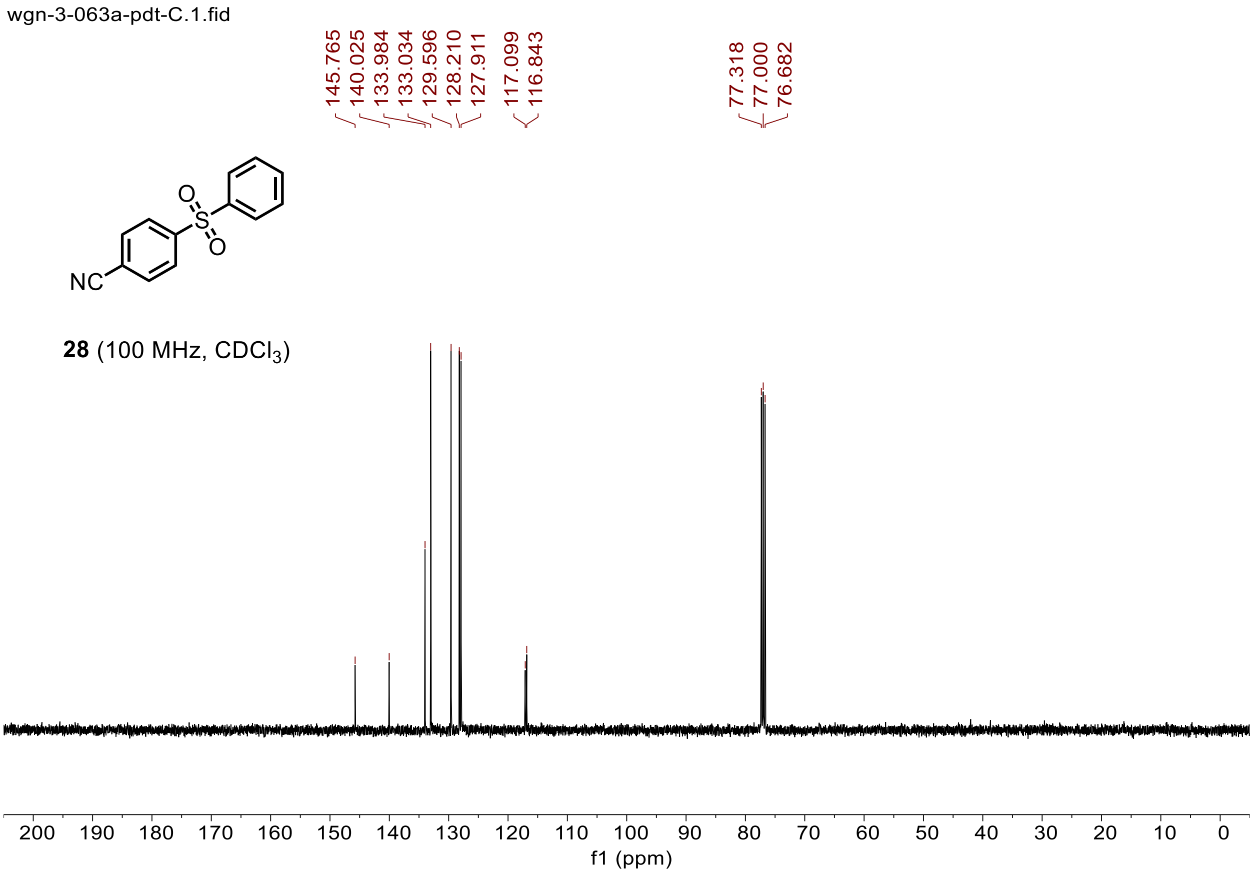

^{13}C NMR (100 MHz, $CDCl_3$) δ 145.8, 140.0, 134.0, 133.0, 129.6, 128.2, 127.9, 117.1, 116.8.

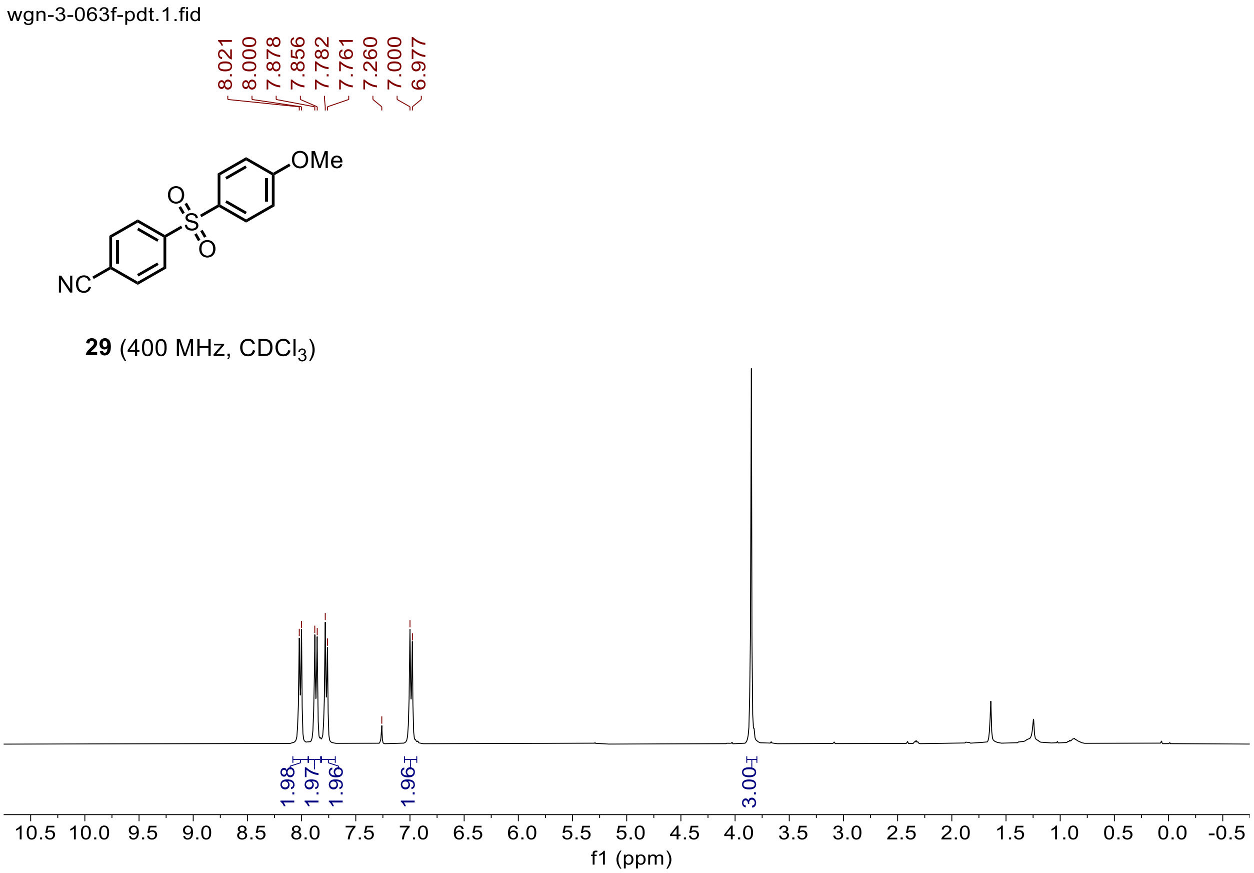
wgn-3-063f-pdt.1.fid
8.021
8.000
7.878
7.856
7.782
7.761
7.260
7.000
6.977
OMe
NC
29 (400 MHz, CDCl3)
1.98
1.97
1.96
1.96
3.00
10.5 10.0 9.5 9.0 8.5 8.0 7.5 7.0 6.5 6.0 5.5 5.0 4.5 4.0 3.5 3.0 2.5 2.0 1.5 1.0 0.5 0.0 -0.5
f1 (ppm)

wgn-3-063f-pdt-C.1.fid

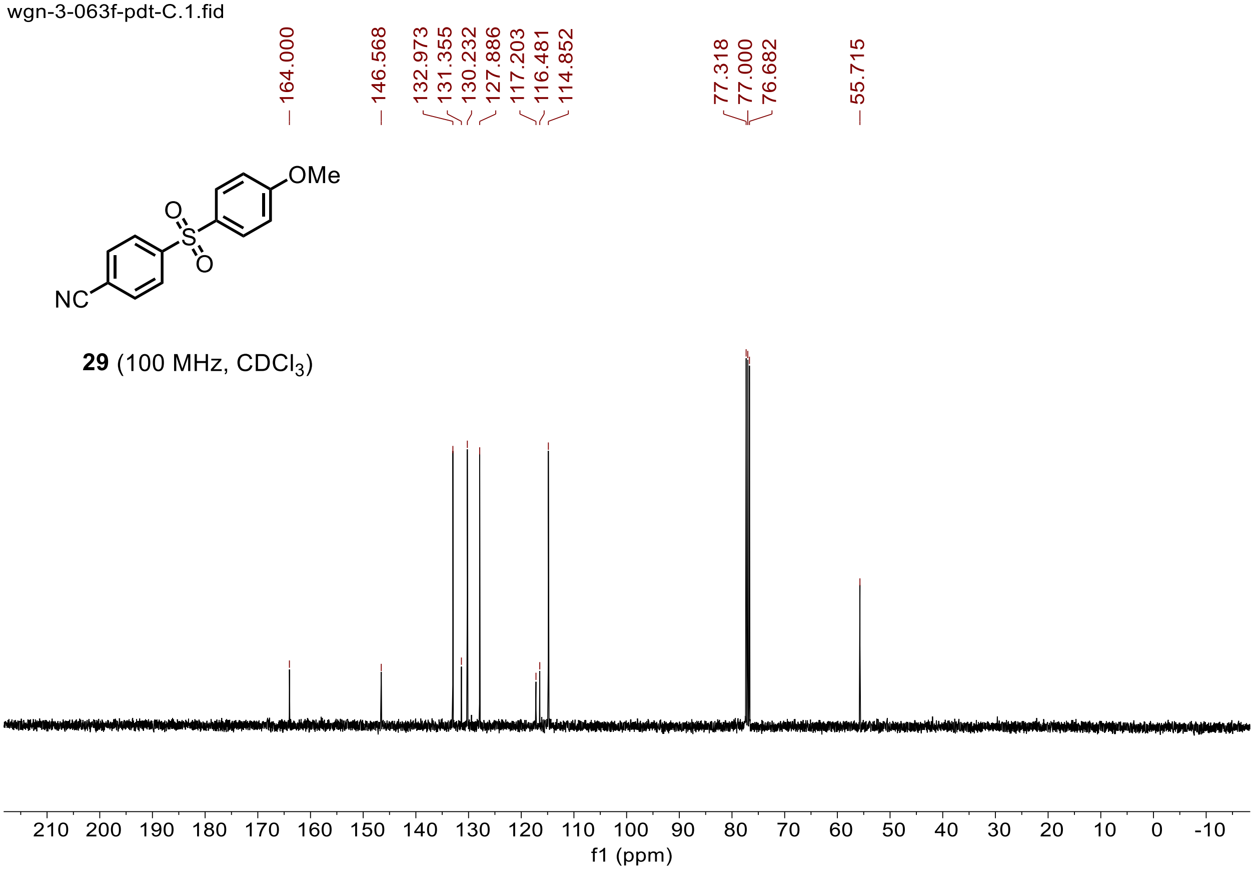

29 (100 MHz, $CDCl_3$)
